# The Toxic Effects of Cigarette Additives. Philip Morris' Project Mix Reconsidered: An Analysis of Documents Released through Litigation

**DOI:** 10.1371/journal.pmed.1001145

**Published:** 2011-12-20

**Authors:** Marcia S. Wertz, Thomas Kyriss, Suman Paranjape, Stanton A. Glantz

**Affiliations:** 1 Center for Tobacco Control Research and Education, University of California San Francisco, San Francisco, California, United States of America; 2 Department of Social and Behavioral Sciences, School of Nursing, University of California San Francisco San Francisco, California, United States of America; 3 Thoracic Surgery, Schillerhoehe Hospital, Gerlingen, Germany; 4 Department of Medicine, University of California San Francisco, San Francisco, California, United States of America; London School of Hygiene & Tropical Medicine, United Kingdom

## Abstract

**Background:**

In 2009, the promulgation of US Food and Drug Administration (FDA) tobacco regulation focused attention on cigarette flavor additives. The tobacco industry had prepared for this eventuality by initiating a research program focusing on additive toxicity. The objective of this study was to analyze Philip Morris' Project MIX as a case study of tobacco industry scientific research being positioned strategically to prevent anticipated tobacco control regulations.

**Methods and Findings:**

We analyzed previously secret tobacco industry documents to identify internal strategies for research on cigarette additives and reanalyzed tobacco industry peer-reviewed published results of this research. We focused on the key group of studies conducted by Phillip Morris in a coordinated effort known as “Project MIX.” Documents showed that Project MIX subsumed the study of various combinations of 333 cigarette additives. In addition to multiple internal reports, this work also led to four peer-reviewed publications (published in 2001). These papers concluded that there was no evidence of substantial toxicity attributable to the cigarette additives studied. Internal documents revealed post hoc changes in analytical protocols after initial statistical findings indicated an additive-associated increase in cigarette toxicity as well as increased total particulate matter (TPM) concentrations in additive-modified cigarette smoke. By expressing the data adjusted by TPM concentration, the published papers obscured this underlying toxicity and particulate increase. The animal toxicology results were based on a small number of rats in each experiment, raising the possibility that the failure to detect statistically significant changes in the end points was due to underpowering the experiments rather than lack of a real effect.

**Conclusion:**

The case study of Project MIX shows tobacco industry scientific research on the use of cigarette additives cannot be taken at face value. The results demonstrate that toxins in cigarette smoke increase substantially when additives are put in cigarettes, including the level of TPM. In particular, regulatory authorities, including the FDA and similar agencies elsewhere, could use the Project MIX data to eliminate the use of these 333 additives (including menthol) from cigarettes.

*Please see later in the article for the Editors' Summary*

## Abstract 2

Stanton Glantz and colleagues analyzed previously secret tobacco industry documents and peer-reviewed published results of Philip Morris' Project MIX about research on cigarette additives, and show that this research on the use of cigarette additives cannot be taken at face value.

## Editors' Summary

**Background:**

The tobacco industry in the United States has recognized that regulation of its products was inevitable as early as 1963 and devoted increasing attention to the likelihood of regulation by the US Food and Drug Administration in the mid-1990s, which finally became law in 2009. In addition, the World Health Organization (WHO) Framework Convention on Tobacco Control (WHO FCTC), which came into force in June 2003, includes provisions addressing the regulation of the contents of tobacco products and the regulation of tobacco product disclosures. Although these steps represent progress in tobacco control, the events of the past few decades show the determination of the tobacco industry to avoid regulation, including the regulation of additives. In the United States, executives of the tobacco company Philip Morris (PM) recognized the inevitability of regulation and responded by initiating efforts to shape legislation and regulation by reorganizing its internal scientific activities and conducting scientific research that could be used to shape any proposed regulations. For example, the company conducted “Project MIX,” a study of chemical constituents in and toxicity of smoke produced by burning cigarettes containing three different combinations of 333 cigarette additives that “were constructed to resemble typical commercial blended cigarettes.” The resulting four papers published *in Food and Chemical Toxicology* in January 2002 concluded that there was no evidence of substantial toxicity attributable to the cigarette additives studied.

**Why Was This Study Done?:**

The use of cigarette additives is an important concern of the WHO, FDA, and similar national regulatory bodies around the world. Philip Morris has used the published Project MIX papers to assert the safety of individual additives and other cigarette companies have done similar studies that reached similar conclusions. In this study, the researchers used documents made public as a result of litigation against the tobacco industry to investigate the origins and design of Project MIX and to conduct their own analyses of the results to assess the reliability of the conclusions in the papers published in *Food and Chemical Toxicology*.

**What Did the Researchers Do and Find?:**

The researchers systematically examined tobacco industry documents in the University of California San Francisco Legacy Tobacco Documents Library (then about 60 million pages made publicly available as a result of litigation) and used an iterative process of searching, analyzing, and refining to identify and review in detail 500 relevant documents.

The researchers found that in the original Project MIX analysis, the published papers obscured findings of toxicity by adjusting the data by total particulate matter (TPM) concentration. When the researchers conducted their own analysis by studying additives per cigarette (as was specified in the original Project MIX protocol), they found that 15 carcinogenic chemicals increased by 20%. The researchers also reported that, for unexplained reasons, Philip Morris deemphasized 19 of the 51 chemicals tested in the presentation of results, including nine that were substantially increased in smoke on a per cigarette basis of additive-added cigarettes, compared to smoke of control cigarettes.

The researchers explored the possibility that the failure of Project MIX to detect statistically significant changes in the toxicity of the smoke from cigarettes containing the additives was due to underpowered experiments rather than lack of a real effect by conducting their own statistical analysis. This analysis suggests that a better powered study would have detected a much broader range of biological effects associated with the additives than was identified in Philip Morris' published paper, suggesting that it substantially underestimated the toxic potential of cigarette smoke and additives.

The researchers also found that *Food and Chemical Toxicology*, the journal in which the four Project MIX papers were published, had an editor and 11 of its International Editorial Board with documented links to the tobacco industry. The scientist and leader of Project MIX Edward Carmines described the process of publication as “an inside job.”

**What Do These Findings Mean?:**

These findings show that the tobacco industry scientific research on the use of cigarette additives cannot be taken at face value: the results demonstrate that toxins in cigarette smoke increase substantially when additives are put in cigarettes. In addition, better powered studies would probably have detected a much broader range of adverse biological effects associated with the additives than identified to those identified in PM's published papers suggesting that the published papers substantially underestimate the toxic potential combination of cigarette smoke and additives.

Regulatory authorities, including the FDA and similar agencies elsewhere who are implementing WHO FCTC, should conduct their own independent analysis of Project MIX data, which, analyzed correctly, could provide a strong evidence base for the elimination of the use of the studied additives (including menthol) in cigarettes on public health grounds.

**Additional Information:**

Please access these Web sites via the online version of this summary at http://dx.doi.org/10.1371/journal.pmed.1001145.

- For *PLoS Medicin*e's own policy on publishing papers sponsored by the tobacco industry see http://www.plosmedicine.org/static/policies.action#funders
- The World Health Organization (WHO) provides information on the Framework Convention on Tobacco Control (FCTC)
- The documents that the researchers reviewed in this paper can be found at the Legacy Tobacco Documents Library

## Introduction

The tobacco industry has been preparing for regulation of its products since at least 1963 when Philip Morris (PM) Vice President of Research and Development Helmut Wakeham expressed concern that the existing US Food and Drug Administration (FDA) “generally recognized as safe (GRAS)” for food additives would not apply to cigarettes and observed that, “Strictly speaking, only published studies could be used to establish a GRAS list for things which are inhaled. Relatively few such studies have been made. This concept would provide a rather formidable barrier in the case of cigarettes” [1]. In 1984 the safety of cigarette additives first became a public issue in the United States [2]. Additives are important elements of tobacco products because they allow manufacturers to modify the sensory and pharmacological properties of tobacco products in a way that affects initiation and cessation (with additives that make the smoke less harsh or more pleasant to the user) or bioavailability and impact of nicotine (through changes in smoke pH or through additives such as menthol) (Box 1) [3],[4]. Regulating additives, including prohibiting their use, poses a serious threat to the tobacco companies. The threat of regulation grew in 1995 when President William Clinton allowed the FDA to regulate cigarettes. While the industry won a Supreme Court ruling that the FDA did not have authority to regulate tobacco products, PM executives recognized by the mid-1990s that regulation was inevitable. They initiated efforts to shape the legislation that would become the 2009 Family Smoking Prevention and Tobacco Control Act [5] granting the FDA authority over tobacco products [6]–[8]. (Among the first actions taken by the newly established FDA Center for Tobacco Products in 2009 was prohibition of flavor additives in cigarettes, with the exceptions of tobacco and menthol [9].) While US legislation over tobacco product regulation was being debated, several tobacco companies developed procedures for assessing the biological effects of new products that delivered nicotine differently than conventional cigarettes [10]. In addition, tobacco product regulation became a global issue with development and implementation of the 2005 World Health Organization (WHO) Framework Convention on Tobacco Control (FCTC) [11], whose articles 9, 10, and 11 require parties to regulate tobacco products.

**Box 1. Excerpt from FCTC implementing guidelines on ingredients (articles 9 and 10) [3, Section 3.1.2.2]**

***Ingredients used to increase palatability***

The harsh and irritating character of tobacco smoke provides a significant barrier to experimentation and initial use. Tobacco industry documents have shown that significant effort has been put into mitigating these unfavourable characteristics. Harshness can be reduced in a variety of ways including: adding various ingredients, eliminating substances with known irritant properties, balancing irritation alongside other significant sensory effects, or altering the chemical properties of tobacco product emissions by adding or removing specific substances.

Some tobacco products contain added sugars and sweeteners. High sugar content improves the palatability of tobacco products to tobacco users. Examples of sugars and sweeteners used in these products include glucose, molasses, honey, and sorbitol.

Masking tobacco smoke harshness with flavours contributes to promoting and sustaining tobacco use. Examples of flavouring substances include benzaldehyde, maltol, menthol, and vanillin.

Spices and herbs can also be used to improve the palatability of tobacco products. Examples include cinnamon, ginger, and mint.

***Recommendation:***

Parties should regulate, by prohibiting or restricting, ingredients that may be used to increase palatability in tobacco products.

Ingredients indispensable for the manufacturing of tobacco products and not linked to attractiveness should be subject to regulation according to national law.

In addition to its political response [6],[7], PM USA reorganized its internal scientific activities to respond to product regulation. In a July 1997 memo “1997 FDA Compliance Preparation,” PM's Vice President of Research and Development stated that, “It is important that we quickly … move to prepare ourselves for these potential regulations” [12]. PM had been analyzing smoke constituents and performing toxicology testing on cigarettes at its European laboratories for decades [13]. In August 1997 PM began to plan and conduct studies of the chemical and toxicological effects of 333 cigarette additives through its “Project MIX” [14]. Project MIX included chemical analysis of smoke, in vitro mutagenicity and cytotoxicity testing, and in vivo rat inhalation toxicology studies. The studies resulted in four papers published together in *Food and Chemical Toxicology* in January 2002 [15]–[18]. The overall conclusion reported by PM in these four papers was that, “The statistically significant changes detected in some of the parameters measured in these studies were considered incidental, without influence on the overall biological effects normally seen with cigarette smoke exposure. There was no indication of any new effects that could be attributable to ingredients” [15].

The use of cigarette additives is an important concern of the FDA. Among the first actions taken by the newly established FDA Center for Tobacco Products in 2009 was prohibition of flavor additives in cigarettes, with the exceptions of tobacco and menthol [9], and in 2011 the FDA's Tobacco Products Scientific Advisory Committee concluded that, “Removal of menthol cigarettes from the marketplace would benefit public health in the United States” [19].

PM has used the published Project MIX papers to assert the safety of individual additives, citing the not yet published papers in a 2001 series of white papers. These papers include *Evaluation of Menthol for Use as a Cigarette Ingredient* [20] and at least seven others (cocoa [21], propylene glycol [22], vanilla extract [23], glycerol [24], sweet orange oil [25], and licorice extract [26]). Lorillard scientist, J. Daniel Heck, a member of the FDA Tobacco Products Scientific Advisory Committee [27] cited Project MIX findings in a 2010 review paper arguing that use of menthol in cigarettes is safe [28].

We used documents made public as a result of litigation against the tobacco industry to investigate the origins and design of Project MIX and conducted additional analyses of the results. This assessment raises questions about the four papers' conclusion that the results “did not demonstrate any meaningful effect of these [333] ingredients on the toxicity of cigarettes” [15].

## Methods

### Tobacco Industry Documents

We systematically examined tobacco industry documents in the University of California San Francisco Legacy Tobacco Documents Library (LTDL; http://legacy.library.ucsf.edu/), which were discovered using established protocols and methods of searching documents based on the snowball technique, by which previous searches inform subsequent searches [29]–[31]. In order to address our research question about how the tobacco industry uses scientific research as a strategy to oppose anticipated tobacco control policy, we began with the search terms “ingredients,” “additives,” and “toxicity.” This initial search yielded thousands of documents, sorted for relevance by the optical character recognition capability of LTDL on the basis of the number of times a search term appears in a document. Screening the documents qualitatively in batches of 50 to 100 helped refine and narrow the search allowing us to collect documents that would help construct a history and provide context for the information that was coming forth. Additional relevant documents were returned by examining adjacent documents (Bates numbers), and searching for key individuals mentioned and locations in which relevant documents were found. The iterative process of searching, analyzing, and refining led to the identification of Project MIX as a key search term. We reviewed approximately 500 relevant documents in detail, including German language documents from the PM-owned European laboratories where Project MIX was conducted. Our analysis is necessarily limited only to those materials (about 60 million pages at the time we conducted this study) that are available as a result of litigation [29].

## Results

### Design Elements

#### Origins of Project MIX

The tobacco companies have a long history of animal research, dating back to at least the 1960s, when they attempted to identify and eliminate the chemicals that caused cancer as part of the effort to develop a “safe cigarette” (see in [32], Chapter 4). Beginning in the late 1970s, PM scientists conducted mouse inhalation and skin painting studies to assess potential cigarette additives, including urea and carmel [33], cocoa [34], and glycerol [35]. In the 1980s PM routinely performed chemical analysis of smoke [36],[37], cytotoxicity testing (Ames and neutral red uptake tests) for mutagenicity [38],[39], and in vivo rodent toxicology studies [40]–[42], honing the techniques for performing these assays at their Institut für Biologische Forschung (INBIFO) in Germany and Contract Research Center (CRC) in Belgium [13].

In March 1994 lawyers representing PM (at the Covington & Burling law firm) commissioned a report on cigarette additives written by six toxicology experts that concluded “ingredients are not hazardous under the conditions of use” [43]. A list of 599 ingredients used in the manufacture of cigarettes was made public for the first time in August 1994 [44] that the industry regarded as “safe” [45]. By 1997 PM joined the Society of Toxicology and the American College of Toxicology to position PM scientists to present and publish results of the INBIFO trials and the six toxicology experts [46].

PM scientists designed Project MIX in 1997 to evaluate the effects of cigarette additives on smoke chemistry, in vitro mutagenicity and cytotoxicity, and in vivo biological activity [14]. In 2001 the four papers written by PM scientists based on the Project MIX results were accepted for publication in *Food and Chemical Toxicology* [15]–[18] through a process that PM scientist and leader of Project MIX Edward Carmines described to coworkers as “an inside job. We went to a journal whose editor knew us” [47].

Carmines' comment is well founded. The then editor of *Food and Chemical Toxicology*, Joseph Borzelleca, was a member of the US tobacco industry's Council for Tobacco Research Scientific Advisory Board [48] and PM Scientific Advisory Board [49] and had a long history of doing contract research and consulting for PM (e.g., see [50] and [51]; there are thousands of documents mentioning Borzelleca in LTDL). The associate editor, P.J. van Bladeren, was coauthor of a paper at the 1991 meeting sponsored by Indoor Air International, a group managed by tobacco industry lawyers, “International Conference: Priorities for Indoor Air Research and Action” [52] that served as the launch for a nominally peer-reviewed journal that could be used to publish research supporting the tobacco industry's position on secondhand smoke [53]. Susan Barlow, one of two review editors, coauthored a PM-funded review paper that, after incorporating comments from PM, questioned the evidence linking secondhand smoke and sudden infant death syndrome [54]. Eleven of the journal's International Editorial Board members had ties to the tobacco industry: three were employees (A.W. Hayes, D.J. Doolittle [55]–[57], and Y.P. Dragan [58]–[60]; two held positions on PM Scientific Advisory Board (M. Pariza and S.L. Taylor [61]); and six others had tobacco industry funding or other connections (O.I. Aruoma [62],[63], A.R. Boobis [64]–[66], H.R. Glatt [67]–[69], G.C. Hard [70],[71], B. Pool-Zobel [72],[73],and H. Poulsen [74]–[77]).

#### Additive selection

Project MIX examined the potential chemical and biological effects of 333 additives used in cigarette manufacturing, selected because they “are representative of flavors used throughout the world by Philip Morris” [78]. The 333 additives represent some of the 599 that had been reported to be added in cigarette manufacturing [44].

The 333 additives selected for testing under Project MIX were divided into three overlapping “ingredient groups” [15]. The additives were added to tobacco during the cigarette manufacturing process to produce the test cigarettes. The control cigarette contained tobacco only. Two target levels for each additive, a low level that “approximated the level considered to be reflective of those used in commercial cigarettes” [15] and a high level, 1.5 or 3 times multiple of the low level. (The exception was menthol in ingredient group 3, which was only studied at a single level, 18,000 parts per million [ppm], in both the low and high groups “because no more material could be physically incorporated into the test cigarette” [15].) Carmines, et al. state in the published paper [15] that they could only vary the levels by a factor of 1.5–3 because it was the maximum amount of the ingredients that the cigarettes would hold and still “burn in manner similar to the control cigarette.” Tobacco was removed from the test cigarettes to keep the cigarette mass constant.

The specific contents of the test cigarettes are listed in tabular form in the published paper [15] and can be summarized as follows: ingredient group 1, 177 additives; ingredient group 2, 277 additives (including 117 also in group 1); ingredient group 3, mostly menthol plus cocoa shells, licorice extract, and corn syrup.

The process or logic for assigning additives to groups is not evident from the industry documents or published papers. It is not clear how or why PM selected these 333 additives and the specific amounts or combinations used, nor why the other 266 were not selected for testing in Project MIX. The published paper [15] simply states that the additives are “representative of ingredients [additives] used throughout the world,” are “food-grade quality or better,” and were added during the test cigarette manufacturing process “to closely mimic the actual process” that “were constructed to resemble typical commercial blended cigarettes.”

To prepare for Project MIX, PM USA manufactured seven test cigarettes under these product codes: 97.MG.292 for control, 97.MG.295 and 97.MG.296 for ingredient group 1 low and high, 97.MJ.125 and 97.MJ.126 for ingredient group 2 low and high, and 97.MJ.127 and 97.MJ.128 for ingredient group 3 low and high [79]. The test cigarettes were released to INBIFO in January 1998 by PM's US Product Testing Laboratory scientist after performance tests were completed [80].

#### Chemical analysis of the smoke

INBIFO measured 51 mainstream smoke constituents in the Project MIX test cigarettes. According to Carmines, these constituents were selected from lists prepared by the US Consumer Product Safety Commission (CPSC) [81] and International Agency for Research on Cancer [16] (IARC) monograph 38 [82]. A 1997 memo written by PM scientist Rick Solana [83],[84] indicates that PM had created a list of 39 as a “current smoke constituent chemistry list for analyzing mainstream smoke in product integrity testing” on the basis of the IARC and CPSC lists screened against the 1994 National Toxicology Program (NTP) carcinogen list.

We could not determine how the 51 constituents were selected for use in Project MIX. The published paper from Project MIX [16] acknowledges the selection was “to a certain degree subjective and not based on extensive risk assessments,” and argues that some analytes were not included because “there is no straightforward toxicological interpretation for changes that might occur” or because “their method development is still in progress” [16]. Combining the CPSC and IARC lists yields 118 compounds (Text S1, Table S-1). Solana noted (in 1997, of the 39 constituent list), that “polyaromatic hydrocarbons…will be kept on the current list this is due to the focus on this class of compounds. Even these compounds, however, might be considered for discontinuing should they prove to consistently track the polyaromatic hydrocarbons which are on the recommended list” [83]. This decision could account for the omission of some constituents from Project MIX.

The list of 72 constituents not measured in Project MIX includes 11 polycyclic aromatic hydrocarbons (PAHs). PAHs are of particular concern because they cause carcinogenic and noncarcinogenic disease in animals and in humans [85]–[87]. Project MIX also included eight chemicals not on the CPSC, IARC, or National Toxicology Program (NTP) lists: three PAHs: acenaphthene, acenaphthylene, benzo[ghi]perylene; naphthalene; m-, o-, and p-cresol; and particle size distribution. One of these PAHs was also identified in a single ingredient smoke analysis study, conducted by PM under the code name URSUS in 1994 [88].

#### Omission of ammonia results

**Figure 1:**
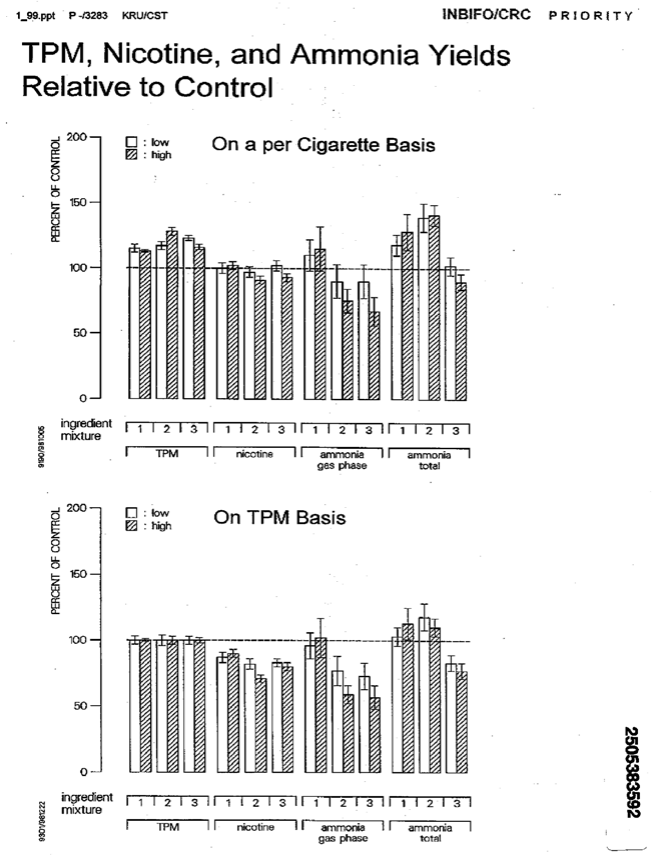
This result from an internal INBIFO report dated January 1999 shows results for measuring ammonia in Project MIX [**89**]. (Error bars are SD.) All three ingredient groups were associated with increased TPM, higher gas phase ammonia for ingredient group 1 and lower levels for ingredient groups 2 and 3 (in a dose-dependent manner), reported as statistically significant [90]. Total ammonia was increased in ingredient groups 1 and 2. Because cigarettes with the additives produced more TPM, normalizing the ammonia produced by TPM lowered (and in most cases reversed) the estimated effects of the ingredients on ammonia production.

Project MIX assessed ammonia levels in the smoke of the test cigarettes, yet the results were not included in the published report [16]. Internal reports from early 1999 [89],[90] show that ammonia was analyzed and significantly elevated in the smoke from ingredient group 1 high level and ingredient group 2 low and high levels, and significantly decreased in ingredient group 3 (containing menthol) low and high levels, compared to control cigarette smoke (Figure 1). The omission of ammonia test results from the final reports is curious in light of the fact that ammonia increases the pH of tobacco smoke making it less acidic and therefore easier to smoke [91] while increasing the bioavailability of the nicotine present in the smoke [92],[93]. No pH test results for the Project MIX test cigarettes were found.

### Presentation of Results: Normalizing by Total Particulate Matter

In October 1998, “at the request of the client [PM USA]” [94], INBIFO scientists amended the Project MIX study protocol to report and analyze results on a per total particulate matter (TPM) basis; the original January 1998 plan [95] called for results to be presented on a per cigarette basis. The change was made 8 mo after INBIFO scientist Wolf Reininghaus reported to PM USA Manager of External Studies Gerry Nixon [96],[97] that the Project MIX “prototypes differ from control in a 10% higher TPM yield (which is compensated for by dilution in the subchronic study), [and] a 20% higher acrolein yield (which is, after compensation for TPM, no relevant difference)” [96].

The published paper does include a table reporting the smoke chemistry results on a per cigarette basis [16]; these data showed that all the test cigarettes containing any additives produced more TPM than the control cigarettes (ingredient group 1 low and high levels were 15% and 13% higher, ingredient group 2, 17% and 28%, and ingredient group 3, 13% and 16%, higher). Rather than emphasizing the biological importance of this increase in TPM, PM's researchers normalized the results by TPM and discussed whether the additives increased the amounts of each toxin in the smoke per unit TPM produced compared to the control cigarettes. Thus, as long as the amount of a toxin in the smoke of a test cigarette increased by less than the amount TPM increased in that cigarette, the ratio would drop even if both the toxin and TPM increased with the additives.

After TPM normalization, the graphical presentation of results as radar plots in the published paper [16] shows only five of the 31 toxins increased in ingredient group 1 and 15 showed drops. The PM scientists concluded, on the basis of this analysis (and similar analyses for the other two ingredient groups), “that the addition of these 333 commonly used ingredients to cigarettes in three groups did not add to the toxicity of smoke, even at the exaggerated levels tested in the present series of studies” [16].

**Figure 2:**
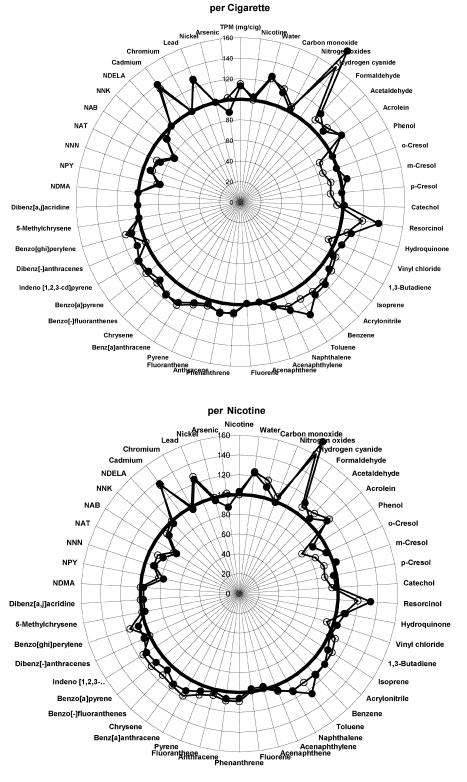
Graphical display of smoke constituents per cigarette and per unit of nicotine compared to control cigarettes for ingredient group 1. Low levels of additives are open circles, high levels are solid circles. Points outside the circle at 100% indicate increased levels of smoke constituents; points inside indicate less.

Smokers do not, however, smoke cigarettes to titrate their TPM exposure. They smoke whole cigarettes, generally to obtain a certain amount of nicotine [98]–[100]. Therefore we used PM's published results [16] to prepare a corresponding set of radar plots that present the levels of toxins per cigarette (as a fraction of control) and the ratio of the levels of toxin per unit nicotine for the cigarettes with additives compared to the level of toxin per unit nicotine in the control cigarettes (Figures 2–4). Presenting the Project MIX results this way provides a much different picture than that in PM's paper: On a per cigarette basis, 31 of 51 chemicals increased in at least one of the three ingredient groups over control (with 17 decreased), and 37 increased (and nine decreased) on a per unit nicotine basis.

**Figure 3 pmed-1001145-g003:**
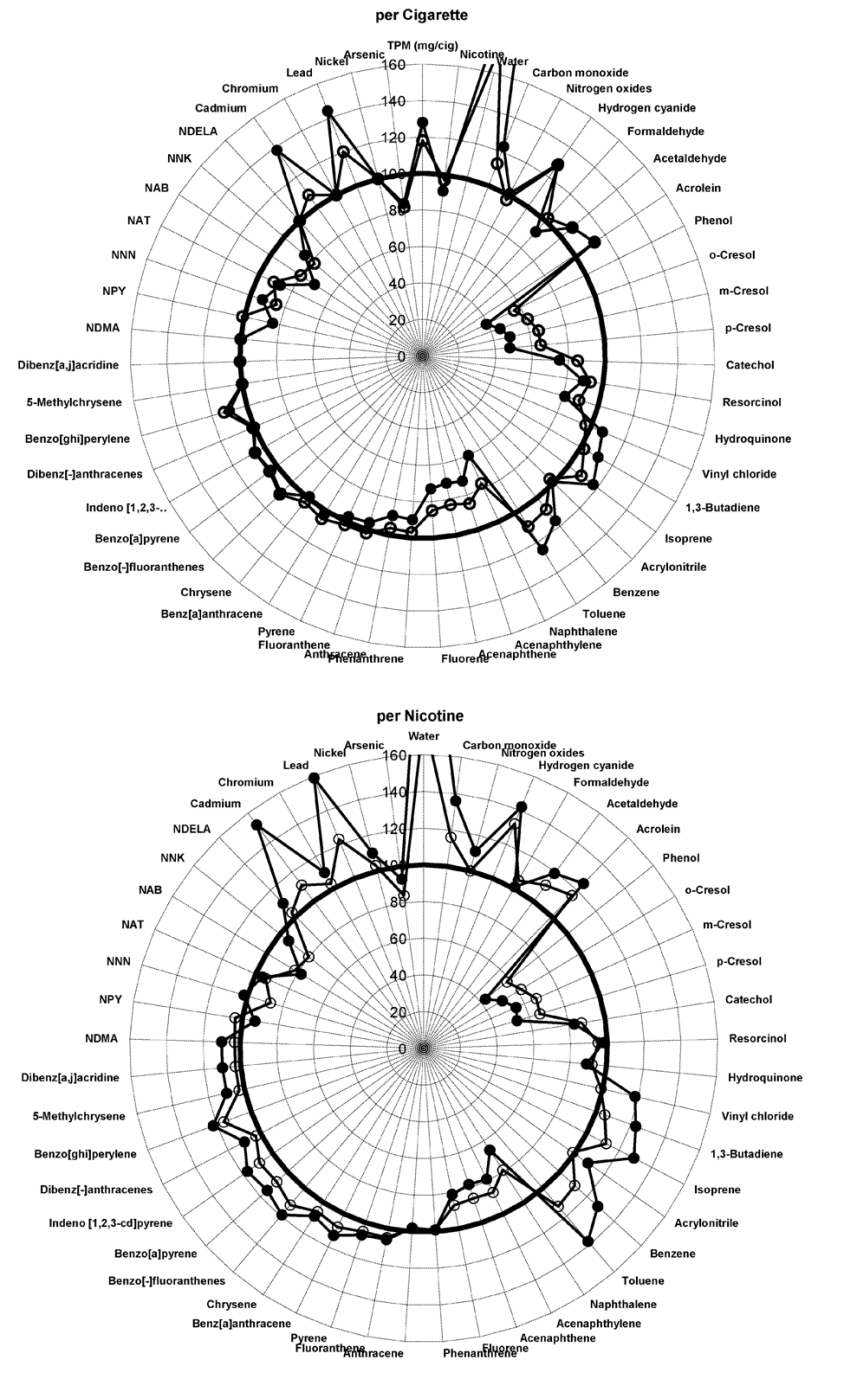
Graphical display of smoke constituents per cigarette and per unit of nicotine for ingredient group 2. Low levels of additives are open circles, high levels are solid circles. Points outside the circle at 100% indicate increased levels of smoke constituents; points inside indicate less.

**Figure 4:**
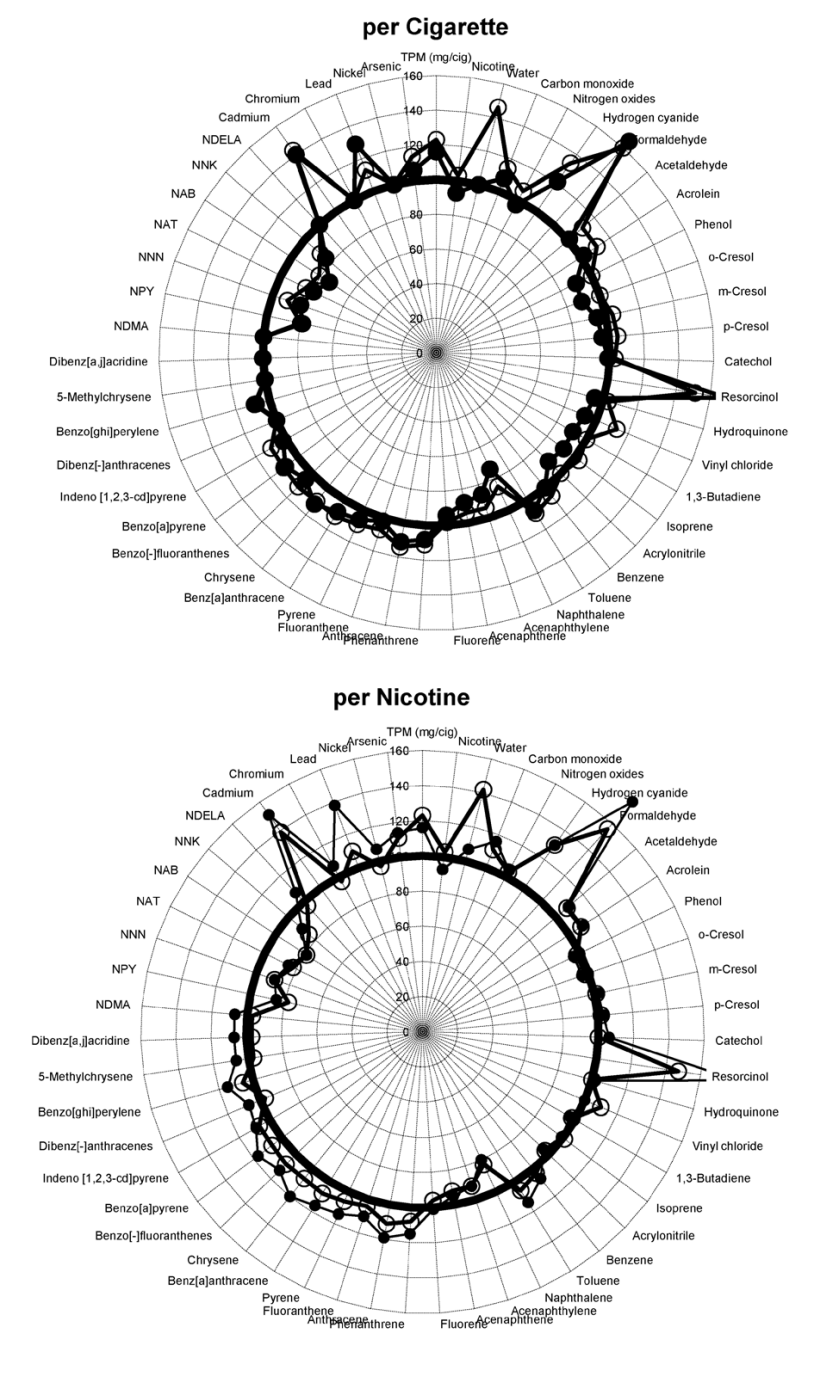
Graphical display of smoke constituents per cigarette and per unit of nicotine for ingredient group 3. Low levels of additives are open circles, high levels are solid circles. Points outside the circle at 100% indicate increased levels of smoke constituents; points inside indicate less.

Fifteen chemicals increased by 20% or more above the levels observed in the control cigarettes (Table 1). These chemicals include a number of human and animal carcinogens (arsenic, cadmium, 1,3-butadiene, lead, formaldehyde, and PAHs), respiratory irritants (e.g., acrolein), and cellular toxicants (hydrogen cyanide, carbon monoxide). The number of smoke constituents that increase by 20% or more rises from seven constituents when normalized by TPM to 18 when expressed per cigarette and 23 on a per nicotine basis. Likewise, 24 chemicals fell by more than a 20% reduction when normalized by TPM (all but nine are on the published radar plots), compared to 12 on a per cigarette basis and nine on a per nicotine basis (Figures 2–4).

**Table 1:** Chemicals that increased by more than 20% over control cigarette smoke.

Chemical	Per mg TPM (published radar plot [16])	Per Cigarette	Per mg Nicotine
***Ingredient group 1***			
Hydrogen cyanide	L H	L H	L H
Resorcinol	H	L H	L H
Toluene			H
Cadmium	L	L H	L H
Lead		L H	L H
Arsenic		H	
***Ingredient group 2***			
TPM		H	H
Carbon monoxide		H	H
Hydrogen cyanide		L H	L H
Acrolein			H
Resorcinol		L H	
1 3-Butadiene			H
Isoprene			H
Benzene			H
Toluene		H	H
Benzo[ghi]perylene			H
Cadmium		H	H
Lead		L H	L H
***Ingredient group 3***			
TPM		L	L
Hydrogen cyanide		L H	L H
Formaldehyde	L H	L H	L H
Resorcinol	L H	L H	L H
Chrysene			H
Cadmium	H	L H	L H
Lead		H	H

Empty cells indicate changes under 20%. Only chemicals that increased by 20% or more are listed.

L, low level of additives added (typical of actual usage in commercial cigarettes); H, high level (1.5–3 times the low level).

For unexplained reasons, the paper [16] excluded 19 of the 51 chemicals tested from the radar plots that formed the basis for discussing the results (1, Table S-2). Two of the excluded compounds significantly increased in smoke on a per cigarette basis of additive-added cigarettes, compared to smoke of control cigarettes, for all ingredient groups: benzo[-]fluoranthenes and benzo[ghi]perylene, both PAHs. Six more PAHs that were significantly higher in the smoke from cigarettes of ingredient groups 2 and 3 were also missing from the published radar plots—phenanthrene, anthracene, fluoranthene, pyrene, benz[a]anthracene, and chrysene—as well as a seventh, indeno[1,2,3-cd]pyrene, which was significantly increased in ingredient group 1. (We include all 51 smoke constituents in our analysis, shown in Figures 2–4 and Table 1.).

### In Vivo Subchronic Inhalation Testing

The in vivo animal toxicology portion of Project MIX consisted of exposing male and female Sprague-Dawley rats, nose only, to either fresh air (sham) or diluted mainstream smoke from the Project MIX control and test cigarettes for 90 d, followed by a 42-d postexposure period, after which time the animals were sacrificed and histopathology studies were completed [18]. The levels of smoke exposure were adjusted to expose all rats to the same level of TPM (150 µg TPM/l), regardless of which cigarettes (i.e., control or ingredient group) were used to generate the smoke.

The PM investigators [18] reported a “few minor differences” in the biological activity of the rats exposed to cigarettes with the additives, but dismissed these differences as “following no clear pattern,” suggesting they are “due to statistical inference.” The overall conclusion, reported in the paper's abstract is that “the data [from a 90-d inhalation study] indicate that the addition of the 333 commonly used ingredients, added to cigarettes in three groups, did not increase the inhalation toxicity of the smoke.”

The animal toxicology results [18] reported from Project MIX were based on a small number of rats in each experiment (usually nine), raising the possibility that the failure to detect statistically significant changes in the end points were due to underpowering the experiments rather than lack of a real effect. To explore this possibility, we assumed that the published descriptive statistics (means and standard deviations [SDs]) were unbiased estimates of the effects of those additives, then examined what the statistical conclusions would have been had Project MIX found these results on the basis of a sample size of 50 rats per group rather than nine rats per group. (While doing the actual experiment with a larger sample size would likely not yield precisely the same means and SDs as in the published paper, the differences should be randomly distributed on the assumption that the reported results are unbiased). We selected a sample size of 50 because PM used a much larger sample size of 99 rats (of each gender) in its study comparing the effects of secondhand smoke with diesel exhaust [101],[102]. When the reported standard error of the mean (SEM) was 0, we assumed the SD was 0.001. We then computed an analysis of variance using the reported means and SDs with an *n* of 50; if this ANOVA was significant (*p*<0.05), we then conducted multiple comparisons against control cigarettes with Holm-Sidak *t*-tests using a 0.05 family error rate. All calculations were done with Primer of Biostatistics (version 6) with the *n*, mean, SD option for ANOVA [103].

This exercise yielded 194 statistically significant changes (compared to the 26 reported in PM's published paper, Text S1,Table S-3), suggesting that a better powered study would have detected a much broader range of biological effects associated with the additives than identified in PM's published paper [18], suggesting that it substantially underestimates the toxic potential of cigarette smoke and additives.

## Discussion

The laboratory aspects of Project MIX studies appear to have been conducted using well-accepted laboratory practices, so the raw data are probably unbiased. The essential conclusion that the PM investigators reached, on the basis of these data, that “The studies with ingredients added to cigarettes did not demonstrate any meaningful effect of the ingredients on the toxicity of cigarettes” [15], however, is a reflection of the way that the data were normalized in the studies of toxins in the smoke [16] and low power of the animal toxicology studies [18]. Despite these problems, Project MIX results have been widely promoted to the scientific community [104]–[116], the public at large, and the Institute of Medicine [117], as well as cited by other tobacco industry scientists [118]–[122].

The conclusion that the additives did not increase the yield of toxins in the smoke is a direct result of the fact that the cigarettes containing the additives produced 15%–28% more TPM than the control tobacco-only cigarettes. The fact that the additives lead to more TPM is, itself, an important indicator of increased toxicity because the TPM in cigarette smoke leads to substantial increases in risk of cardiovascular disease, with a steep and highly nonlinear dose-response at low levels of exposure [123]–[126]. TPM exposure also disrupts physiological angiogenesis and contributes to ectopic pregnancy, spontaneous abortion, preterm delivery, sudden infant death syndrome, and slower wound healing [127].

In a letter to the editor of *Food and Chemical Toxicology*, Vleeming et al. [128] also commented on the fact that normalizing toxin production in smoke gave a misleading picture of the effects of the additives on smoke toxicity. They suggested that toxin levels should be normalized by the amount of tobacco in each cigarette. Since PM reduced the amount of tobacco when including the additives to maintain the mass of the cigarette constant, presenting the results normalized by tobacco weight led to even greater increases in the estimated effects of the toxins than we present (in our Figures 2–4). The PM researchers justified their use of TPM normalization on the grounds that “We choose TPM as our basis of comparison to be consistent with the animal and in vitro studies presented in this series of publications” and because “Since consumers choose to smoke cigarettes according to the tar delivery and taste, we chose to normalize the data to the TPM yield to reveal the effect of ingredients which contribute to the taste” [129]. The first of these arguments is surprising since, as noted above, the original design of Project MIX did not anticipate reporting and analyzing results normalized by TPM [130] and only introduced this normalization after PM had results showing that the additives led to higher levels of TPM (even though there was correspondingly less tobacco in the cigarettes). Also, as noted above, it is well-established that smokers smoke to control the delivery of nicotine, not tar (TPM) [98]–[100] and normalizing toxin deliveries by nicotine delivery yields higher toxicity estimates than normalizing by TPM (Figures 2–4; Table 1).

The fact that the in vivo toxicology studies [18] were conducted at matched levels of TPM for the smoke from all cigarettes (to hold TPM constant) also meant, as PM scientist Reininghaus noted internally in 1998 [96], that the rats breathing the smoke from the cigarettes with the additives were exposed to lower levels of toxins in the smoke than if the exposures had been matched on another smoke variable, such as nicotine delivery. To the extent that the mass of tobacco in the cigarette determines nicotine delivery, Vleeming et al.'s [128] approach suggests that the underestimates of toxin exposures the rats received compared to human smokers could be substantial.

Another problem with the in vivo toxicology studies is that PM exposed the animals to fixed levels of TPM and the ratio of TPM to gas phase toxins changes with the different ingredient groups. If the production of gas phase toxins increases less than the increase in TPM, then exposing the rats to a fixed amount of TPM will reduce exposure to the gaseous constituents. For physiological endpoints affected by these gases the toxicity might appear to decrease on the basis of per TPM versus per cigarette exposures. Indeed, when PM instructed INBIFO to analyze the smoke chemistry data on a per TPM basis [94] they noted that such an adjustment was already “compensated for by dilution in the subchronic study” [96].

The way the animal toxicology studies were designed with the relatively short (90 d) exposure period and follow-up after the end of the exposure (42 d) also raises concerns. PM's comparison of secondhand smoke with diesel exhaust (also conducted at INBIFO) had 99 rats of each gender in each exposure group, exposed the rats for 24 mo (730 d) with a 6-mo (183 d) follow-up [101],[102]. Longer exposure and follow-up would have increased the power of Project MIX to detect toxic effects of the additives. Even with these downward biases due to experimental design, however, our results suggest that an adequately powered design would have revealed a large number of toxic effects on the rats.

PM conducted extensive research on individual additives; the industry documents contain a list of 170 projects [131] conducted between 1973 and 1995 that includes many single additive projects. These single additive projects include INBIFO Project Juice, in which citric acid (one of the 333 ingredients in the present study) was added in low (2.6%), medium (3.9%), and high (6.3%) quantities to cigarette filler [132]. The amounts of citric acid reported for Project MIX ingredient group 1 were low level = 44 ppm, and high level = 122 ppm. It is not clear how these amounts of citric acid compare across studies. Studies were also conducted with lactic acid (Project Milk [133]), propylene glycol [134], and cocoa [34]. These studies were also conducted using low ratios between groups, along the order of only a 2- to 3-fold difference.

Project MIX also included in vitro genotoxicity and cytotoxicity studies [17] using a standard test panel PM developed in 1996, including the Ames test [14],[135]–[137]. Not surprisingly, the results of these tests found that all of the cigarettes, whether or not they included additives, were genotoxic and mutagenic. These tests, however, are screening tests, not sensitive measures of dose-response [138]–[141]. Therefore they are not appropriate for quantifying changes in toxicity associated with the additives. Absent the unlikely situation that the additives would eliminate tobacco smoke's genotoxicity and mutagenicity, the failure to find increased toxicity associated with the additives does not support the conclusion that “in vitro mutagenicity and cytotoxicity of the cigarette smoke were not increased by the addition of the ingredients” [17].

Scientists at British American Tobacco (BAT) conducted a similar project that in 2004 was also published in *Food and Chemical Toxicology* [142]–[144]. The BAT studies assessed 482 additives in various mixtures “at or above their typical maximum levels used on cigarettes sold by BAT” through an analysis of 45 selected smoke constituents in mainstream smoke of test cigarettes; in vitro mutagenicity and cytotoxicity tests (Ames test, mammalian cell micronucleus test, and neutral red uptake test) and 90-d inhalation studies in male and female Sprague-Dawley rats (ten rats per group). Using comparable rates of exposure of rats to smoke (approximately 20% less than the PM study rats), the BAT scientists report similar deleterious histopathological findings as Project MIX: increased density of goblet cells in respiratory epithelium, increased hyperplasia/degeneration of the nasal cavity, and increased epiglottal metaplasia. Similar to the PM scientists, the BAT scientists downplayed the findings by concluding that they were inconsistent, indicating that the response of rats exposed to smoke was indistinguishable between test and control cigarettes.

The kind of manipulation of the presentation of scientific results demonstrated by the publication of the Project MIX results [15]–[18], is nothing new for the tobacco industry; industry researchers have a long history of doing so around a variety of issues related to secondhand smoke [145]–[149]. While the procedures to collect the data themselves appear sound, the way that the data were analyzed and interpreted is not. An important implication of the analysis we present is that the scientific community and regulatory authorities cannot take the conclusions in tobacco industry (or industry-funded) research or research published in industry-dominated journals such as *Food and Chemical Toxicology* at face value. Vigorous implementation of FCTC article 5.3, which seeks to protect the policy making process against tobacco industry interference and manipulation [150], underscores the need for such skepticism in considering research such as Project MIX (and the corresponding papers from BAT) at face value in the rulemaking process. It will be important for the US FDA, WHO, and regulatory agencies in other countries who are working to implement FCTC articles 9–11 to insist on receiving all drafts of the study protocol (taking particular care to not allow the tobacco companies to use lawyer involvement in the process as a way to avoid disclosure) together with the raw data to reduce the likelihood of the problems identified in this paper.

If one accepts PM's assertion that Project MIX evaluated additives in groups that “resemble typical commercial blended cigarettes” [15], the data PM collected could be used for policy making regarding the use of these 333 additives. That the Project MIX scientists examined cigarette additives in combination (rather than singly) allows for the possibility that the additives act either synergistically or antagonistically. Allowing for this synergism is important. A comparison of the estimated lung cancer effect of tobacco smoke produced by summing the individual effects of constituents of tobacco smoke produced an estimate of cancer risk much smaller than the observed epidemiological risk [114]. Probably this is because all the carcinogens and cardiac toxins in tobacco smoke have not been identified and because there are likely interactions between the different constituents in the complex mixture that cigarette smoke represents.

It is also important to emphasize that the definition of “harm” implicit in PM's Project MIX and BAT's similar experiments [142]–[144]—direct short-term toxic effects of additives—is just one dimension of the effects that additives have on disease. Additives also have an effect on product palatability and pharmacologic properties (Box 1) in ways that make cigarettes less harsh and more pleasant to the user [3],[151],[152], leading to higher initiation and lower cessation and, so, more tobacco use and tobacco-induced disease [4],[19]. Indeed, it was primarily on the basis of these population-level impacts that the FDA Tobacco Products Scientific Advisory Committee concluded that, “Removal of menthol cigarettes from the marketplace would benefit public health in the United States” [19].

### Limitations

As noted in the Methods, this study is based on the documents that PM made available as a result of litigation against the company. While we were able to find several drafts and detailed results of the Project MIX studies, that, for example, allowed us to determine that the protocol for analyzing the data was altered after the results indicating increased TPM were obtained, there are key missing pieces of information: why PM chose the additives that they did for study, as well as the levels and combinations and the reason for selecting the sample sizes in the toxicology studies. This situation represents a limitation of the available sources of information and highlights the importance of the FDA using its new authority to obtain all sources of data on additive testing and other features of product manufacturing and testing and to make them fully available for review.

### Conclusion

The analysis in this paper shows that many of the toxins in cigarette smoke increase substantially when additives are put in cigarettes, including the level of TPM, and that, assuming that the toxicological results from Project MIX represent unbiased estimates of the true biological effects, these data show many adverse biological consequences (and that the failure to reach statistical significance was the result of underpowered studies rather than lack of an effect). In particular, regulatory authorities, including the FDA and similar agencies elsewhere who are implementing FCTC articles 9–11, could use the Project MIX data to eliminate the use of these 333 additives (including menthol, which is the major component of ingredient group 3) in cigarettes.

Any tobacco company would, of course, remain free to submit an application to the FDA, or other regulatory agency, to reintroduce use of an additive if they could provide convincing data from adequately powered studies that the additive truly did not have any adverse health consequences.

## Supporting Information

Text S1 **Three tables.** Table S-1, combined list of CPSC, IARC, and Project MIX smoke constituents for analysis; Table S-2, 19 smoke constituents not represented in published radar plots; and Table S-3, statistically significant toxicological changes associated with smoke from cigarettes from the three additive groups with two different sample sizes.
(PDF)

Alternative Language Abstract S1 **German translation of the abstract by TK.**
(DOC)

Alternative Language Abstract S2 **Chinese translation of the abstract by Ting Ting Yao.**
(DOC)

Alternative Language Abstract S3 **French translation of the abstract by Martine Wagnac.**
(DOC)

Alternative Language Abstract S4 **Spanish translation of the abstract by Ernesto Sebrie.**
(DOC)

Alternative Language Abstract S5 **Finnish translation of the abstract by Heikki Hiilamo.**
(DOC)

Alternative Language Abstract S6 **Russian translation of the abstract by Lyudmila Popova.**
(DOC)

Alternative Language Text S1 **German translation of the article by TK.**
(PDF)

Alternative Language Text S2 **German translation of Text S1 by TK.**
(PDF)

## Abbreviations

BAT: British American Tobacco
CPSC: US Consumer Product Safety Commission
FCTC: Framework Convention on Tobacco Control
FDA: Food and Drug Administration
IARC: International Agency for Research on Cancer
INBIFO: Institut für Biologische Forschung
LTDL: Legacy Tobacco Documents Library
PAH: polycyclic aromatic hydrocarbon
PM: Philip Morris
SD: standard deviation
TPM: total particulate matter
