# Supplementary material for: The Toxic Effects of Cigarette Additives. Philip Morris' Project Mix Reconsidered: An Analysis of Documents Released through Litigation
Source: PLoS Med. 2011 Dec 20;8(12):e1001145. doi: 10.1371/journal.pmed.1001145 (PMC3243707; doi:10.1371/journal.pmed.1001145)
Supplement: Text S1 — Three tables. Table S-1, combined list of CPSC, IARC, and Project MIX smoke constituents for analysis; Table S-2, 19 smoke constituents not represented in published radar plots; and Table S-3, statistically significant toxicological changes associated with smoke from cigarettes from the three additive groups with two different sample sizes. (PDF) [file pmed.1001145.s001.pdf]

THE TOXIC EFFECTS OF CIGARETTE ADDITIVES:

PHILIP MORRIS' PROJECT MIX RECONSIDERED

Supplementary Text

Marcia S. Wertz, PhD RN

Thomas Kyriss, MD

Suman Paranjape, PhD, MPH

Stanton A. Glantz, PhD

**Table S-1. Combined List of CPSC, IARC and Project MIX Smoke Constituents for Analysis**		
1,1-dimethylhydrazine	Benzo[c]phenanthrene	Naphthalene†
1,3-Butadiene (µg/cig) *	Benzo]c]pyrene	NAT *
1,4-dimethylphenanthrene	Benzo[ghi]perylene†	NDBA
1-methylchrysene	Cadmium *	NDEA
1-methylphenanthrene	Captan	NDELA *
1-naphthylamine	carbazole	NDMA *
2,3-dimethylmaleic anhydride	Carbon monoxide *	NDPA
2-cresol	Catechol *	NEMA
2-methylchrysene	Cholesterol	Nickel *
2-methylfluoranthene	Chromium *	Nicotine *
2-naphthylamine	Chrysene *	Nitrogen oxides *
2-nitropropane	Coronene	N-Nitroso-N-methylethylamine
3- and 4-cresol	Coumarin	NNK *
3- and 4-methylcatechol	Croton aldehyde	NNN *
3-methylchrysene	DDT	N-phenyl-2-naphthylamine
3-methylfluoranthene	Dibenz [*a*,*h*] acridine	NPIP
3-methylpyridine	Dibenz [a,j] acridine *	NPY *
4-aminobiphenyl	Dibenz[-]anthracenes *	ortho-anisidine
4-methylchrysene	Dibenzo [a,e]pyrene	o-cresol†
5-Methylchrysene *	Dibenzo [a,h]pyrene	o-toluidine
6-methylchrysene	Dibenzo [a,i]pyrene	p-cresol†
7H-Dibenzo[c,g]carbazole	Dibenzo[a,e]fluoranthene	Perylene
Acenaphthene†	Endrin	pH (acidity)
Acenaphthylene†	Ethylene oxide	Phenanthrene *
Acetaldehyde *	Eugenol	Phenol *
Acetamide	Fluoranthene *	Propionaldehyde
Acetic Acid	Fluorene *	Propylene oxide
Acetone	Formaldehyde *	Pyrene *
Acrolein *	Formic Acid	Pyridine
Acrylonitrile *	Hydrazine	Reduct/oxidat potential
Ammonia‡	Hydrogen cyanide *	Resorcinol *
Aniline	Hydroquinone *	Selenium
Anthanthrene	Indeno [1,2,3-cd]pyrene *	Styrene
Anthracene *	Isoprene (µg/cig) *	Succinic anhydride
Arsenic *	Lead (ng/cig) *	Tar
Benz[a]acridine	Malathion	Toluene *
Benz[a]anthracene *	Maleic anhydride	TPM *
Benz[c]acridine	Maleic hydrazide	Triphenylene
Benzene *	m-cresol†	Urethane
Benzo[-] fluorene	Methanol	Vinyl chloride *
Benzo[-]fluoranthenes *	Methyl acrylate	Water *
Benzo[a]pyrene *	NAB *	y-butyrolactose
*Included in Project MIX and CSPC or IARC<br>†Included in Project MIX but not CSPS or IARC		

‡Included in Project MIX but not reported [1]

**Table S-2. 19 Smoke constituents not represented in published radar plots** [1]		
Acenaphthalene L* IG2 & IG3	Acenaphthene L IG2 & IG3	Fluorene L IG2
Phenanthrene H IG1 & IG3 L IG2	Anthracene H IG1 & IG3 L IG2	Fluoranthene H IG1 & IG3
Pyrene H IG1 & IG3	Benz[a]anthracene H IG1 & IG3	Chrysene H IG1 & IG3
Benzo[-]fluoranthenes H All groups	Indeno [1,2,3-cd]pyrene H IG1	Dibenz[-]anthracenes
Benzo[ghi]perylene H All groups	5-Methylchrysene	Dibenz[a,j]acridine
NDMA	NDELA	Chromium
Nickel		
* indicates change in additive-added smoke is statistically different from control-cigarette smoke on a per cigarette basis (L=lower than control, H=higher than control, IG=Ingredient Group) reported in Rustemeier et al.'s [1] published Table 2 (2002).		

Table S-3. Statistically Significant Toxicological Changes Associated with Smoke from Cigarettes from the Three Additive Groups with Two Different Sample Sizes (Only significant results are shown.)				
**Health Parameter**	**Published Statistics Based on Observed Data** [2] **(N=4-14 per group†)**		**Assuming Same Mean and SD but N=50 per group**	
	**Male Rats**	**Female Rats**	**Male Rats**	**Female Rats**
Carboxyhemoglobin proportion in blood (%)			Increased 1L<br>Decreased 2L 1H	Decreased all
Urinary Nicotine metabolites* (%) NCOT			Increased 1L<br>Decreased 1H	Increased 1L 2L 3H
**Serum Chemistry**				
Alkaline Phosphatase			Decreased 2L	Increased 2H
Aspartate aminotransferase (AST)				Increased 2H<br>Decreased 3H
Alanine aminotrasferase (ALT)			Decreased 1H 2H	Increased 2H<br>Decreased 3L 3H
γGlutamyltransferase (GGT)				Increased 1L 2L 2H 3L
Total Cholesterol	Increased 2L 3L 3H		Increased 1L 2L 2H 3L 3H	Increased 3L
Triglycerides				Increased 1L 2L 2H 3L
Glucose				Decreased 1H 2L 2H 3L
Urea	Decreased 3L 3H	Increased 1H 2L	Decreased all	Increased all
**Hematology**				
Erythrocyte count			Increased 2L 3L 3H	Increased 1H 2H 3L
Hematocrit			Increased 1H 2L 3L	Increased 1H 2H 3L
Hemoglobin			Increased 1H 2L 2H 3L 3H	Increased 2H 3L
Platelet count			Increased 3H	Increased 1L 2L 3L 3H
Leukocyte count			Increased 1L 2H	Increased 1L 2H
Neutrophil count				Decreased 3H
Lymphocyte count			Increased 1L 2H 3L 3H	Decreased 1L 2H
**Organ Weights**				
Thymus	Increased 1L 1H 2H 3L 3H	Increased 3H	Increased all	Increased 3H<br>Decreased 1L
Liver	Increased 2H		Increased 2H 3H	Increased 2L
Adrenals				Increased 1L 2L 2H
Lungs	Increased 2H		Increased 1H 2L	Increased 1L 1H 2L 3H
Kidneys				
**Histopathology in Nose**				
Nasal Cavity Level 1				
Resp. epithelium, reserve cell hyperplasia		Increased 1L	Decreased 1L 1H 2H	Increased 1L 1H 2L 2H 3L

Squamous metaplasia				Increased 3H<br>Decreased 2H
Goblet-cell hyperplasia				Increased 3H
Loss of goblet cells			Increased 1L 1H 2L	Decreased 2L 2H 3L 3H
Nasal Cavity Level 2				
Resp. epithelium, reserve cell hyperplasia				Increased 2H 3H
Olfactory epithelium atrophy	Decreased 2H	Increased 2L 2H	Decreased 1L 2H 3L	Increased 1L 1H 2L 2H 3L
Squamous metaplasia				Increased 2H
Nasal Cavity Level 3				
Olfactory epithelium atrophy		Increased 2L 2H	Decreased 2H 3L	Increased 1L 1H 2L 2H 3L
Squamous metaplasia		Increased 2H	Decreased 2H	Increased 1L 1H 2L 2H 3L
Nasal cavity Level 4				
Olfactory epithelium atrophy				Increased 1L 2L
Squamous metaplasia			Decreased 2H	Increased 1L 2H
**Histopathology of Larynx**				
Base of Epiglottis				
Pseudostratified epithelium squamous metaplasia			Increased 2L 2H	Decreased 1H 3L 3H
Squamous epithelium hyperplasia			Increased 1H 2L 2H 3L	Increased 1H
Floor of larynx (arythenoid projections)				
Pseudostratified epithelium squamous metaplasia		Decreased 3H		Decreased All
Vocal cords, lower medial				
Squamous epithelium hyperplasia			Decreased 1L 3H	Decreased 1H 3L 3H
Vocal cords, upper medial				
Pseudostratified epithelium squamous metaplasia				
Vocal folds				
Squamous epithelium hyperplasia				Decreased 1H 2L 2H
**Thickness of laryngeal epithelium**				
Floor of the Lanryx	Increased 3L	Increased 3L 3H	Increased 2L 2H 3L	Decreased 1H 3L 3H
**Histopathology of the Trachea, Bronchi and Lungs**				
Tracheal Bifurcation				
Resp. epithelium goblet cell			Increased 2L	Decreased 3H

hyperplasia				
Right Lung				
Alveolar macrophages			Increased 3H	Increased 3H<br>Decreased 2H
Left Lung				
Resp. epithelium goblet cell hyperplasia			Increased 2H	Decreased 3H
Alveolar macrophages			Increased 3H<br>Decreased 2H	

†N varied (e.g.4-5 rats per group for HbCO%, 8 rats per group for nicotine metabolites, 8-10 rats per group for chemistry, hematology, and pathology exams except14 rats in all control groups).
Number = Ingredient Group (e.g. 2=Ingredient Group 2); L or H refers to low or high level of additives in Ingredient Group.
Only statistically significant differences reported by the PM investigators[2] (first two columns, using analytical methods described in that paper) and the authors' reanalysis (last two columns) assuming the same means and standard deviation as PM reported, but increasing the sample size to 50 rats in each group. An empty box indicates no significant statistical difference.
*Cigarettes with additives are reported to have less tobacco (substituted by the additives), and therefore less nicotine, which may account for the decrease in cotinine levels when there was one.

REFRENCES

1. Rustemeier K, Stabbert R, Haussmann HJ, Roemer E, Carmines EL (2002) Evaluation of the potential effects of ingredients added to cigarettes. Part 2: chemical composition of mainstream smoke. Food Chem Toxicol 40: 93-104.
2. Vanscheeuwijck PM, Teredesai A, Terpstra PM, Verbeeck J, Kuhl P, et al. (2002) Evaluation of the potential effects of ingredients added to cigarettes. Part 4: subchronic inhalation toxicity. Food Chem Toxicol 40: 113-131.
