# Supplementary material for: The Toxic Effects of Cigarette Additives. Philip Morris' Project Mix Reconsidered: An Analysis of Documents Released through Litigation
Source: PLoS Med. 2011 Dec 20;8(12):e1001145. doi: 10.1371/journal.pmed.1001145 (PMC3243707; doi:10.1371/journal.pmed.1001145)
Supplement: Alternative Language Abstract S1 — German translation of the abstract by TK. (DOC) [file pmed.1001145.s002.doc]

**DIE TOXISCHE WIRKUNG VON ZIGARETTENADDITIVEN:**

**PHILIP MORRIS’ PROJECT MIX AUF DEM PRÜFSTAND**

**Fragestellung:** Inwieweit kann das Philip Morris’ Projekt MIX als Fallstudie für eine strategisch ausgerichtete wissenschaftliche Forschungsarbeit der Tabakindustrie gelten, die absehbaren Anti-Tabakmaßnahmen entgegengestellt wurde?

**Hintergrund:** Ein Erlass der US-Food and Drug Administration (FDA) zielte 2009 auf Additive in Zigaretten. Die Tabakindustrie bereitete sich auf diesen Fall vor, indem sie ein Forschungsprogramm zur Toxizität von Additiven startete.

**Methode und Ergebnisse:** Vormals geheime Tabakindustriedokumente wurden ausgewertet, um industrieinterne Forschungsvorhaben zu Zigarettenadditiven mit den darüber veröffentlichten Ergebnissen aus der Fachliteratur zu vergleichen. Wir konzentrierten uns dabei auf eine Forschungsreihe, die Philip Morris in einem koordinierten Ansatz durchführte und die firmenintern als „Projekt MIX“ bezeichnet wurde. Die internen Industriedokumente zeigten, dass unter Projekt MIX verschiedene Kombinationen von 333 Zigarettenadditiven untersucht wurden. Neben zahlreichen internen Berichten entstammen diesem Projekt auch 4 Veröffentlichungen in der Fachliteratur. Diese Arbeiten schließen, dass die untersuchten Zusatzstoffe nicht wesentlich zur Toxizität beitragen würden. Die internen Industriedokumente enthüllen nachträgliche Veränderungen in den Analyseprotokollen, die vorgenommen wurden, nachdem statistische Berechnungen eine mit Additiven assoziierte Zunahme der Zigarettentoxizität und des Rohkondensats im Zigarettenrauch ergaben. In den Veröffentlichungen wurden der Anstieg der vorhandenen Toxizität und die Partikelmenge verschleiert, indem diese bezogen auf das Rohkondensat dargestellt wurden.

**Schlussfolgerung:** Diese Fallstudie über das Projekt MIX macht klar, dass Forschungsergebnisse der Tabakindustrie über Zigarettenadditive genauestens geprüft werden müssen. Die Ergebnisse zeigen, dass Rohkondensat und Toxine im Zigarettenrauch beträchtlich zunehmen, wenn Additive verwendet werden. Insbesondere Zulassungsbehörden, wie die FDA und ähnliche Einrichtungen andernorts, könnten Daten aus Projekt MIX verwenden, um den Einsatz dieser 333 Zusatzstoffe (einschließlich Menthol) zu unterbinden.
