# Supplementary material for: The Toxic Effects of Cigarette Additives. Philip Morris' Project Mix Reconsidered: An Analysis of Documents Released through Litigation
Source: PLoS Med. 2011 Dec 20;8(12):e1001145. doi: 10.1371/journal.pmed.1001145 (PMC3243707; doi:10.1371/journal.pmed.1001145)
Supplement: Alternative Language Abstract S2 — Chinese translation of the abstract by Ting Ting Yao. (DOC) [file pmed.1001145.s003.doc]

**香烟添加剂的毒性作用:**

**对菲利普莫里斯“混合项目”的重新思考**

**摘要**

**研究目的:**以菲利普莫里斯烟草公司的“混合项目”为案例，分析烟草企业有针对性地开展科研以反对控烟政策的行为。

**研究背景:** 2009年，美国食品药品管理局颁布了有关香烟味道添加剂的规则。而烟草企业早就为此作足了准备—即启动了一项有关添加剂毒性的研究计划。

**研究方法和结果:** 我们通过分析烟草企业以前的秘密文件以确定其内部有关香烟味道添加剂的研究战略，并重新分析了烟草业曾发表的研究结果。我们的研究重点为一系列菲利普莫里斯开展的被称为“混合项目”的研究。文件分析显示“混合项目”包括了对333种香烟味道添加剂进行各种组合的研究。除了形成多份内部报告之外，烟草企业的这项研究还发表了四篇同行评审的文章（发表于2001年）。这些发表的文章均指出没有证据显示香烟味道添加剂中含有过剂量的毒性。我们对内部文件的分析显示：初步统计结果显示了与添加剂有关的香烟毒性的增加以及香烟烟雾中总颗粒物浓度的增加。而烟草企业却通过调整总颗粒物浓度的数据，使得发表的文章淡化了香烟味道添加剂潜在的毒性和增加的颗粒物浓度。

**总结:**该案例研究显示烟草企业有关香烟添加剂的研究结果不能被采用。我们的研究结果表明当在香烟中使用味道添加剂时，香烟烟雾中（包括总颗粒物）的毒性会大大增加。我们建议监管机构（包括食品药品管理局和其他相似的机构）可以使用“混合项目”的数据来禁止香烟中对这333项味道添加剂（包括薄荷味）的使用。
