# Supplementary material for: The Toxic Effects of Cigarette Additives. Philip Morris' Project Mix Reconsidered: An Analysis of Documents Released through Litigation
Source: PLoS Med. 2011 Dec 20;8(12):e1001145. doi: 10.1371/journal.pmed.1001145 (PMC3243707; doi:10.1371/journal.pmed.1001145)
Supplement: Alternative Language Abstract S3 — French translation of the abstract by Martine Wagnac. (DOC) [file pmed.1001145.s004.doc]

LES EFFETS TOXIQUES DES ADDITIFS DANS LES CIGARETTES

ÉTUDE DU PROJET MIX DE PHILIP MORRIS

Marcia S. Wertz, PhD, RN^1,2^

Thomas Kyriss, MD^3^

Suman Paranjape, PhD, MPH^1^

Stanton A. Glantz, PhD^1,4^

^1^Centre pour la Recherche et de l’éducation du Control du Tabac, Université de Californie, San Francisco.

^2^Département des Sciences Sociales et du Somportement, école des Sciences Infirmières, Université de Californie, San Francisco ([marcia.wertz@ucsf.edu](mailto:marcia.wertz@ucsf.edu))

^3^Hôpital Schillerhoehe, Chirurgie Thoracique, Solitudestrasse 18, D-70893 Gerlingen, Allemagne ([kyriss@klinik-schillerhoehe.de](mailto:kyriss@klinik-schillerhoehe.de))

^4^Département de Médecine, Université de Californie, San Francisco ([glantz@medicine.ucsf.edu](mailto:glantz@medicine.ucsf.edu))

**Objectif:**

Analyser le Projet MIX de la compagnie Philip Morris en tant qu’étude de cas sur les méthodes utilisées par l’industrie du tabac pour positionner leurs recherches scientifiques en vue de s’opposer aux politiques anticipés sur le contrôle des produits du tabac.

**Historique:**

En 2009, la « Food and Drug Administration  (FDA) » a promulgué des règlements qui portaient sur des additifs ajoutés aux cigarettes. L’industrie du tabac s’était préparée à cette éventualité en établissant un programme de recherche qui se concentrait sur la toxicité des saveurs ajoutées.

**Méthode et Résultat:**

Nous avons analysé des documents de l’industrie du tabac, qui jusqu'à tout récemment étaient classifiés secrets, pour identifier les stratégies internes utilisées dans la direction des  recherches sur les saveurs incorporées aux cigarettes.   Nous avons de nouveau fait l’étude des articles publiées par l’industrie du tabac elle-même et qui ont été soumis  à la revue arbitrée.  Nous nous sommes concentrés sur le groupe clé de l’étude réalisée par Philip Morris dans un effort coordonné appelé « Projet Mix ».  Les documents démontrent que le Projet Mix comprend l’étude de plusieurs arrangements de 333 additifs de saveurs de cigarettes.  De multiples rapports internes ont découlé de cette étude ainsi que quatre articles qui ont été revus par les pairs de l’industrie du tabac avant leur publication (en 2001). Ces études ont conclues qu’il n’y avait pas d’évidence de la toxicité substantive attribuable aux saveurs ajoutées étudiées.  Des documents internes ont démontré des changements (post-hoc) dans le protocole analytique après que les premiers résultats statistiques ont indiqué une augmentation de la toxicité des cigarettes associée aux saveurs ajoutées, ainsi qu’une augmentation de la concentration de « Total Particulate Matter  (TPM) » dans la fumée des cigarettes ayant été additionnées de saveurs.   En utilisant les données qui ont manipulées la concentration de TPM, les articles publiés ont dissimulé cette présente toxicité ainsi que l’augmentation des particules.

**Conclusion:**

L’étude de cas du projet Mix démontre que les recherches scientifiques des compagnies de tabac sur l’utilisation de saveurs ajoutées des cigarettes ne peuvent être considérées véridiques. Les résultats ont démontré que les toxines présentes dans la fumée de cigarette augmentent de façon substantielle lorsque les saveurs sont ajoutées aux cigarettes, y compris le niveau de TPM.  De même, les autorités sanitaires incluant la FDA et d’autres agences similaires pourraient utiliser les données du Projet Mix pour interdire l’utilisation de ces 333 produits  (y compris le menthol).
