# Supplementary material for: The Toxic Effects of Cigarette Additives. Philip Morris' Project Mix Reconsidered: An Analysis of Documents Released through Litigation
Source: PLoS Med. 2011 Dec 20;8(12):e1001145. doi: 10.1371/journal.pmed.1001145 (PMC3243707; doi:10.1371/journal.pmed.1001145)
Supplement: Alternative Language Abstract S4 — Spanish translation of the abstract by Ernesto Sebrie. (DOC) [file pmed.1001145.s005.doc]

**LOS EFECTOS TOXICOS DE LOS ADITIVOS DE CIGARRILLOS:**

**EL PROYECTO MIX DE PHILIP MORRIS RECONSIDERADO**

**RESUMEN**

**Objetivo:** Analizar el Proyecto MIX de Philip Morris como un caso de estudio de investigación científica de la industria del tabaco posicionada estratégicamente para oponerse a políticas anticipadas de control del tabaco.

**Antecedentes:** En 2009, la promulgación de la regulación del tabaco de la Administración de Medicamentos y Alimentos (FDA por sus siglas en inglés) enfocó la atención sobre los aditivos de sabor en los cigarrillos. La industria del tabaco se había preparado para esta eventualidad iniciando un programa de investigación enfocado en la toxicidad de los aditivos.

**Métodos y Resultados: A**nalizamos documentos previamente secretos de la industria del tabaco para identificar estrategias internas de investigación sobre aditivos en cigarrillos y reanalizamos resultados de investigaciones de la industria tabacalera publicados en artículos revisados por pares. Nos centramos en el grupo clave de estudios conducido por Phillip Morris en un esfuerzo coordinado conocido como “Proyecto MIX.” Los documentos mostraron que el Proyecto MIX incluyó el estudio de varias combinaciones de 333 aditivos de cigarrillos. Además de múltiples reportes internos, este estudio resultó en la publicación de cuatro artículos revisados por pares (publicados en 2001). Estos artículos concluyeron que no había evidencia de toxicidad sustantiva atribuible a los aditivos de cigarrillos estudiados. Documentos internos revelaron cambios post-hoc en los protocolos analíticos después que resultados estadísticos iniciales indicaron un incremento en la toxicidad asociada al aditivo del cigarrillo así como también un aumento en las concentraciones de materia particulada total (TPM por sus siglas en inglés), en el humo de cigarrillo modificado por aditivo. Al presentar los datos ajustados por la concentración de TPM, los artículos publicados ocultaron esta toxicidad subyacente y el incremento en las partículas.

**Conclusión:** El estudio de caso del Proyecto MIX muestra que la investigación científica de la industria tabacalera sobre el uso de aditivos en cigarrillos no puede ser tomada en serio. Los resultados demuestran que las toxinas del humo de cigarrillo aumentan sustancialmente cuando los aditivos son agregados a los cigarrillos, incluyendo el nivel de TPM. En particular, las autoridades de regulación, incluyendo la FDA y agencias similares de otros lugares podrían usar los datos del Proyecto Mix para eliminar el uso de estos 333 aditivos (incluyendo el mentol) de los cigarrillos.
