# Supplementary material for: The Toxic Effects of Cigarette Additives. Philip Morris' Project Mix Reconsidered: An Analysis of Documents Released through Litigation
Source: PLoS Med. 2011 Dec 20;8(12):e1001145. doi: 10.1371/journal.pmed.1001145 (PMC3243707; doi:10.1371/journal.pmed.1001145)
Supplement: Alternative Language Abstract S5 — Finnish translation of the abstract by Heikki Hiilamo. (DOC) [file pmed.1001145.s006.doc]

**Savukkeiden lisäaineiden haitallisuus: Philip Morrisin MIX-hanke uudelleentarkastelussa**

**Tavoite:** Analysoida Philip Morrisin MIX hanketta tapaustutkimuksena tupakkateollisuuden tieteellisestä tutkimuksesta, jonka strategisena tavoitteena on tupakkasääntelyn estäminen.

**Tausta:** Yhdysvaltain elintarvike- ja lääkeviraston (Food and Drug Administratio, FDA) vuonna 2009 saama valtuutus säädellä tupakkaa nosti esille savukkeiden makua tuottavat lisäaineet. Tupakkateollisuus oli valmistautunut tähän käynnistämällä tutkimusohjelman lisäaineiden haitallisuudesta.

**Metodit ja tulokset:** Analysoimme aikaisemmin salaisia tupakkateollisuuden sisäisiä asiakirjoja ja yritimme paikallistaa tupakkayhtiöiden omia strategioita savukkeiden lisäaineiden tutkimuksessa. Analysoimme näiden tietojen valossa uudelleen tupakkateollisuuden vertaisarvioiduissa tutkimuksissa julkaistuja tuloksia. Keskityimme yhteen avain hankkeeseen eli Philip Morrisin MIX-hankkeeseen. Asiakirjat osoittivat, että MIX -hankkeessa tutkittiin yhteensä 333 eri lisäaineen kombinaatioita. Lukuisten sisäisten raporttien lisäksi hanke johti neljään vuonna 2001 ilmestyneeseen vertaisarvioituun artikkeliin. Tutkimusten johtopäätöksenä oli, etteivät lisäaineet olleet merkittävissä määrin haitallisia. Sisäiset asiakirjat paljastivat, että analyysitapoja oli muutettu sen jälkeen, kun ensimmäiset tilastolliset ajot olivat osoittaneet lisäaineisiin liittyvää savukkeiden haitallisuuden lisääntymistä sekä pienhiukkasten määrän lisääntymistä lisäaineita sisältävien savukkeiden savussa. Koska julkaistuissa tutkimuksissa tulokset suhteutettiin pienhiukkasten tiheyteen, tulosten kuva haitallisuuden ja pienhiukkasten määrän lisääntymisestä hämärtyi.

**Johtopäätökset:** Tapaustutkimus MIX-hankkeesta osoittaa, ettei tupakkateollisuuden tutkimustuloksia savukkeiden lisäaineista voida pitää luotettavina. Tulokset osoittavat, että savukkeiden haitallisuus (ml. pienhiukkasten määrä) lisääntyy selvästi lisäaineiden vuoksi. Valvovat viranomaiset kuten FDA voivat käyttää MIX -hankkeen tilastoaineistoa näiden 333 lisäaineen (mukaan lukien mentoli) poistamiseksi savukkeista.
