# Supplementary material for: The Toxic Effects of Cigarette Additives. Philip Morris' Project Mix Reconsidered: An Analysis of Documents Released through Litigation
Source: PLoS Med. 2011 Dec 20;8(12):e1001145. doi: 10.1371/journal.pmed.1001145 (PMC3243707; doi:10.1371/journal.pmed.1001145)
Supplement: Alternative Language Abstract S6 — Russian translation of the abstract by Lyudmila Popova. (DOC) [file pmed.1001145.s007.doc]

**Токсичный эффект добавок в сигареты: Новый взгляд на Проект «МИКС» компании Филип Моррис**

**Цель**: Ситуационный анализ Проекта «МИКС» компании Филип Моррис как образца научных исследований проводимых табачной индустрией в подготовке к ожидаемой политике контроля над табаком.

**Состояние Вопроса:** Обнародование в 2009 г. Управлением по Контролю за Качеством Пищевых Продуктов и Лекарственных Препаратов (FDA) табачного урегулирования заострило внимание на вкусовых добавках в сигареты. Табачная индустрия была готова к такому повороту событий, заранее инициировав исследовательскую программу по токсичности добавок.

**Методы и Результаты:** Мы проанализировали прежде секретные документы табачной индустрии с целью определить внутреннюю стратегию исследований по добавкам в сигареты, а также повторно проанализировали результаты этих исследований, опубликованные в рецензируемых изданиях. Мы сосредоточили наше внимание на ключевой группе исследований, проведенных компанией Филип Моррис под кодовым названием Проект «МИКС». Документы показали, что Проект «МИКС» включал в себя исследования различных комбинаций из 333 сигаретных добавок. Результаты этого проекта можно найти в многочисленных внутренних отчетах и помимо этого, в четырех публикациях в рецензируемых изданиях (2001 г.). В этих публикациях заявлялось, что исследуемые сигаретные добавки не являются токсичными. Внутренние документы выявили последующие поправки к аналитическому протоколу, после того как изначальные статистические выводы показали повышение сигаретной токсичности, связанное с добавками, а также повышение общего содержания твердых частиц в дыме сигарет с добавками. В опубликованных работах результаты были представлены с поправкой на концентрацию твердых частиц, скрыв таким образом настоящие уровни токсичности и повышенной концентрации частиц.

**Заключение:** Анализ Проекта «МИКС» компании Филип Моррис показывает, что нельзя слепо доверять результатам научных исследований о добавках в сигареты, проводимых табачной индустрией. Результаты демонстрируют, что содержание токсинов и тяжелых частиц в сигаретном дыме значительно повысилось после включения в них добавок. В частности, управляющие органы, такие как FDA и подобные агенства, могут использовать результаты Проекта «МИКС» для устранения использования этих 333 добавок (включая ментол) в сигаретах.
