# Supplementary material for: The Toxic Effects of Cigarette Additives. Philip Morris' Project Mix Reconsidered: An Analysis of Documents Released through Litigation
Source: PLoS Med. 2011 Dec 20;8(12):e1001145. doi: 10.1371/journal.pmed.1001145 (PMC3243707; doi:10.1371/journal.pmed.1001145)
Supplement: Alternative Language Text S1 — German translation of the article by TK. (PDF) [file pmed.1001145.s008.pdf]

Die toxische Wirkung von Zigarettenadditiven. Das Philip Morris Projekt Mix auf dem Prüfstand: eine Analyse von Dokumenten, die als Folge von Haftungsklagen herausgegeben wurden

Marcia S. Wertz[1,2], Thomas Kyriss[3], Suman Paranjape[1], Stanton A. Glantz[1,4*]

1 Center for Tobacco Control Research and Education, University of California San Francisco, San Francisco, California, United States of America,
2 Department of Social and Behavioral Sciences, School of Nursing, University of California San Francisco San Francisco, California, United States of America,
3 Thoracic Surgery, Schillerhoehe Hospital, Gerlingen, Germany,
4 Department of Medicine, University of California San Francisco, San Francisco, California, United States of America

* E-mail: glantz@medicine.ucsf.edu

Deutsche Übersetzung von: Wertz MS, Kyriss T, Paranjape S, Glantz SA (2011) The Toxic Effects of Cigarette Additives. Philip Morris' Project Mix Reconsidered: An Analysis of Documents Released Through Litigation. PLoS Med 8(12): e1001145. doi:10.1371/journal.pmed.1001145 (übersetzt von Thomas Kyriss)

ABSTRAKT
Hintergrund: Im Jahr 2009 richtete sich ein Erlass der US Food and Drug Administration (FDA) gegen Additive in Zigaretten. Die Tabakindustrie bereitete sich auf diesen Fall vor, indem sie ein Forschungsprogramm zur Toxizität von Additiven veranlasste. Die Fragestellung der vorliegenden Studie war, inwieweit das Philip Morris' Projekt MIX als Fallstudie für eine strategisch ausgerichtete wissenschaftliche Forschungsarbeit der Tabakindustrie gelten kann, um absehbare Tabakkontrollverordnungen abzuwenden.
Methode und Ergebnisse: Vormals geheime Tabakindustriedokumente wurden ausgewertet, um industrieinterne Forschungsvorhaben zu Zigarettenadditiven mit den darüber veröffentlichten Ergebnissen aus der Fachliteratur zu vergleichen. Wir konzentrierten uns dabei auf eine Forschungsreihe, die Philip Morris in einem koordinierten Ansatz durchführte und die firmenintern als „Projekt MIX" bezeichnet wurde. Die internen Industriedokumente zeigten, dass unter Projekt MIX verschiedene Kombinationen von 333 Zigarettenadditiven untersucht wurden. Neben zahlreichen internen Berichten entstammen diesem Projekt auch 4 Veröffentlichungen in der Fachliteratur. Diese Arbeiten schließen, dass die untersuchten Zusatzstoffe nicht wesentlich zur Toxizität beitragen würden. Die internen Industriedokumente enthüllen nachträgliche Veränderungen in den Analyseprotokollen, die vorgenommen wurden, nachdem statistische Berechnungen eine mit Additiven assoziierte Zunahme der Zigarettentoxizität und des Rohkondensats im Zigarettenrauch ergaben. In den Veröffentlichungen wurden der Anstieg der vorhandenen Toxizität und die Partikelmenge verschleiert, indem diese bezogen auf das Rohkondensat dargestellt wurden. Die Ergebnisse der tiertoxikologischen Untersuchungen basierten in den einzelnen Experimenten auf einer kleinen Anzahl von Ratten, was die Frage aufwirft, ob die Ergebnisse unterhalb des Signifikanzniveaus eher mangelnder Teststärke als nicht vorhandener Wirkung zuzuschreiben sind.
Schlussfolgerung: Diese Fallstudie über das Projekt MIX macht klar, dass Forschungsergebnisse der Tabakindustrie über Zigarettenadditive genauestens geprüft werden müssen. Die Ergebnisse zeigen, dass Rohkondensat und Toxine im Zigarettenrauch beträchtlich zunehmen, wenn Additive verwendet werden. Insbesondere Zulassungsbehörden, wie die FDA und vergleichbare Einrichtungen andernorts, könnten die Daten aus Projekt MIX verwenden, um den Einsatz dieser 333 Zusatzstoffe (einschließlich Menthol) zu unterbinden.

Die Aufbereitung der Zusammenfassung dieses Artikels erfolgte entsprechend der Referenzen.

EINLEITUNG

Spätestens seit dem Jahr 1963 hat sich die Tabakindustrie auf gesetzliche Regulierungen ihrer Produkte eingestellt. Damals zeigte sich der Philip Morris (PM) Vizepräsident der Forschungs- und Entwicklungsabteilung Helmut Wakeham besorgt, dass das bestehende Feststellungs-verfahren der US-Kontrollbehörde zur Lebens- und Arzneimittel-Überwachung (US Food and Drug Administration, FDA) für unbedenkliche Nahrungsmittel und -inhaltsstoffe, („GRAS, Generally Recognized As Safe"), für Lebensmittelzusatzstoffe, aber nicht für Zigaretten gelten würde und stellte fest: "Streng genommen könnten nur publizierte Studien herangezogen werden, um eine GRAS-Liste für inhalierte Substanzen aufzustellen. Dazu gibt es relativ wenige Studien. Diese Ausgangslage würde im Falle von Zigaretten hervorragenden Schutz bieten" [Übersetzung der Autoren, ÜdA] [1]. 1984 wurde in den USA die Sicherheit von Zigarettenadditiven zum ersten Mal zu einer öffentlichen Angelegenheit [2]. Zusatzstoffe sind bedeutende Bestandteile von Tabakprodukten, weil sie den Herstellern ermöglichen, die sensorischen und pharmakologischen Eigenschaften von Tabakprodukten zu beeinflussen. Der Einstieg zum Rauchbeginn und die Entwöhnung vom Rauchen werden durch Additive beeinflusst, weil diese den Rauch milder oder angenehmer für den Raucher machen oder die Bioverfügbarkeit und die Wirkung von Nikotin beeinflussen, wie dies durch die Veränderungen des pH-Wertes im Rauch oder durch Additive wie Menthol geschieht [3,4] (Kasten 1). Gesetzliche Bestimmungen zum Gebrauch von Additiven, bis hin zu einem Verbot, stellen eine ernste Bedrohung für die Tabakhersteller dar. Die Angst vor Regulierung nahm zu, als Präsident William Clinton 1995 der FDA ermöglichte, Verordnungen Zigaretten betreffend zu erlassen. Während die Tabakindustrie in einer Entscheidung vor dem Obersten (US) Gerichtshof noch erreichte, dass die FDA nicht die Befugnis hat gesetzliche Bestimmungen zu Tabakprodukten zu erlassen, stellte die PM Geschäftsführung Mitte der 1990er Jahre fest, dass gesetzliche Bestimmungen unausweichlich wären. Die Tabakindustrie versuchte die Rechtsvorschriften des "Family Smoking Prevention and Tobacco Control Act" [5] zu beeinflussen, der 2009 die FDA zur zuständigen Behörde für Tabakprodukte machte [6,7,8]. (Zu den ersten Maß-nahmen des neu gegründeten Zentrums für Tabakprodukte der FDA gehörte 2009 das Verbot von Geschmacksstoffen in Zigaretten, mit Ausnahme von Tabak und Menthol [9].) Während über die US Gesetzgebung zur Regulierung von Tabakprodukten beraten wurde, entwickelten verschiedene Tabakkonzerne Verfahren, um die biologische Wirkung neuer Produkte zu bestimmen, die Nikotin anders als herkömmliche Zigaretten abgeben [10]. Ein weltweites Thema wurde die Regulierung von Tabakerzeugnissen mit der Entwicklung und Umsetzung des WHO Rahmenabkommens zur Tabakkontrolle (FCTC) [11]. Die FCTC Artikel 9,10 und 11 fordern die Vertragsparteien auf gesetzliche Bestimmungen zu Tabakprodukten zu erlassen.

Neben der politischen Antwort [6,7], reorganisierte PM USA betriebsinterne wissenschaftliche Aktivitäten, um sich für die Regulierung von Tabakerzeugnissen zu wappnen. In einem Memo zur Einhaltung der FDA-Normen vom Juli 1992 stellte der Vizepräsident der PM Forschungs- und Entwicklungsabteilung fest: „Es ist wichtig, dass wir uns so schnell wie möglich auf potentielle Regulierungen vorbereiten" [ÜdA] [12]. PM hatte in seinen europäischen Versuchslaboratorien jahrzehntelang Rauchinhaltsstoffe analysiert und toxikologische Untersuchungen an Zigaretten vorgenommen [13]. Im August 1997 begann PM mit der Planung und Durchführung von Experimenten zur chemischen und toxikologischen Wirkung von 333 Zigarettenadditiven im Rahmen des „Projekt MIX" [14]. Projekt MIX umfasste die chemische Analyse von Rauchinhaltsstoffen, Untersuchungen zur In-vitro Mutagenität und -Zytotoxizität sowie zur In-vivo Inhalations-toxikologie. Diese Untersuchungen führten zu vier Publikationen, die im Januar 2002 in der Fachzeitschrift *Food and Chemical Toxicology* zusammen erschienen [15,16,17,18]. PM kam in den vier Arbeiten zu der Gesamteinschätzung: „Die in diesen Studien gefundenen statistisch signifikanten Veränderungen einiger Parameter weisen in dieselbe Richtung und waren ohne Einfluss auf die biologische Wirkungen, die gewöhnlich bei einer Exposition mit Zigarettenrauch beobachtet werden. Hinweise auf mögliche neue Effekte, die auf Zusatzstoffe zurückgeführt werden können, gab es nicht" [ÜdA] [15].

Die FDA sieht den Gebrauch von Zigarettenadditiven mit großer Besorgnis. So war eine der ersten Maßnahmen, die das neu gegründete Zentrum für Tabakprodukte der FDA 2009 ergriff, das Verbot aller Zigaretten-geschmacksstoffe, ausgenommen von Tabak und Menthol [9]. Der Beratende Wissenschaftsausschuss für Tabak-produkte der FDA kam 2011 zu dem Ergebnis, dass „die Entfernung von Mentholzigaretten aus dem Handel die öffentliche Gesundheit in den Vereinigten Staaten verbessern würde" [19].

PM benutze ab 2001 die bis dato unveröffentlichten Daten aus Projekt MIX in einer Reihe von Weißbüchern, um die Unbedenklichkeit einzelner Additive zu reklamieren. Neben „Die Bewertung des Einsatzes von Menthol als Zutat in Zigaretten" [ÜdA] [20] gehören dazu noch mindestens 7 vergleichbare Darstellungen z.B. zu Kakao [21], Propylen-glykol [22], Vanilleextrakt [23], Glycerin [24], Süßem Orangenöl [25] und Lakritzextrakt [26]. J. Daniel Heck, Wissenschaftler beim US Zigarettenhersteller Lorillard und Mitglied des Beratenden Wissenschaftsausschuss für Tabakprodukte der FDA [27], zitierte Ergebnisse aus Projekt MIX in einer 2010 erschienenen Übersichtsarbeit als Beleg für die Unbedenklichkeit von Menthol in Zigaretten [28].

Wir werteten Dokumente aus, die als Folge von Haftungsklagen in den Vereinigten Staaten gegen die Tabakindustrie öffentlich zugänglich gemacht wurden. Wir untersuchten die Entstehung und die Konzeption von Projekt MIX und unterwarfen die Ergebnisse einer zusätzlichen Auswertung. Danach ist fraglich, ob die Schlussfolgerung aus den vier Veröffentlichungen

zutreffend ist, dass die „Ergebnisse auf keine nennenswerten Auswirkungen der 333 Zusatzstoffe auf die Toxizität von Zigaretten hinweisen“ [15].

METHODE

Dokumente der Tabakindustrie

Wir untersuchten systematisch Dokumente der Tabakindustrie in der Legacy Tobacco Documents Library (LTDL; http://legacy.library.ucsf.edu/) der University of California, San Francisco. Die Dokumente wurden mittels etablierter Protokolle und Suchmethoden ausfindig gemacht, die auf einer Schneeballtechnik basieren, bei der nachfolgende Suchvorgänge auf vorgegangenen Suchergebnissen aufbauen [29,30,31]. Im Sinne der Fragestellung, ob die Tabakindustrie wissenschaftliche Forschung strategisch nutzt, um absehbare Tabakkontrollmaßnahmen abzuwenden, begannen wir die Suche mit den Begriffen “ingredients” (Inhaltsstoffe, Zutaten), “additives” (Zusatzstoffe) und “toxicity“ (Toxizität). Diese einleitende Recherche ergab tausende von Dokumenten, die mit den Möglichkeiten der optischen Zeichenerkennung der Legacy Tobacco Documents Library danach sortiert waren, wie oft der Suchbegriff in einem Dokument vorkommt. Eine qualitative Überprüfung dieser Dokumente in 50er bis 100er Paketen ermöglicht es die Suche zu präzisieren und Dokumente zusammen zustellen, die einen chronologischen Rahmen bilden für tiefer gehende Einblicke. Weitere relevante Dokumente ergab die Überprüfung benachbarter Dokument anhand sog. Bates-Nummern, sowie die Suche nach Schlüsselpersonen und an Orten, an denen schon andere relevante Dokumente gefunden wurden. Dieser sich wiederholende Prozess des Suchens, Überprüfens und Verfeinerns führte dazu, dass „Project MIX“ als Schlüsselbegriff identifiziert wurde. Etwa 500 relevante Dokumente wurden einer detaillierten Prüfung unterzogen, inklusive von Dokumenten in deutscher Sprache. Die Dokumente stammten aus den im Besitz von PM befindlichen Laboratorien in Europa, wo Projekt MIX durchgeführt wurde. Unsere Analyse begrenzt sich zwangsläufig auf Unterlagen, die in Folge von Haftungsklagen zur Verfügung stehen (etwa 60 Millionen Seiten zu der Zeit, zu der diese Studie durchgeführt wurde) [29].

ERGEBNISSE

Projektmerkmale

Entstehung von Project MIX

Seit langem führt die Tabakindustrie Tierversuche durch; mindestens seit den 1960er Jahren wurde, als Teil der Bemühungen eine “sichere Zigarette” zu entwickeln, versucht, krebserzeugende chemische Substanzen zu identifizieren und zu eliminieren [siehe Kapitel 4 in 32]. Seit dem Ende der 1970er Jahre führten PM Wissenschaftler Inhalationsversuche und Hautauftragungsstudien mit Mäusen durch, um potentielle Zigarettenadditive zu testen, einschließlich Harnstoff und Karamell [33], Kakao [34] und Glycerin [35]. In den 1980er Jahren unternahm PM routinemäßig chemische Rauchanalysen [36,37], Untersuchungen zur Mutagenität und Zytotoxizität (Ames-Test and Neutralrot-Aufnahme) [38,39], sowie In-vivo Toxizitätsmessungen an Nagern [40,41,42]. Die Methodik dieser Verfahren wurde dabei in den PM eigenen Forschungslabors „Institut für Biologische Forschung“ (INBIFO) in Köln und „Contract Research Center“ (CRC) in Brüssel / Belgien verfeinert [13].

Im März 1994 gaben Rechtsanwälte der Kanzlei Covington & Burling, die PM vertraten, ein Gutachten in Auftrag, das nach Auffassung der sechs verfassenden toxikologischen Experten zu dem Schluss kam, Zusatzstoffe wären unter den gegebenen Anwendungsbedingungen unbedenklich [43]. Im August 1994 wurde erstmals eine Liste veröffentlicht [44], die 599 bei der Herstellung von Zigaretten eingesetzte Zusatzstoffe aufführte, welche die Industrie als „sicher“ („safe“) einstufte [45]. 1997 trat PM der Gesellschaft für Toxikologie und dem Amerikanischen Toxikologenverband (American College of Toxicology) mit der Absicht bei, Wissenschaftler von PM zu positionieren, um die Studienergebnisse von INBIFO und der sechs toxikologischen Experten publik zu machen [46].

Kasten 1. Auszug aus den Leitlinien zur Umsetzung der Artikel 9 und 10 der FCTC [3, Absatz 3.1.2.2]

Inhaltsstoffe zur Erhöhung der Schmackhaftigkeit

Der strenge und reizende Charakter von Tabakrauch stellt eine entscheidende Barriere für das Experimentieren mit Tabakerzeugnissen und deren erstmaligen Konsum dar. Unterlagen der Tabakindustrie haben gezeigt, dass erhebliche Anstrengungen in die Minderung dieser ungünstigen Eigenschaften gesteckt worden sind. Die Strenge kann auf verschiedene Weise reduziert werden, unter anderem durch Zugabe verschiedener Inhaltsstoffe, durch Beseitigung von Stoffen mit bekanntermaßen reizenden Eigenschaften, durch Ausgleich der Reizwirkung mit anderen starken sensorischen Effekten oder durch Änderung der chemischen Eigenschaften der Emissionen von Tabakerzeugnissen durch Hinzufügung oder Entfernen bestimmter Stoffe.

Einige Tabakerzeugnisse enthalten Zuckerzusätze und Süßstoffe. Ein hoher Zuckergehalt verbessert die Geschmackseigenschaften von Tabakerzeugnissen für die Tabakkonsumenten. Beispiele für Zuckerzusätze und Süßstoffe, die in diesen Erzeugnissen verwendet werden, sind unter anderem Glukose, Melasse, Honig und Sorbitol.

Das Maskieren der Strenge von Tabakrauch mit Aromastoffen trägt zur Förderung und Aufrechterhaltung des Tabakgebrauchs bei. Beispiele für Geschmacks- und Aromastoffe sind unter anderem Benzaldehyd, Maltol, Menthol und Vanillin.

Gewürze und Kräuter können ebenfalls verwendet werden, um die Schmackhaftigkeit von Tabakerzeugnissen zu verbessern. Beispiele sind unter anderem Zimt, Ingwer und Minze.

Empfehlung:

Die Vertragsparteien sollten die Inhaltsstoffe, die zur Erhöhung der Schmackhaftigkeit bei Tabakerzeugnissen eingesetzt werden können, durch Verbote bzw. Einschränkungen regeln.

Inhaltsstoffe, die für die Herstellung von Tabakerzeugnissen unerlässlich sind und nicht deren Attraktivität beeinflussen, sollten nach dem jeweiligen Landesrecht geregelt werden.

1997 konzipierten Wissenschaftler von PM Projekt MIX, um die Wirkung von Zigarettenadditiven auf chemische Raucheigenschaften, die In-vitro Mutagenität und -Zytotoxität, sowie die biologische Aktivität in vivo zu untersuchen [14]. 2001 nahm die Zeitschrift *Food and Chemical Toxicology* die vier Arbeiten zur Veröffentlichung an, die auf den Resultaten von Projekt MIX resultierten und von PM Wissenschaftlern verfasst wurden [15,16,17,18]; das Zulassungsverfahren der Zeitschrift beschrieb Edward Carmines, Wissenschaftler bei PM und Leiter von Projekt MIX, Mitarbeitern gegenüber als „inside job“ (Insider Tätigkeit). „Wir wandten uns an eine Zeitschrift, deren Herausgeber uns kannte“ [ÜdA] [47].

Carmines Anmerkung ist wohlbegründet. Der damalige Herausgeber von *Food and Chemical Toxicology*, Joseph Borzelleca, gehörte dem wissenschaftlichen Beirat des „Ausschuss für Tabakforschung“ (Council for Tobacco Research) der Tabakindustrie an [48], ebenso wie dem „Wissenschaftlichen Beratergremium“ von PM (PM Scientific Advisory Board) [49]; zudem hatte Borzelleca eine lange Vorgeschichte mit Forschungs- und Beratungstätigkeiten für PM [siehe z.B.50,51]; tausende Dokumente der LTDL erwähnen Borzelleca. Der Mitherausgeber, P.J. van Bladeren, war 1991 Koautor einer Veröffentlichung bei der “International Conference: Priorities for Indoor Air Research and Action”, die durch die „Indoor Air International“ gefördert wurde [52]. Diese Gruppierung, die von Anwälten der Tabakindustrie geleitet wurde, war als Ausgangsbasis für eine Fachzeitschrift mit einem nominellen Peer-Review Verfahren gedacht, um Forschungsergebnisse zu veröffentlichen, die Standpunkte der Tabakindustrie zum Passivrauchen unterstützen sollten [53]. Susan Barlow, eine von zwei Gutachtern des Herausgebers, war auch Koautorin einer von PM finanzierten Übersichtsarbeit, die, nachdem von PM abgegebene Kommentare berücksichtigt wurden, den Hinweise auf einen Zusammenhang von Passivrauchexposition und plötzlichem Kindstod infrage stellte [54]. Elf Mitglieder des internationalen Redaktionsbeirates der Zeitschrift hatten Verbindungen zur Tabakindustrie: drei als Angestellte (A.W. Hayes, D.J. Doolittle [55,56,57] und Y.P. Dragan [58,59,60], zwei als Mitglieder des „Wissenschaftlichen Beratergremiums“ von PM (M. Pariza and S.L. Taylor [61]), weitere sechs hatten Forschungsgelder der Tabakindustrie angenommen oder verfügten über andere Verbindungen (O.I. Aruoma [62,63], A.R. Boobis [64,65,66], H.R. Glatt [67,68,69], G.C. Hard [70, 71], B. Pool-Zobel [72, 73] und H. Poulsen [74,75,76, 77]).

Auswahl der Additive

Unter Projekt MIX wurde die potentielle chemische und biologische Wirksamkeit von 333 Additiven untersucht, die bei der Zigarettenherstellung verwendet werden; sie wurden ausgewählt weil sie „Geschmacksstoffe repräsentieren, die weltweit von Philip Morris eingesetzt werden“ [ÜdA] [78]. Unter diesen 333 Additiven finden sich einige der 599, von denen bekannt war, dass sie bei der Herstellung von Zigaretten zugesetzt werden [44].

Die 333 für die Versuche unter Projekt MIX ausgewählten Additive, wurden in drei sich überschneidende *Zutaten-Gruppen* („Ingredient Groups“) eingeteilt [15]. Um die Testzigaretten zu erzeugen, wurden die Additive dem Tabak während der Zigarettenherstellung beigemengt. Kontrollzigaretten enthielten ausschließlich Tabak. Es gab zwei Zielwerte für jedes Additiv, einen „niedrigen“, der „angenähert der Menge entsprach, die bei kommerziellen Zigaretten einsetzt wird“ [ÜdA] [15] und einen “hohen”, der 1.5-3 mal des „niedrigen“ Wertes darstellte. (Eine Ausnahme stellte Menthol in *Zutaten-Gruppe 3* dar, das nur bei einem Wert von 18000 ppm in einem „hohen“ und „niedrigen“ Ansatz untersucht wurde, „weil physikalisch nicht mehr Substanz in die Testzigaretten eingebracht werden konnte“ [ÜdA] [15].) Carmines und Koautoren gaben in ihrer Veröffentlichung an [15], dass sie die Werte nur um den Faktor 1.5-3 variieren konnten, weil dies dem Höchstwert an Additiven entsprach, den die Zigaretten aufnehmen konnten und dabei noch „vergleichbar den Kontrollzigaretten abbrannten“ [ÜdA]. Um die Masse der Zigaretten konstant zu halten, wurde aus den Testzigaretten ein Teil des Tabaks entfernt.

Die spezifischen Bestandteile der Testzigaretten sind in tabellarischer Form in der veröffentlichten Arbeit aufgelistet [15] und können folgendermaßen zusammengefasst werden: *Zutaten-Gruppe 1*: 177 Additive; *Zutaten-Gruppe 2*: 277 Additive (incl. 117 aus Gruppe 1); *Zutaten-Gruppe 3*: größtenteils Menthol plus Kakaoschalen, Lakritzextrakt und Maissirup.

Das Verfahren oder die Logik für die Gruppen-zuordnung der Additive sind weder aus den Industrie-dokumenten noch aus den publizierten Arbeiten ersichtlich. Unklar ist wie und warum PM diese 333 Additive auswählte, ebenso warum die spezifischen Mengen und Zusammenstellungen eingesetzt wurden oder warum die anderen 266 Additive nicht für eine Testung in Projekt MIX ausgewählt wurden. In der Veröffentlichung [15] wird dazu lapidar erklärt, die Additive wären “repräsentativ für Zutaten, die weltweit eingesetzt würden“ und „von Lebensmittelqualität, oder besser“ und während des Herstellungsprozesses der Testzigaretten zugefügt wurden, um „dem tatsächlichen Herstellungsverfahren möglichst nahe zu kommen“ und „gestaltet worden, um gängigen kommerziellen Zigaretten zu ähneln“ [ÜdA].

In Vorbereitung von Projekt MIX fertigte PM USA sieben Testzigaretten unter folgenden Produktcodes: 97.MG.292 für Kontrollen, 97.MG.295 und 97.MG.296 für *Zutaten-Gruppe 1 niedrig* und *hoch*, 97.MJ.125 and 97.MJ.126 für *Zutaten-Gruppe 2 niedrig* und *hoch* und 97.MJ.127 and 97.MJ.128 für *Zutaten-Gruppe 3 niedrig* und *hoch* [79]. Die Testzigaretten wurden im Januar 1998 von einem Wissenschaftler des PM Produktprüflabors in den USA an INBIFO übergegeben, nachdem Validierungstests abgeschlossen waren [80].

Chemische Analyse von Rauchinhaltsstoffen

INBIFO bestimmte 51 Inhaltsstoffe im Haupt-stromrauch der Projekt MIX Zigaretten. Carmines zufolge wurden diese Rauchinhaltsstoffe aus Listen ausgewählt, die

von der amerikanischen Regierungsstelle für Produktsicherheit (U.S. Consumer Product Safety Commission (CPSC)) [81] und der Internationalen Agentur für Krebsforschung (International Agency for Research on Cancer [16] (IARC) Monographie 38) erstellt worden waren. Ein Memo des PM Wissenschaftlers Rick Solana aus dem Jahr 1997 [83,84] deutet an, dass PM eine Liste von 39 Inhaltsstoffen erstellte als "aktuelle Liste der Rauchinhaltsstoffe für die Hauptstromrauchanalyse bei Integritätsprüfungen von Produkten" [ÜdA], die auf den Listen der IARC und CPSC basierte und abgeglichen war mit der Liste krebserzeugender Substanzen des National Toxicology Program (NTP) von 1994.

Wir konnten nicht feststellen wie die 51 Inhaltstoffe für die Testungen in Projekt MIX festgelegt wurden. Die entsprechende, aus Projekt MIX abgeleitete Veröffentlichung [16] bestätigt, die Auswahl war „zu einem gewissen Grad subjektiv und basierte nicht auf einer ausgedehnten Risikobewertung" und legt dar, dass einige Analyte nicht eingeschlossen waren, weil „es keine eindeutige toxikologische Interpretation für möglicherweise auftretende Veränderungen gibt", oder weil „die Entwicklung von Testverfahren noch nicht abgeschlossen ist" [16]. Nimmt man die Listen der CPSC und der IARC zusammen, würden sich daraus 118 Analyte ergeben (Tabelle S-1 in Text S1). Solana stellte 1997 über die Liste der 39 fest, dass „polyaromatische Kohlenwasserstoffe…entsprechend der Aufmerksamkeit für diese Verbindungsklasse in der aktuellen Liste beibehalten werden. Man könnte jedoch überlegen sogar diese Verbindungen zu entfernen, sollte sich erweisen, dass sie konsequent die polyaromatischen Kohlenwasserstoffe beobachten, die auf der Liste empfohlen werden" [ÜdA] [83]. Diese Entscheidung könnte für das Weglassen einiger Inhaltsstoffe bei Projekt MIX verantwortlich sein.

Eine Liste der 72 Inhaltsstoffe, die nicht in Projekt MIX gemessen wurden, umfasst 11 polyzyklische aromatische Kohlenwasserstoffe (PAH). PAH sind besonders bedenklich, weil sie bei Menschen und Tieren Krebs und andere Erkrankungen verursachen [85,86,87]. Projekt MIX schloss auch die Analyse einiger Substanzen ein, die nicht von CPSC, IARC oder NTP gelistet sind, darunter die drei PAH Acenaphthen, Acenaphthylen, Benzo[ghi]perylen und weiter Naphthalin, m-, m-, und p-Kresol. Eines dieser PAH wurde auch in einer Analyse einzelner Rauchinhaltstoffe geprüft, die PM 1994 unter dem Codenamen URSUS durchführte [88].

Fehlende Messergebnisse von Ammoniak

Obgleich die Ammoniakwerte im Rauch der Testzigaretten in Projekt MIX bestimmt wurden, erwähnt die Publikation diese Ergebnisse nicht [16]. Interne Berichte vom Jahresbeginn 1999 zeigen [89,90], dass Ammoniak bestimmt wurde und verglichen mit dem Rauch von Kontrollzigaretten im Rauch der *Zutaten-Gruppe 1 hoch* signifikant erhöht war, ebenso wie in den beiden *Gruppen 2 hoch* und *niedrig*, aber signifikant erniedrigt vorlag im Rauch der mit Menthol versehenen *Zutaten Gruppen 3 hoch* und *niedrig* (Abbildung 1). Das Ergebnis der Ammoniakmessung aus dem abschließenden Report wegzulassen, erscheint sonderbar, angesichts der Tatsache, dass Ammoniak den pH-Wert des Tabaks anhebt (weniger sauer macht), und er dadurch leichter zu inhalieren ist [91]. Gleichzeitig nimmt dadurch die Bioverfügbarkeit des im Rauch vorhandenen Nikotins zu [92,93]. Es konnten keine pH-Wert Messungen der Testzigaretten aus Projekt MIX gefunden werden.

Ergebnisdarstellung im Verhältnis zum Rohkondensat (TPM)

Im Oktober 1998 änderten Wissenschaftler von INBIFO „auf Verlangen des Auftraggebers [PM USA]" [94], das Studienprotokoll von Projekt MIX, um die Ergebnisse im Verhältnis zum Rohkondensat (TPM) zu untersuchen und wiederzugeben; die ursprüngliche Planung vom Januar 1998 sah vor die Ergebnisse auf der Basis einer Zigarette darzustellen [95]. Vorgenommen wurden diese Veränderung acht Monate nachdem der INBIFO Wissenschaftler Wolf Reininghaus dem PM USA Manager für externe Studienprojekte Gerry Nixon [96,97] berichtete, dass Projekt MIX "Prototypen sich von den Kontrollen durch einen 10% höheren Rohkondensatanteil unterscheiden (was durch Verdünnung bei den subchronischen Studien ausgeglichen ist), [und] einen 20% höheren Akroleinwert (was bezogen auf TPM kein relevanter Unterschied ist)" [ÜdA] [96].

In der publizierten Arbeit werden die Ergebnisse der Rauchinhaltsstoffanalyse in einer Tabelle auf der Basis einer Zigarette dargestellt [16]; diese Daten zeigen, dass alle Testzigaretten, die Additive enthielten mehr Rohkondensat abgaben als die Kontrollzigaretten. In *Zutaten-Gruppen 1 hoch* und *niedrig* lagen die Werte um 15% and 13% höher, in den *Zutaten-Gruppen 2* um 17% und 28% und in den *Zutaten-Gruppen 3* um13% und 16% höher. Anstatt auf die biologische Bedeutung des angestiegenen Rohkondensats hinzuweisen, bezogen die Forscher von PM die Ergebnisse auf das Rohkondensat selbst und diskutierten, ob die Additive den Wert einzelner Toxine im Rauch pro Einheit des produzierten Rohkondensats erhöht hätten. Damit würde sich das Verhältnis (Toxin / TPM) verringern, sogar wenn Toxine und Rohkondensat mit den Additiven ansteigen würden, solange nur der Wert eines Toxins im Rauch einer Testzigarette weniger ansteigt als das Rohkondensat in dieser Zigarette.

Nach der Normalisierung des Rohkondensats zeigte die graphische Ergebnisdarstellung als Radarplot in der Publikation [16] nur bei 5 von 31 Toxinen eine Erhöhung in der *Zutaten-Gruppe 1*, 15 Werte dagegen fielen ab. Die Wissenschaftler von PM schlossen, dass aufgrund dieser Analyse - sowie entsprechender Analysen für die beiden anderen Gruppen - „die Beimischung dieser 333 gängigen Zutaten zu den Zigaretten in drei Gruppen nicht zur Rauchtoxizität beitrage, nicht mal auf dem unverhältnismäßig hohen Niveau, das in der vorliegenden Studie untersucht wurde" [ÜdA] [16].

Raucher rauchen jedoch keine Zigaretten, um ihre Rohkondensataufnahme zu regulieren. Sie rauchen eine

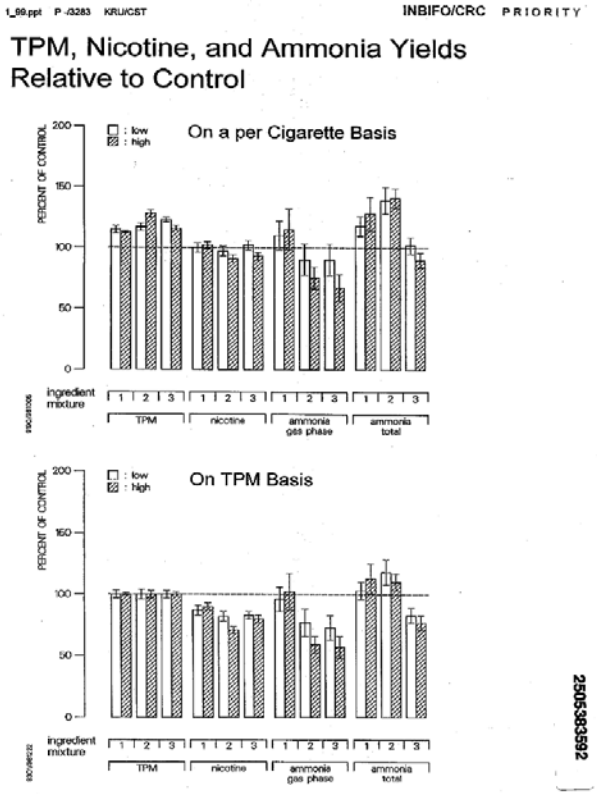

Abbildung 1. Diese Darstellung in einem internen INBIFO Report vom Januar 1999 gibt Ergebnisse der Ammoniakmessung in Projekt MIX wieder [89]. (Fehlerbalken entpr. Standardabweichungen). Alle drei *Zutaten-Gruppen* wiesen erhöhte Rohkondensatmengen (*TPM*) auf, höhere Ammoniakwerte in der Gasphase („ammonia gas phase“) in den *Zutaten-Gruppen 1* und niedrige Werte in *Zutaten-Gruppen 2* und *3* (dosisabhängig); die Werte wurden als statistisch signifikant bezeichnet. Gesamtammoniak („ammonia total“) war in den *Zutaten-Gruppen 1* und *2* erhöht. Weil Zigaretten mit Additiven mehr Rohkondensat erzeugten, minderte das (bzw. kehrte um) den angenommenen Effekt der Additive auf die Ammoniakproduktion, wenn diese auf das Rohkondensat bezogen wurden.

ganze Zigarette, im Allgemeinen, um sich eine bestimmte Dosis Nikotin zuzuführen [98,99,100]. Daher legten wir die von PM veröffentlichten Daten zugrunde [in 16, Tabelle 2], um einen entsprechenden Satz an Radarplots zu erstellen, welcher die Toxinmenge zum einen bezogen auf eine Zigarette darstellt (als Bruchteil der Kontrollzigaretten) und zum anderen das Verhältnis der Toxinwerte pro Einheit Nikotin für die Zigaretten mit Additiven verglichen mit den Toxinwerten pro Einheit Nikotin in den Kontrollzigaretten (Abbildungen 2-4). Werden die Daten aus Projekt MIX auf diese Weise dargestellt, entsteht ein ganz anderes Bild als in der Veröffentlichung von PM: auf der Basis einer Zigarette lagen nun 31 von 51 Chemikalien in mindestens einer der drei Zutatengruppen über der Kontrollgruppe, verglichen mit 17, die abgefallen waren; auf Basis einer Einheit Nikotin stiegen 37 an und 9 fielen ab.

Um 20% oder mehr über die Werte der Kontrollzigaretten stiegen 15 Chemikalien (Tabelle 1). Die Substanzen beinhalten eine Reihe von Tier- und Menschen-karzinogenen (Arsen, Cadmium, 1,3-Butadien, Blei, Formaldehyd, PAH), Atemwegsreizstoffe (z.B. Akrolien) und Zellgifte (Zyanwasserstoff, Kohlenmonoxid). Die Anzahl der Rauchinhaltsstoffe, die um mehr als 20% zunahmen, stieg von 7 bezogen auf das Rohkondensat auf 18, bezogen auf eine Zigarette und auf 23, bezogen auf Nikotin. Entsprechend fielen 24 Chemikalienwerte um mehr als 20%, wenn das Ergebnis bezogen auf das Rohkondensat dargestellt wurde, verglichen mit 12, wenn eine Zigarette und 9 wenn Nikotin die Grundlage bildeten (Abbildungen 2-4). In der Veröffentlichung wurden mit Ausnahme von 9 alle 24 gefallen Werte in den Radarplots dargestellt.

Unklar ist, warum man in der Veröffentlichung 19 der 51 getesteten Chemikalien von der Darstellung in Radarplots ausschloss [16], die als Grundlage der Diskussion dienen (Tabelle S-2 in Text S1). Zwei dieser ausgeschlossenen Verbindungen waren in den mit Additiven versehenen Zigaretten in der auf Zigaretten bezogenen Betrachtung signifikant gegenüber den Kontrollzigaretten angestiegen und zwar in allen Zutatengruppen: Benzo[-]fluoranthen und Benzo[ghi]perylen, beides PAH. Sechs weitere PAH, die im Rauch der *Zutaten-Gruppen 2* und *3* signifikant erhöht waren, fehlen ebenso in den veröffent-lichten Radarplots - Phenanthren, Anthrazen, Fluoranthen, Pyren, Benz[a]anthrazen und Chrysen - wie auch eine siebte Substanz - Indeno[1,2,3-cd]pyren -, die signifikant in *Zutaten-Gruppe 1* erhöht war. (Wir schließen alle 51 Rauchinhaltsstoffe in unsere Auswertung ein, siehe Abbildungen 2-4 und Tabelle 1.)

Subchronische In-vivo Inhalationsmessung

Die Exposition zur In-vivo Inhalationstoxikologie unter Projekt MIX erfolgten bei männlichen und weiblichen Sprague-Dawley Ratten über 90 Tage durch die Nase der Tiere entweder mit Frischluft (Plazebogruppe) oder mit verdünntem Hauptstromrauch aus den Test- und Kontrollzigaretten von Projekt MIX; nach einer 42-tägigen Postexpositionsphase wurden die Tiere getötet und histopathologisch untersucht [18]. Die Rauchexposition wurde so justiert, dass alle Ratten mit derselben Menge an Rohkondensat belastet wurden (150µg TPM/Liter), unabhängig davon mit welchen Zigaretten („Zutaten-„ oder Kontrollgruppe) der Rauch erzeugt wurde.

Die Wissenschaftler von PM berichteten über “einige geringfügige Veränderungen” der biologischen Wirkung bei Ratten, die mit additivbelastetem Zigarettenrauch behandelt wurden; allerdings ließen diese Veränderungen „kein ein-deutiges Muster erkennen“ und wären vermutlich „eine Folge statistischer Interferenz“ [ÜdA] [18]. Das Abstrakt der publizierten Arbeit kommt insgesamt zu dem Schluss, dass „die Ergebnisse [der 90-Tage Inhalationsstudie] andeuten, dass die Beimischung der 333 gängigen Zutaten, die den Zigaretten in den 3 Gruppen beigefügt wurden, die Toxizität des inhalierten Rauches nicht erhöhen würden“ [ÜdA].

Die von Projekt MIX wiedergegebenen Ergebnisse der tiertoxikologischen Untersuchungen [18] basieren auf einer kleinen Anzahl von Ratten in den einzelnen Testreihen (meist 9). Damit ist es möglich, dass statistisch signifikante Veränderungen der Endpunkte nicht erreicht werden,

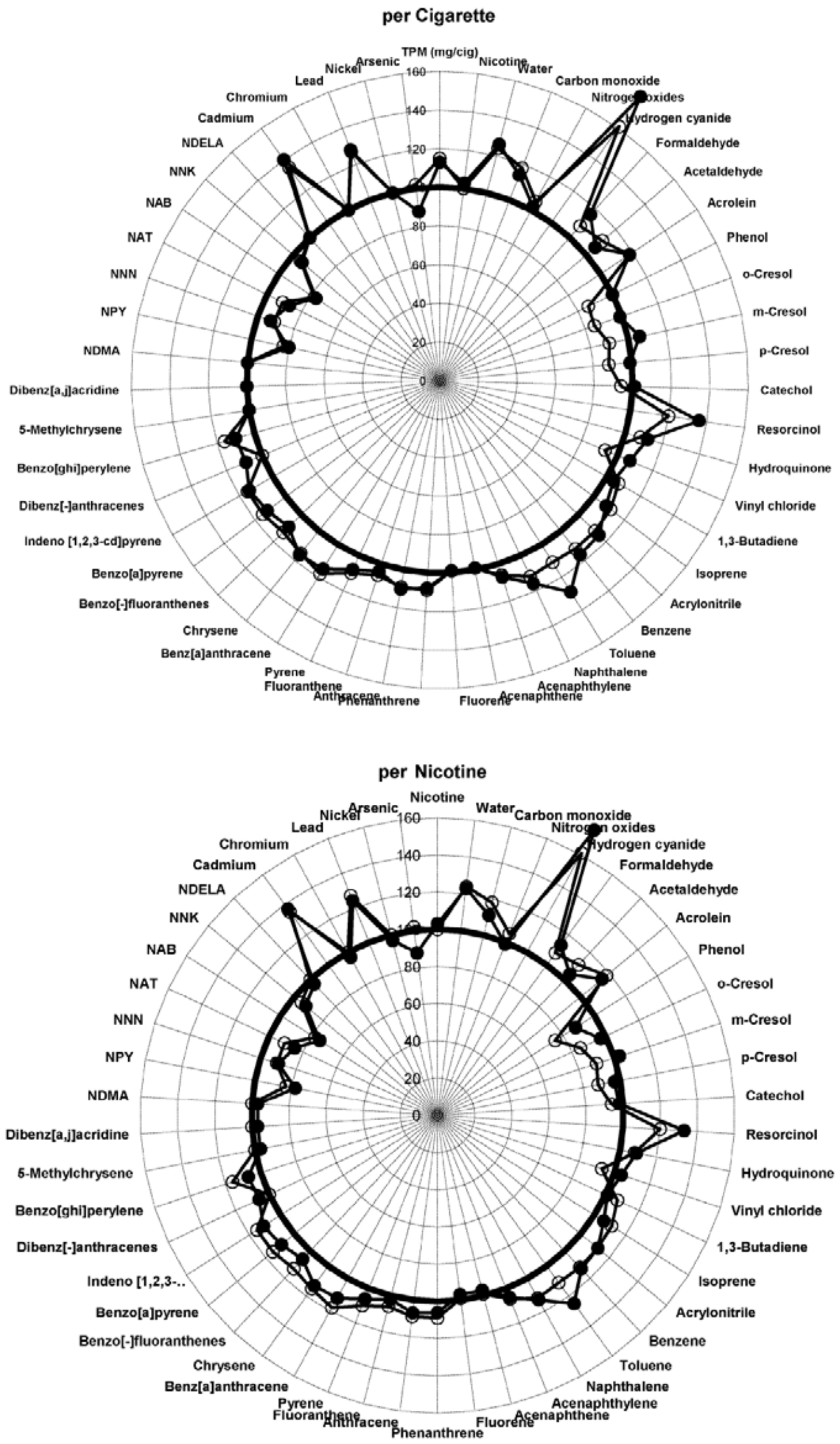

Abbildung 2. Graphische Darstellung der Rauchinhaltsstoffe pro Zigarette bzw. pro Einheit Nikotin für *Zutaten-Gruppe 1* verglichen mit den Kontrollzigaretten. *Niedrige* Additivwerte entsprechen offenen Kreisen, *hohe* Werte gefüllten Kreisen. Punkte außerhalb der 100% Kreislinie zeigen erhöhte Werte von Rauchinhaltsstoffen an, Punkte innerhalb entsprechen geringeren Werten.

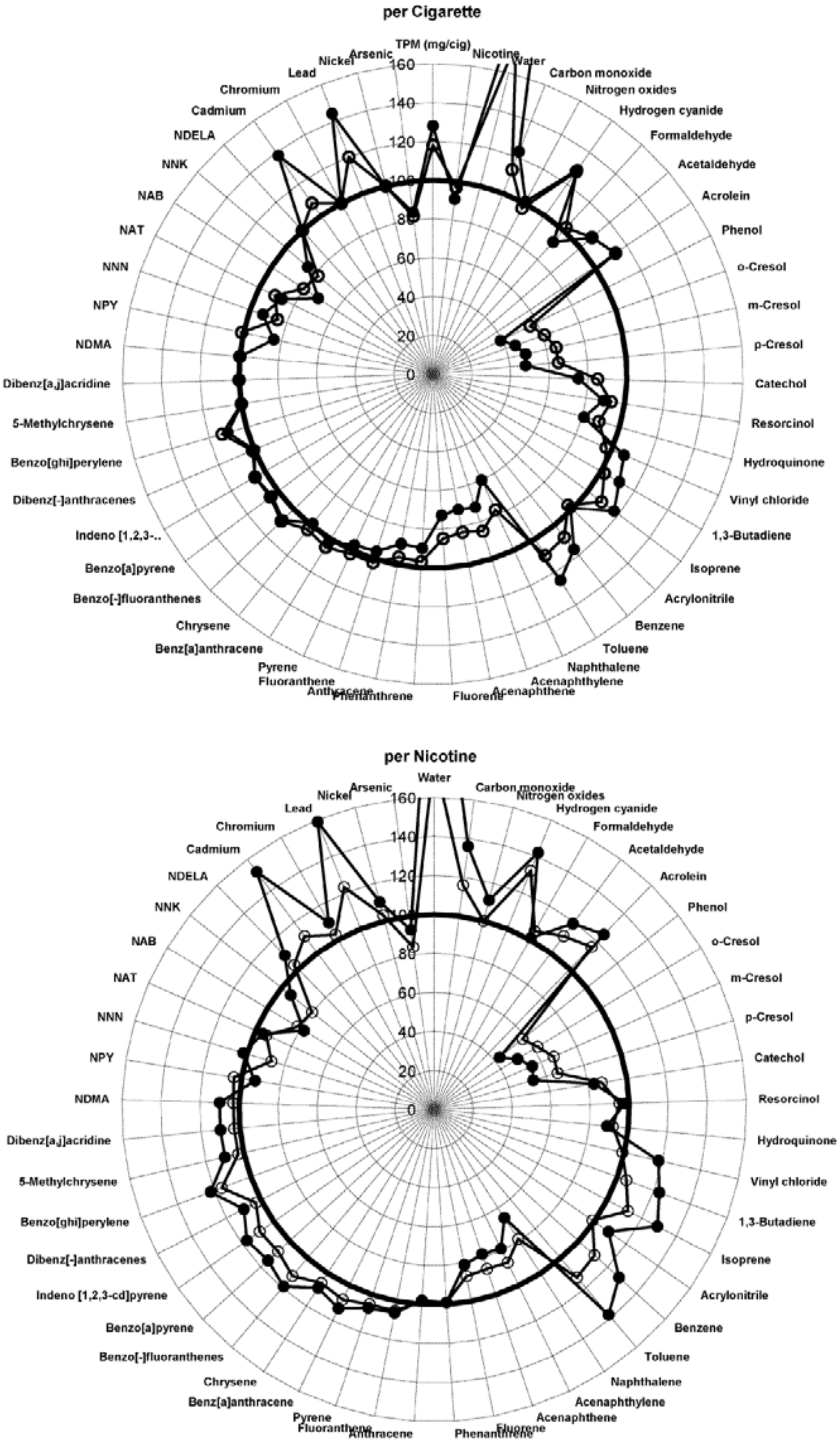

Abbildung 3. Graphische Darstellung der Rauchinhaltsstoffe pro Zigarette und pro Einheit Nikotin für *Zutaten-Gruppe 2* verglichen mit den Kontrollzigaretten. *Niedrige* Additivwerte entsprechen offenen Kreisen, *hohe* Werte geschlossen Kreisen. Punkte außerhalb der 100% Kreislinie zeigen erhöhte Werte von Rauchinhaltsstoffen an, Punkte innerhalb entsprechen geringeren Werten.

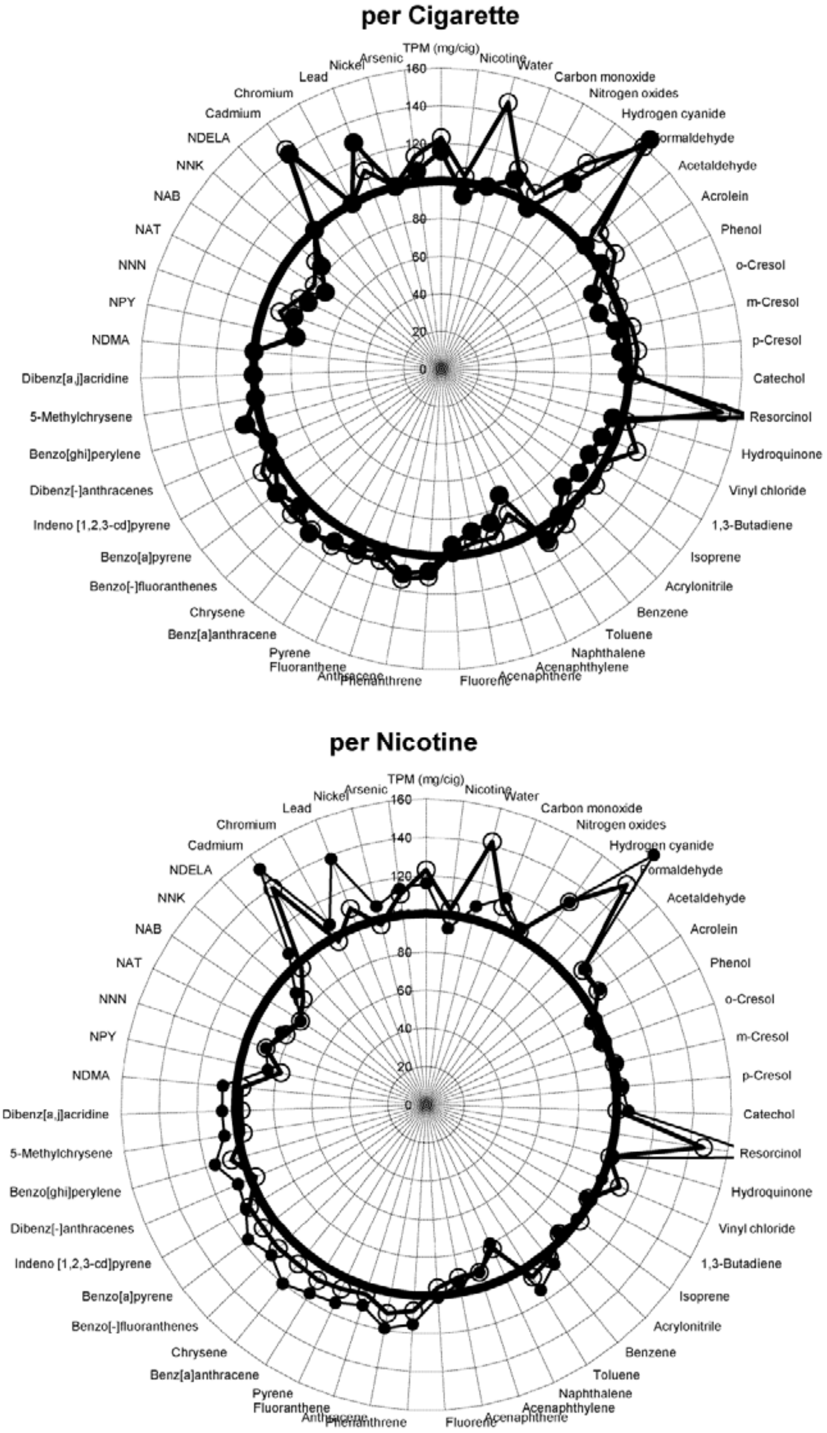

Abbildung 4. Graphische Darstellung der Rauchinhaltsstoffe pro Zigarette und pro Einheit Nikotin für *Zutaten-Gruppe 3* verglichen mit den Kontrollzigaretten. *Niedrige* Additivwerte entsprechen offenen Kreisen, *hohe* Werte geschlossen Kreisen. Punkte außerhalb der 100% Kreislinie zeigen erhöhte Werte von Rauchinhaltsstoffen an, Punkte innerhalb entsprechen geringeren Werten.

Chemikalie	pro mg Rohkondensat (TPM) (veröffentlichtes Radarplot [16])	pro Zigarette	pro mg Nikotin
Zutaten Gruppe 1			
Cyanwasserstoff	N H	N H	N H
Resorcin	H	N H	N H
Toluol			H
Cadmium	N	N H	N H
Blei		N H	N H
Arsen		H	
Zutaten Gruppe 2			
Rohkondensat (TPM)		H	H
Kohlenmonoxid		H	H
Cyanwasserstoff		N H	N H
Acrolein			H
Resorcin		N H	
1,3-Butadien			H
Isopren			H
Benzol			H
Toluol		H	H
Benzo[ghi]perylen			H
Cadmium		H	H
Blei		N H	N H
Zutaten Gruppe 3			
Rohkondensat (TPM)		N	N
Cyanwasserstoff		N H	N H
Formaldehyd	N H	N H	N H
Resorcin	N H	N H	N H
Chrysen			H
Cadmium	H	N H	N H
Blei		H	H

Tabelle 1. Chemikalien, die um mehr als 20% gegenüber Rauch aus Kontrollzigaretten erhöht waren

N = bei niedriger Additivmenge (entspr. der Verwendung in kommerziellen Zigaretten) H = bei hoher Additivdosierung (das 1.5-3 fache des niedrigen Wertes), leere Zellen zeigen Anstiege <20% an. Nur Chemikalien, die >20% anstiegen, wurden gelistet.

aufgrund tatsächlich ausbleibender Effekte, als wegen fehlender statistischer Teststärke. Um diese Möglichkeit zu überprüfen, setzten wir voraus, dass die veröffentlichten Ergebnisse (Durchschnittswerte und Standardabweichungen) einer unverzerrten Wiedergabe der Additiveffekte entsprechen. Basierend auf dieser Annahme untersuchten wir, wie eine statistische Berechnung ausfallen würde, wenn die Resultate von Projekt MIX aufgrund einer Stichprobengröße von 50, anstelle von 9 Ratten pro Gruppe ermittelt worden wären. (Während das tatsächliche Experiment mit einer größeren Stichprobe nicht präzise die selben Durchschnittswerte und Standardabweichungen ergeben dürfte wie in der Publikation, müssten die Abweichungen hingegen zufällig verteilt sein, angenommen die dargestellten Ergebnisse sind objektiv und korrekt). Wir entschieden uns für eine Stichprobengröße von 50 Tieren, weil PM eine wesentliche größere Stichprobe von 99 Ratten (von jedem Geschlecht) wählte, um in einer Studie die Effekte von Passivrauchexposition mit Dieselabgasen zu vergleichen [101,102]. Bei einem mitgeteilten Standardfehler des Mittelwerts von 0, nahmen wir die Standardabweichung mit 0.001 an. Unter Verwendung der berichteten Durchschnittswerte und Standardabweichungen überprüften wir in einer Varianzanalyse mit einem N von 50, ob die ANOVA-Testanalyse zu signifikanten Unterschieden führt ($p<0.05$); es folgte eine Mehrvergleichstestung gegen Kontrollzigaretten mit dem Holm-Sidak t-Test unter Annahme einer FWER (Familywise Error Rate) von 0.05. Alle Berechungen erfolgten mit dem Programm „Primer of Biostatistics (Version 6)" mit der *n*, Durchschnitts- und Standardabweichungsoption für ANOVA [103].

Diese Berechnung führte zu 194 statistisch signifikanten Änderungen verglichen mit 26, in der von PM publizierten Arbeit (Tabelle S-3 in Text S1). Dies legt nahe, dass bei besserer Teststärke ein wesentlich breiteres Spektrum an biologischen Effekten unter dem Einfluss der Additive zu Tage getreten wäre, als es in der von PM publizierten Arbeit dargelegt wurde [18]. Dies führt zu der Annahme, dass die Arbeit das toxische Potential von Rauch und Additiven erheblich unterschätzt.

DISKUSSION

Die Versuche der Projekt MIX Testreihen wurden scheinbar mit anerkannten Laborverfahren durchgeführt, so dass man wahrscheinlich von objektiven und korrekten Rohdaten ausgehen kann. Die grundlegende Schlussfolgerung hingegen, zu der die Wissenschaftler von PM aufgrund dieser Daten kommen, dass „Die Untersuchungen an Zigaretten mit beifügten Zutaten keinen nennenswerten Effekt dieser Zutaten auf die Toxizität der Zigaretten ergaben" [ÜdA] [15], spiegelt eher wieder, worauf die Ergebnisse der Rauchtoxizitätsmessungen bezogen wurden [16], und dass es den tiertoxikologischen Versuchen an Teststärke mangelt [18]. Ungeachtet dieser Schwächen wurden die Ergebnisse von Projekt MIX weit verbreitet, sowohl in der Wissenschaft [104, 105, 106, 107, 108, 109, 110, 111, 112, 113, 114, 115, 116], wie auch in einer breiteren Öffentlichkeit, dem (US) Insitute of Medicine gegenüber [117] und nicht zuletzt, weil sie von Wissenschaftlern anderer Tabakkonzerne zitiert wurden [118,119,120,121,122].

Die Schlussfolgerung, dass die Toxinmenge im Rauch durch Additive nicht erhöht würde, resultiert direkt aus der Tatsache, dass die mit Additiven belasteten Zigaretten 15-28% mehr Rohkondensat (TPM) abgaben als die Kontrollzigaretten, die nur Tabak enthielten. Alleine der Umstand, dass die Additive zu höheren Rohkondensatwerten führten, ist für sich genommen ein wichtiger Indikator für die gesteigerte Toxizität, weil das Rohkondensat im Zigarettenrauch das Risiko für kardiovaskuläre Erkrankungen erheblich steigert, und dies in Form einer steilen und hochgradig nichtlinearen Dosis-Wirkungsbeziehungen schon bei niedriger Belastung [123,124,125, 126]. Rohkondensat beeinträchtigt die physiologische Angiogenese und trägt zu extrauteriner Schwangerschaft, Spontanabort, Frühgeburt, plötzlichem Säuglingstod und verzögerter Wundheilung bei [127].

In einem Brief an den Herausgeber von *Food and Chemical Toxicology,* äußerten sich Vleeming et al. [128]

zur Tatsache, dass die Normalisierung der Toxinproduktion im Rauch ein falsches Bild über die Wirkung von Additiven auf die Rauchtoxizität entstehen lasse. Sie empfahlen, die Toxinmenge sollte auf die Tabakmenge einzelner Zigaretten bezogen werden. Weil PM die Tabakmenge reduzierte, wenn Additive zugegeben wurden, um die Masse der Zigaretten konstant zu halten, führt die Ergebnisdarstellung bezogen auf das Tabakgewicht zu einem noch stärkeren Anstieg der Toxizität als wir sie (in unseren Abbildungen 2-4) darstellen. Die Forscher von PM erklärten ihr Verfahren das Rohkondensat zu Normalisieren folgendermaßen: „Wir wählten das Rohkondensat als Grundlage unseres Vergleichs, um im Einklang mit den Tier- und In-vitro Studien dieser Publikationsserie zu sein", und „da sich Konsumenten entscheiden, Zigaretten entsprechend des Teergehaltes und Geschmacks zu rauchen, entschieden wir uns, die Daten auf das Rohkondensat zu beziehen, um den Effekt der Zutaten auf den Geschmack zu bestimmen" [ÜdA] [129]. Das erste Argument überrascht, da wie bereits erwähnt der ursprüngliche Plan für Projekt MIX nicht vorsah die Testergebnisse bezogen auf das Rohkondensat zu berechnen und darzustellen [130], sondern diese Berechnungsgrundlage erst gewählt wurde, nachdem sich zeigte, dass die Additive zu höheren Rohkondensatwerten führten, und das, obwohl die Zigaretten entsprechend weniger Tabak enthielten. Zudem ist allgemein anerkannt, dass Raucher rauchen, um die Zufuhr von Nikotin und nicht die Zufuhr von „Teer" zu kontrollieren [98,99,100], und auch bezogen auf den Nikotingehalt würden sich höhere Toxinwerte ergeben, als wenn die Toxinausbeute ins Verhältnis zum Rohkondensat gesetzt würden (Tabelle 1, Abbildungen 2-4).

Der Umstand, dass die In-vivo Versuche zur Toxikologie bei angeglichenen Rohkondensatwerten für den Rauch aller Zigarettentypen (um das Rohkondensat konstant zu halten) durchgeführt wurden, bedeutet zudem, wie der PM Wissenschaftler Reininghaus 1998 intern vermerkte [96], dass die Ratten, als sie den Rauch aus Zigaretten mit Additiven inhalierten, einer geringeren Toxinbelastung im Rauch ausgesetzt waren, als wenn die Exposition an eine andere Variable angepasst worden wäre, wie zum Beispiel die Nikotinzufuhr. Berücksichtigt man, dass die Tabakmasse der Zigaretten die Nikotinzufuhr bestimmt, könnte, wie Vleemings Betrachtungsweise nahe legt [128], die Unterbewertung der Toxinmenge, denen die Ratten ausgesetzt waren, übertragen auf menschliche Raucher, erheblich sein.

Ein weiteres Problem der In-vivo toxikologischen Untersuchungen ist darin zusehen, dass PM die Tiere mit festgelegten Expositionsgrenzwerten für Rohkondensat behandelte, wodurch sich das Verhältnis des Rohkondensats zu den Toxinen der Gasphase mit der jeweiligen *Zutaten-Gruppe* änderte. Wenn die Bildung von Toxinen der Gasphase weniger zunimmt als der Anstieg des Rohkondensats, dann würde eine Exposition der Ratten mit einem fixierten Rohkondensatwert die Exposition gegenüber gasförmigen Bestandteilen reduzieren. Für physiologische Endpunkte, die von diesen Gasen beeinflusst werden, könnte die Toxizität vermeintlich geringer ausfallen, wenn die Exposition auf das Rohkondensat und nicht auf Zigaretten bezogenen wird. Tatsächlich, als PM an INBIFO die Anweisung erteilte die Rauchinhaltsstoffe auf der Basis des Rohkondensats zu berechnen [94], wurde angemerkt, dass eine derartige Anpassung bereits „durch Verdünnung bei den subchronischen Studien ausgeglichen ist" [ÜdA] [96].

Fragen ergeben sich auch angesichts des Studiendesigns mit der relativ kurzen Expositionsphase von 90 Tagen, ebenso wie aufgrund der anschließenden 42tägigen Beobachtungsperiode nach Ende der Exposition. Ein Expositionsvergleich über die Wirkung von Passivrauch mit Dieselabgasen, den PM ebenfalls bei INBIFO durchführte, hatte je 99 Ratten beiderlei Geschlechts in jeder Gruppe, die 24 Monate (730 Tage) lang exponiert und anschließend 6 Monate (183 Tage) beobachtet wurden [101,102]. Die Teststärke von Projekt MIX zur Bestimmung der toxischen Additiveffekte hätte durch längere Expositions- und Nachbeobachtungsphasen verbessert werden können. Aber selbst mit diesen abwärtsgerichteten Testfehlern im Studiendesign, weisen unsere Ergebnisse noch nach, dass eine Untersuchung mit adäquater Teststärke eine große Anzahl toxischer Effekte bei den Ratten zu Tage gebracht hätte.

Die Industriedokumente zeigen, dass PM umfangreiche Forschung mit einzelnen Additive betrieb; eine Liste führt zwischen 1973 und 1995 170 Projekte auf, von denen etliche Einzeladditive untersuchten [131]. Zu den Projekten mit Einzeladditiven gehörte auch das INBIFO Projekt „Juice", bei dem Zitronensäure – eines der 333 Additive der vorliegenden Studie – in niedriger (2.6%), mittlerer (3.9%) und hoher (6.3%) Dosierung der Hauptzigaretteneinlage beigefügt wurde [132]. Die Mengen an Zitronensäure, über die bei Projekt MIX in der *Zutaten-Gruppe 1* berichtet wurden, lagen bei 44ppm in der *niedrigen* und 122ppm in der *hohen* Gruppe. Es ist unklar wie sich diese Zitronensäuredosierungen über die Studiengrenzen hinweg zueinander verhalten. Es gab auch Studien zu Milchsäure ("Project Milk") [133], Propylenglykol [134] und Kakao [34]. Auch bei diesen Studien wurden geringe Unterschiede zwischen den Gruppen getestet, in der Größenordnung einer nur 2-3 fachen Differenz.

Projekt MIX umfasste auch Untersuchungen zur In-vitro Mutagenität und -Zytotoxität [17], bei denen eine 1996 von PM entwickelte standardisierte Testbatterie, einschließlich Ames-Test zum Einsatz kam [14, 135, 136, 137]. Nicht ganz überraschend ergaben diese Testungen, dass alle Zigaretten genotoxisch und mutagen waren, ob sie Additive enthielten oder nicht. Diese Tests sind jedoch Screening-Verfahren und keine sensitiven Dosis-Wirkungs-Messungen [138, 139, 140, 141]. Deshalb sind sie ungeeignet, um Veränderung der Toxizität durch Additive zu quantifizieren. Abgesehen von dem unwahrscheinlichen Fall, dass Additive die Genotoxizität und Mutagenität des Tabaks eliminieren würden, rechtfertigt der fehlende Nachweis erhöhter Toxizität durch Additive nicht den Schluss, dass die "In-vitro Mutagenität und -Zytotoxizität von Zigerettenrauch durch die Zugabe von Zutaten nicht gesteigert wurde" [17].

Wissenschaftler von British American Tobacco (BAT) führten 2004 ein ähnliches Projekt durch, das ebenfalls in *Food and Chemical Toxicology* publiziert wurde [142,143,144]. Die BAT Studie untersuchte 482 Additive in verschiedenen Zusammensetzungen „mit oder über den gängigen Höchstwerten, die in Zigaretten vorkommen, die von BAT vertrieben werden“ [ÜdA]. Die Testungen umfassten die Analyse von 45 ausgewählten Rauchinhaltsstoffen im Hauptstromrauch von Test-zigaretten, In-vitro Mutagenitäts und –Zytotoxitäts-messungen (Ames-Test, In-vivo Mikrokerntest bei Säugetieren und Neutralrot-Aufnahme) und einen 90-Tage Inhalationstest bei männlichen und weiblichen Sprague-Dawley Ratten (10 Ratten pro Gruppe). Bei vergleichbaren Rauchexpositionsraten (ca. 20% weniger als bei den Ratten in der PM-Studie), berichteten die Wissenschaftler von BAT über ähnlich gravierende histopathologische Befunde wie in Projekt MIX: erhöhte Dichte von Gobletzellen im Atemwegsepithel, gesteigerte Hyperplasie bzw. Degeneration in der Nasenhöhle sowie gesteigerte Metaplasien der Epiglottis. Ähnlich wie die Wissenschaftler von PM, verharmlosten die Forscher von BAT die Befunde, indem sie schlossen, sie wären unstimmig und würden darauf hindeuten, dass die Reaktion der Tiere auf die Rauchexposition zwischen Test- und Kontrollzigaretten nicht zu unterscheiden sei.

Manipulationen bei der Darstellung von Forschungsergebnissen, wie in den aus Projekt MIX abgeleiteten Publikationen [15,16,17,18], sind nichts Neues für die Tabakindustrie: Industriewissenschaftler verfahren so seit langem bei einer Vielzahl von Streitfragen über die Wirkung des Passivrauchens [145, 146, 147, 148, 149]. Während die Methoden zur Datenerhebung an sich einwandfrei erscheinen, trifft dies für die Art und Weise wie die Daten ausgewertet und interpretiert werden nicht zu. Eine bedeutsame Erkenntnis unserer Analyse ist, dass Wissenschaftler und Regulierungsbehörden nicht für bare Münze nehmen sollten, was Wissenschaftler der Tabakindustrie als Forschungsergebnisse darbieten, sowenig wie externe von der Tabakindustrie finanzierte Forschungsarbeiten, und auch nicht was in Fachzeitschriften veröffentlicht wurde, die von der Tabakindustrie dominiert werden, wie dies bei *Food and Chemical Toxicology* der Fall ist. Die konsequente Umsetzung von Artikel 5.3 des WHO-Rahmenübereinkommens zur Eindämmung des Tabakgebrauchs (FCTC), das den politischen Entscheidungsprozess vor Einflüssen und Manipulationen der Tabakindustrie zu schützen versucht [150], unterstreicht, dass angesichts der Ergebnisse von Projekt MIX - und den entsprechenden Veröffentlichungen von BAT - bei Gesetzgebungsverfahren Skepsis angebracht ist. Für die FDA, die WHO und Regulierungsbehörden in Ländern, die an der Umsetzung der Artikel 9, 10 und 11 der FCTC arbeiten, wird es von großer Bedeutung sein, darauf zu bestehen, alle Entwürfe des Studienprotokolls zusammen mit den Rohdaten zu erhalten, um der hier dargelegten Problematik zu begegnen. Dabei ist besonders darauf zu achten den Tabakkonzernen nicht zu gestatten, Rechtsanwälte einzuschalten, um die Offenlegung zu verhindern. Wenn man die Annahme von PM teilt, dass die unter Projekt MIX in Gruppen getesteten Additive den „in handelsüblichen Zigaretten ähneln“ [ÜdA] [15], könnten die von PM gesammelten Daten zur Entwicklung gesetzlicher Richtlinien bezüglich dieser 333 Additive herangezogen werden. Der Umstand, dass die Wissenschaftler bei Projekt MIX die Additive nicht getrennt, sondern in Gruppen untersucht haben, gibt Raum für die Überlegung, dass Additive entweder antagonistisch oder synergistisch wirken. Von synergistischer Wirkung auszugehen ist dabei entscheidend. Das angenommene Risiko für Lungenkrebs durch Tabakrauch aufgrund summierter Additiveffekte ist wesentlich geringer als das epidemiologisch ermittelte Risiko [114]. Dies kann damit erklärt werden, dass nicht alle Karzinogene und kardial wirksamen Toxine aus dem Tabakrauch identifiziert sind und man von Interaktionen verschiedener Bestandteile in komplexen Gemischen wie Tabakrauch ausgehen muss.

Es muss auch betont werden, dass die in Projekt MIX und den vergleichbaren Experimenten von BAT [142,143,144] stillschweigend eingeschlossene Definition von “harm“ (Schaden), nämlich die direkte kurzfristige toxische Wirkungen von Additiven, nur eine Dimension der Additivwirkung auf die Krankheitsentstehung darstellt. Additive verändern nämlich die sensorischen und pharmakologischen Eigenschaften der Zigaretten (Kasten 1), wodurch diese für den Konsumenten milder und angenehmer erscheinen [3,151,152]. Dies wirkt sich beim Einstieg in das Rauchen und im Falle einer Entwöhnung aus und führt zur Konsumsteigerung und so zu mehr tabakassoziierten Erkrankungen [4,19]. In der Tat war es vor allem die Bedeutung für die allgemeine Gesundheit, die den Beratenden Wissenschaftsausschuss für Tabakprodukte der FDA zu der Einschätzung brachte, dass „die Entfernung von Mentholzigaretten aus dem Handel die öffentliche Gesundheit in den Vereinigten Staaten verbessern würde“ [ÜdA] [19].

Limitierungen

Wie im Methodenteil erwähnt, basiert diese Untersuchung auf Dokumenten, die PM in der Folge von gegen die Firma erhobenen Haftungsklagen zur Verfügung stellen musste. Während es uns gelang verschiedene Entwürfe und detaillierte Ergebnisse von Projekt MIX zu finden, anhand derer wir zum Beispiel feststellen konnten, dass das Protokoll zur Datenauswertung geändert wurde, nachdem Resultate mit erhöhten Rohkondensatwerten vorlagen, fehlen entscheidende Informationen: auf welcher Grundlage hat PM die Additive ausgesucht, die in der Studie untersucht wurden, wie wurden Dosierungen und Gruppenzusammenstellung festgelegt und was erklärt die Stichprobengröße der toxikologischen Testungen? Diese Umstände schränken die verfügbare Informationsauswertung ein und unterstreichen, dass die FDA ihre neugewonnenen Befugnisse einsetzen sollte, um alle Datenquellen über die Additivtestungen und andere Merkmale des Herstellungsverfahrens zu erhalten, um sie einer Überprüfung zugänglich zu machen.

Schlussfolgerung

Die vorliegende Analyse ergibt, dass Rohkondensat und zahlreiche Toxine erheblich zunehmen, wenn Zigaretten Additiven zugesetzt werden. Vorausgesetzt die toxikologischen Resultate von Projekt MIX spiegeln die biologische Wirkung unverzerrt wieder, belegen diese Daten zahlreiche negative biologische Konsequenzen. (Die Ergebnisse unterhalb des Signifikanzniveaus sind eher mangelnder Teststärke als nicht vorhandener Wirkung zuzuschreiben). Insbesondere Zulassungsbehörden wie die FDA und vergleichbare Einrichtungen andernorts, welche Artikel 9, 10 und 11 der FCTC umsetzen, könnten die Daten aus Projekt MIX verwenden, um den Einsatz dieser 333 Zusatzstoffe (einschließlich Menthol, der Hauptkomponente aus *Zutaten-Gruppe 3*) zu unterbinden. Jedem Tabakkonzern stünde es natürlich frei, bei der FDA oder anderen Regulierungs-behörden einen Antrag auf Zulassung für die Wieder-einführung eines Zusatzstoffes zu stellen, wenn dazu valide Daten aus Untersuchungen mit adäquater Teststärke vorgelegt werden könnten, die belegen, dass ein Additiv unbedenklich für die Gesundheit ist.

DANKSAGUNG

Wir danken Melanie Marty, David Kessler, Paul Blanc, John Froines und Neal Benowitz für hilfreiche Anregung bei der Erstellung des Manuskripts.

ABSTRAKT IN ALTERNATIVSPRACHE

Abstrakt in Alternativsprache S1
Deutsche Abstraktübersetzung von TK
Abstrakt in Alternativsprache S2
Chinesische Abstraktübersetzung von Ting Ting Yao
Abstrakt in Alternativsprache S3
Französische Abstraktübersetzung von Martine Wagnac
Abstrakt in Alternativsprache S4
Spanische Abstraktübersetzung von Ernesto Sebrie
Abstrakt in Alternativsprache S5
Finnische Abstraktübersetzung von Heikki Hiilamo
Abstrakt in Alternativsprache S6
Russische Abstraktübersetzung von Lyudmila Popova

TEXT S1
Drei Tabellen

Tabelle S-1. Kombinierte Liste für die Analyse von Rauchinhaltsstoffen der CPSC, IARC und aus Project MIX, Tabelle S-2: 19 Rauchinhaltsstoffe, die nicht in den veröffentlichten Radarplots aufgeführt sind und Tabelle S-3: Statistisch signifikante toxikologische Veränderungen durch Zigarettenrauch der drei Additivgruppen mit je zwei unterschiedlichen Dosierungen.

TEXT S2
Deutsche Übersetzung des Artikels von TK

TEXT S3
Deutsche Übersetzung von Text S1 von TK

LITERATUR

1. Wakeham H. Hazleton Laboratories. 22 Jul 1963. Bates number 1001606422. Available: http://legacy.library.ucsf.edu/tid/kps08e00. Accessed 14 November 2011.
2. Cigarette Labeling and Advertising Act (P.L. 98-474). Available: http://uscode.house.gov/download/pls/15C36.txt. Accessed 14 November 2011.
3. World Health Organization. Partial guidelines for implementation of Articles 9 and 10 of the WHO Framework Convention on Tobacco Control (Regulation of the contents of tobacco products and Regulation of tobacco product disclosures). Available: http://www.who.int/fctc/guidelines/Decisions9and10.pdf. Accessed 4 June 2011.
4. Lee YO, Glantz SA (2011) Menthol: putting the pieces together. Tob Control 20: ii1-ii7.
5. Family Smoking Prevention and Tobacco Control and Federal Retirement Reform (2009) US Public Law 111-31. Available: http://www.gpo.gov/fdsys/pkg/PLAW-111publ31/pdf/PLAW-111publ31.pdf. 111th Congress. Washington (D.C.): G.P.O. Accessed 14 November 2011.
6. McDaniel PA, Malone RE (2005) Understanding Philip Morris's pursuit of US government regulation of tobacco. Tob Control 14: 193-200.
7. McDaniel PA, Smith EA, Malone RE (2006) Philip Morris's Project Sunrise: weakening tobacco control by working with it. Tob Control 15: 215-223.
8. Glantz SA, Barnes R, Eubanks SY (2009) Compromise or capitulation? US Food and Drug Administration jurisdiction over tobacco products. PLoS Med 6: e1000118. doi:10.1371/journal.pmed.1000118
9. US Food and Drug Administration, Department of Health and Human Services (2009) Candy and fruit flavored cigarettes now illegal in United States; step is first under new tobacco law. Available: http://www.fda.gov/NewsEvents/Newsroom/PressAnnouncements/ucm183211.htm. Accessed 24 July 2011.
10. Rees VW, Kreslake JM, O'Connor RJ, Cummings KM, Parascandola M, et al. (2009) Methods used in internal industry clinical trials to assess tobacco risk reduction. Cancer Epidemiol Biomarkers Prev 18: 3196-3208.
11. World Health Organization. WHO Framework Convention on Tobacco Control. Available: http://www.who.int/fctc/en/index.html. Accessed 12 September 2008.
12. Ellis CL, Milby DL (1997) 970000 FDA Compliance Preparation. 9 Jul 1997. Bates number 2078468223/2078468224. Available: http://legacy.library.ucsf.edu/tid/asl75c00. Acccessed14 November 2011.
13. Diethelm PA, Rielle JC, McKee M (2005) The whole truth and nothing but the truth? The research that Philip Morris did not want you to see. Lancet 366: 86-92.
14. Nixon G. 21 Aug 1997. Bates number 2501950681/2501950682. Available: http://legacy.library.ucsf.edu/tid/gke81c00. Accessed 14 November 2011.
15. Carmines EL (2002) Evaluation of the potential effects of ingredients added to cigarettes. Part 1: cigarette

design, testing approach, and review of results. Food Chem Toxicol 40: 77-91.
16. Rustemeier K, Stabbert R, Haussmann HJ, Roemer E, Carmines EL (2002) Evaluation of the potential effects of ingredients added to cigarettes. Part 2: chemical composition of mainstream smoke. Food Chem Toxicol 40: 93-104.
17. Roemer E, Tewes FJ, Meisgen TJ, Veltel DJ, Carmines EL (2002) Evaluation of the potential effects of ingredients added to cigarettes. Part 3: in vitro genotoxicity and cytotoxicity. Food Chem Toxicol 40: 105-111.
18. Vanscheeuwijck PM, Teredesai A, Terpstra PM, Verbeeck J, Kuhl P, et al. (2002) Evaluation of the potential effects of ingredients added to cigarettes. Part 4: subchronic inhalation toxicity. Food Chem Toxicol 40: 113-131.
19. FDA Tobacco Products Scientific Advisory Committee. Menthol cigarettes and public health: review of the scientific evidence and recommendations (as reviewed at the TPSAC meeting on March 18, 2011). Available: http://www.fda.gov/downloads/AdvisoryCommittees/CommitteesMeetingMaterials/TobaccoProductsScientificAdvisoryCommittee/UCM247689.pdf. Accessed 17 April 2011.
20. Product Integrity, Philip Morris USA (2001) Evaluation of menthol for use as a cigarette ingredient. 03 Oct 2001. Bates number 2067617005/2067617095. Available: http://legacy.library.ucsf.edu/tid/nox75a00. Accessed 14 November 2011.
21. Product Integrity, Philip Morris USA (2001) Evaluation of cocoa for use as a cigarette ingredient. 03 Oct 2001. Bates number 2067637337/2067637404. Available: http://legacy.library.ucsf.edu/tid/wcc34a00. Accessed 14 November 2011.
22. Philip Morris USA (2001) Evaluation of propylene glycol for use as a cigarette ingredient. 03 Oct 2001. Bates number 2085543332/2085543389. Available: http://legacy.library.ucsf.edu/tid/rrn43a00. Accessed 14 November 2011.
23. Product Integrity, Philip Morris USA (2001) Evaluation of vanilla extract for use as a cigarette ingredient. 03 Oct 2001. Bates number 2067617286/2067617380. Available: http://legacy.library.ucsf.edu/tid/lox75a00. Accessed 14 November 2011.
24. Product Integrity, Philip Morris USA (2001) Evaluation of glycerol for use as a cigarette ingredient. 03 Oct 2001. Bates number 2067636691/2067636778. Available: http://legacy.library.ucsf.edu/tid/jec34a00. Accessed 14 November 2011.
25. Product Integrity, Philip Morris USA (2001) Evaluation of sweet orange oil for use as a cigarette ingredient. 03 Oct 2001. Bates number 2067617122/2067617209. Available: http://legacy.library.ucsf.edu/tid/mox75a00. Accessed 14 November 2011.
26. Product Integrity, Philip Morris USA (2001) Evaluation of licorice extract for use as a cigarette ingredient. 03 Oct 2001. Bates number 2067661381/2067661422. Available: http://legacy.library.ucsf.edu/tid/rha34a00. Accessed 14 November 2011.
27. US Department of Health & Human Services (2011) Tobacco Products Scientific Advisory Committee. US Food and Drug Administration. Available: http://www.fda.gov/AdvisoryCommittees/CommitteesMeetingMaterials/TobaccoProductsScientificAdvisoryCommittee/default.htm. Accessed 10 December 2010.
28. Heck JD (2010) A review and assessment of menthol employed as a cigarette flavoring ingredient. Food Chem Toxicol 48: S1-38.
29. Anderson SJ, McCandless PM, Klausner K, Taketa R, Yerger VB (2011) Tobacco documents research methodology. Tobacco Control 20: ii8-ii11.
30. Bero L (2003) Implications of the tobacco industry documents for public health and policy. Annu Rev Public Health 24: 267-288.
31. Malone RE, Balbach ED (2000) Tobacco industry documents: treasure trove or quagmire? Tob Control 9: 334-338.
32. Glantz SA, Barnes DE, Bero L, Hanauer P, Slade J (1996) The cigarette papers. Berkeley: University of California Press. pp. 219-224
33. Author unknown [Document from Thomas Osdene's files at Philip Morris] (1978) Test Protocol LTF. 13 Feb 1978. Bates number 1000024814/1000024816. Available: http://legacy.library.ucsf.edu/tid/cge97e00. Accessed 14 November 2011.
34. Kuhn D, Romer E, Schmatz E, Teredesai A, Thomas C (1982) Report a 0500/3023. Comparative study on whole smoke condensates from cigarettes with and without addition of cocoa in the mouse skin painting assay. 01 Mar 1982. Bates number 2028790638/2028790972. Available: http://legacy.library.ucsf.edu/tid/zkz74e00. Accessed 14 November 2011.
35. Blake C, Bosch V, Droz A, Posset L (1987) The effect of the humectants glycerol and propylene glycol on mainstream and sidestream smoke deliveries of acrolein, formaldehyde, acetaldehyde, acetone and propionaldehyde. 00 Feb 1987. Bates number 2023186677/2023186680. Accessed 14 November 2011.
36. Genoud Y, Lecoultre E, Moser F, Murray M, Piade JJ, et al. (1982) Project title: Analytical Investigations 820417 - 820518. 27 May 1982. Bates number 2501308073/2501308074. Available: http://legacy.library.ucsf.edu/tid/wyc29e00. Accessed 14 November 2011.
37. Blake CJ, Losee B, Oey J, Posset L, Rychener M, et al. (1986) Final report project slow 77-c1 chemical and biological testing of prototype cigarettes 72-c1 and 77-C1. 10 Jan 1986. Bates number 2022150326/2022150352. Available: http://legacy.library.ucsf.edu/tid/yyd59e00. Accessed 14 November 2011.
38. Stinn W (1989) Concept. 23 May 1989. Bates number 2028991555/2028991564. Available: http://legacy.library.ucsf.edu/tid/kmf69e00. Accessed 14 November 2011.
39. Gugel H, Heil M, Oey J, Romer E, Zzdroe (1982) Proposal a 0268/2074 4-day cytotoxicity study

exposure to whole smoke and gas/vapor phase of cigarettes coral-a and coral-b, and of standard reference cigarette 2r1f of human lung cells pan-L3. 00 1982. Bates number 2501217555/2501217604. Available: http://legacy.library.ucsf.edu/tid/zju32e00. Accessed 14 November 2011.
40. Reininghaus W (1990) Achievements and proposed research projects. 15 Nov 1990. Bates number 2029173277/2029173308. Available: http://legacy.library.ucsf.edu/tid/ndx37e00. Accessed 14 November 2011.
41. Philip Morris (1984) Table sidestream smoke doses in in vivo inhalation studies. 10 Jul 1984. Bates number 2501668618. Available: http://legacy.library.ucsf.edu/tid/vin49e00. Accessed 14 November 2011.
42. Bachmayer D (1989) Concept sister chromatid exchange and chromosome aberration inducing activities of ethylnitrosourea and formaldehyde in nasal and tracheal epithelial cells: acute sub acute study on rats (Pt). 00 1989. Bates number 2028826323/2028826333. Available: http://legacy.library.ucsf.edu/tid/kco27e00. Accessed 14 November 2011.
43. Doull J, Frawley JP, George W, Loomis T, Squire RA, et al. (1994) A safety assessment of ingredients added to tobacco in the manufacture of cigarettes. 00 Mar 1994. Bates number 2073910155/2073910163. Available: http://legacy.library.ucsf.edu/tid/acm05a00. Accessed 14 November 2011.
44. American Tobacco Company, Brown & Williamson Tobacco Corporation, Liggett Group Inc., Lorillard Inc., Philip Morris Incorporated et al. (1993) List of ingredients added to tobacco in the manufacture of cigarettes (as of 931231). 31 Dec 1993. Bates number 89284138/89284169. Available: http://legacy.library.ucsf.edu/tid/ayy10e00. Accessed 14 November 2011.
45. Tobacco Reporter (1994) Flavorings. Safe. cig flavorings not a risk, says independent panel. Tr Staff Report. Jul 1994. Bates number 523319071/523319078. Available: http://legacy.library.ucsf.edu/tid/sis20d00. Accessed 14 November 2011.
46. Philip Morris (1998) 6 June 1998. Bates number 2501826284/2501826291. Available: http://legacy.library.ucsf.edu/tid/sac90c00. Accessed 14 November 2011.
47. Carmines EL (2001) L.S.R.O. Presentation. 06 Dec 2001. Bates number 2067636959/2067637071. Available: http://legacy.library.ucsf.edu/tid/ekw86a00. Accessed 14 November 2011.
48. Philip Morris Incorporated (2011) Philip Morris glossary of names. http://legacy.library.ucsf.edu/glossaries/pm_gloss_bo.jsp. Accessed 16 April 2011.
49. Philip Morris Incorporated (1999) Letter to Borzelleca confirming agreement to serve on PM's Scientific Advisory Board. 02 Nov 1999. Bates number 3001109471. Available: http://legacy.library.ucsf.edu/tid/csu91g00. Accessed 14 November 2011.
50. Philip Morris Incorporated (1995) Letter to Houghton Sr. (Consultant agreement for Borzelleca). 13 Jan 1995. Bates number 2059674110. Available: http://legacy.library.ucsf.edu/tid/hds22d00. Accessed 14 November 2011.
51. Borzelleca J, Cox RH. Amendment number 1 to agreement dated 19981001 between Pharmacology & Toxicology, Inc. And Philip Morris Incorporated. 08 Jun 1999. Bates number 3001337718-3001337719. Available: http://legacy.library.ucsf.edu/tid/lem71h00. Accessed 14 November 2011.
52. (1991) International conference: priorities for indoor air research and action. Bates number 400083804-400083813. Available: http://legacy.library.ucsf.edu/tid/wwq52a99. Accessed 14 November 2011.
53. Garne D, Watson M, Chapman S, Byrne F (2005) Environmental tobacco smoke research published in the journal Indoor and Built Environment and associations with the tobacco industry. Lancet 365: 804-809.
54. Tong EK, England L, Glantz SA (2005) Changing conclusions on secondhand smoke in a sudden infant death syndrome review funded by the tobacco industry. Pediatrics 115: e356-366.
55. RJR, Colucci AV (1985) BYU ETS Project. 16 May 1985. Bates number 507915853. Available: http://legacy.library.ucsf.edu/tid/tig14d00. Accessed 14 November 2011.
56. R.J. Reynolds Tobacco Company (2000) R&D Scientific Presentations. 11 Aug 2000. Bates number 531630656/531630753. Available: http://legacy.library.ucsf.edu/tid/knp55a00. Accessed 14 November 2011.
57. Lowe JE, Moore KE (1987) Molecular biology of tumor promotion. Approved Ar #87-656. Internal # Fs 7067. 23 Dec 1987. Bates number 506857456. Available: http://legacy.library.ucsf.edu/tid/twf55a00. Accessed 14 November 2011.
58. Denoble VJ, Dragan YP, Kornfeld R, Mele PC, Naworal J, et al. (1986) Chronic nicotine administration. Sep 1986. Bates number 2047340356/2047340376. Available: http://legacy.library.ucsf.edu/tid/gyc36e00. Accessed 14 November 2011.
59. Philip Morris USA Research Center (1983) 1610 - Behavioral Pharmacology Annual Report 830000. 00 1983. Bates number 1003178534/1003178615. Available: http://legacy.library.ucsf.edu/tid/gpd74e00. Accessed 14 November 2011.
60. Philip Morris (1999) Publications from RD and E. 21 Apr 1999. Bates number 2079072311/2079072423. Available: http://legacy.library.ucsf.edu/tid/bjd47c00. Accessed 14 November 2011.
61. Allison J (2001) Fw: SAB Members. 09 Feb 2001. Bates number 2085523903. Available: http://legacy.library.ucsf.edu/tid/tcs10c00. Accessed 14 November 2011.
62. Barbara R (2007) Colloquia_1979-2007.pdf. 28 Feb 2007. Bates number 3063033715-3063033749. Available: http://legacy.library.ucsf.edu/tid/hqy91g00. Accessed 14 November 2011.

63. Smith G (1987) TAC Visit to Dr Barry Halliwoll University of London King's College 26th November 1987. 02 Dec 1987. Bates number 400959753-400959754. Available: http://legacy.library.ucsf.edu/tid/bge04a99. Accessed 14 November 2011.
64. Fisher A-L (2000) Risk Assessment Forum - First Meeting. 03 Nov 2000. Bates number 325381946. Available: http://legacy.library.ucsf.edu/tid/sry61a99. Accessed 14 November 2011.
65. Fisher A-L (2000) Risk Assessment Meeting - 30th November. 08 Nov 2000. Bates number 325381990-325381991. Available: http://legacy.library.ucsf.edu/tid/ssy61a99. Accessed 14 November 2011.
66. Council for Tobacco Research (1978) Microbiological Associates Contract 14-D July 1, 1977- June 30, 1978 - "the in vivo and in vitro analyses of the biological effects of smoke-related chemicals." 30 Jun 1978. Bates number 10007057-10007058. Available: http://legacy.library.ucsf.edu/tid/vjd66b00. Accessed 14 November 2011.
67. Henry C, Kouri R (1982) Associates M. Letter to Sheldon Sommers (Council for Tobacco Research). 27 Jan 1982. Bates number SF0828041/0828045. Available: http://legacy.library.ucsf.edu/tid/pfj10a00. Accessed 14 November 2011.
68. Council for Tobacco Research (1980) Publication by Grantee; interindividual variations of epoxide hydratase activity in human liver and lung biopsies, lymphocytes and fibroblast cultures; microsomes, drug oxidations, and chemical carcinogenesis. Vol. Ii. 18 Aug 1980. Bates number HK1361092/1361093. Available: http://legacy.library.ucsf.edu/tid/abm2aa00. Accessed 14 November 2011.
69. Muscholl E (Univ Mainz), Oesch F (Univ Mainz) (1978) Application for renewal of research grant importance of epoxide hydratase, dihydrodiol dehydrogenase and conjugating enzymes in the control of mutagenic and transforming metabolites of polycyclic hydrocarbons occurring in cigarette smoke and interindividual comparison of their activities in man. 28 Jun 1978. Bates number 50124698/50124707. Available: http://legacy.library.ucsf.edu/tid/sdh36d00. Accessed 14 November 2011.
70. Walk RA (1994) Visit by Dr. Mathuros Ruchirawat, Vice President for Research, Chulabhorn Research Institute (Cri), Bangkok, Thailand. 13 Jul 1994. Bates number 2062045574/2062045576. Available: http://legacy.library.ucsf.edu/tid/vzz43e00. Accessed 14 November 2011.
71. Saunders S, Steele H, Apoptosis Subcomm C (1999) Apoptosis Working Group Meeting Report. 18 Mar 1999. Bates number 521559705/521559706. Available: http://legacy.library.ucsf.edu/tid/vrp60d00. Accessed 14 November 2011.
72. Smith G (1990) International Symposium on Nicotine. Hamburg June 1990. 09 Jul 1990. Bates number 300522856-300522865. Available: http://legacy.library.ucsf.edu/tid/zks18a99. Accessed 14 November 2011.
73. Walk RA (4 Jul 1991) Bates number 2028698861. Available: http://legacy.library.ucsf.edu/tid/yck12e00. Accessed 14 November 2011.
74. Thornton R (1993) Research proposal. 10 Sep 1993. Bates number 401015140. Available: http://legacy.library.ucsf.edu/tid/kgr61a99. Accessed 14 November 2011.
75. Scientific Research Group (1998) Projects expenditure. 00 1998. Bates number 321727559. Available: http://legacy.library.ucsf.edu/tid/qrr44a99. Accessed 14 November 2011.
76. Rudge L (1994) Note from L Rudge to A Sarkar regarding the possibility of ITC sponsoring scientists to attend the International Cancer Congress. 06 May 1994. Bates number 500863892-500863894. Available: http://legacy.library.ucsf.edu/tid/qdh10a99. Accessed 14 November 2011.
77. Baker R (1999) British American Tobacco Scientific Research Group. 19 Aug 1999. Bates number 325284711-325284720. Available: http://legacy.library.ucsf.edu/tid/rcm82a99. Accessed 14 November 2011.
78. Carmines EL (1999) Evaluation of the potential effects of flavor ingredients added to cigarettes. Part 1. Cigarette design and testing. 10 Apr 1999. Bates number 2505330385/2505330415. Available: http://legacy.library.ucsf.edu/tid/mke25c00. Accessed 14 November 2011.
79. Winston N (1998) Product testing laboratory final laboratory averages report none - 97-1018 - extramural flavor study, activity number t00481 large scale. 19 Jan 1998. Bates number 2075175965/2075175968. Available: http://legacy.library.ucsf.edu/tid/iey56c00. Accessed 14 November 2011.
80. Nixon G (1998) Fax Project Mix. 29 Jan 1998. Bates number 2075175964. Available: http://legacy.library.ucsf.edu/tid/hey56c00. Accessed 14 November 2011.
81. Lee BC, Burns DM, Gairola CG, Harris JE, Hoffman D, et al. (1993) Toxicity testing plan: US Consumer Product Safety Commission in Consultation with the US Department of Health and Human Services. Washington (D.C.): US Department of Health and Human Services.
82. International Agency for Research on Cancer (1998) Tobacco smoking. Lyon, France: International Agency for Research on Cancer.
83. Solana R (1997) Chemistry list for product integrity testing. 18 Mar 1997. Bates number 2501778545. Available: http://legacy.library.ucsf.edu/tid/fpt93g00. Accessed 14 November 2011.
84. (1993) Draft smoke chemistry testing list based on Hoffman list, IARC and Sg lists, and Advisory Board recommendations. Bates number 2501778546. Available: http://legacy.library.ucsf.edu/tid/rqp93g00. Accessed 14 November 2011.
85. Agency for Toxic Substances & Disease Registry. Centers for Disease Control. Toxicity of polycyclic

aromatic hydrocarbons. Available: http://www.atsdr.cdc.gov/csem/pah/pah_physiologic-effects.html. Accessed November 23, 2010. [An archived copy of this page is available on the Internet Archive at http://web.archive.org/web/20110606032914/http://www.atsdr.cdc.gov/csem/pah/pah_physiologic-effects.html] Accessed 14 November 2011.
86. California Office of Environmental Health Hazard Assessment. (2001) Polycyclic organic matter. http://oehha.ca.gov/air/toxic_contaminants/pdf_zip/PAHs_Final.pdf. Accessed 14 November 2011.
87. Pelkonen O, Nebert DW (1982) Metabolism of polycyclic aromatic hydrocarbons: etiologic role in carcinogenesis. Pharmacol Rev 34: 189-222.
88. Philip Morris (1994) [Information on the standard design of our 21-day inhalation studies with cigarette smoke and on results obtained for URSUS-35]. 00 Sep 1994. Bates number 2501383813/2501383841. Available: http://legacy.library.ucsf.edu/tid/uhk42d00. Accessed 14 November 2011.
89. INBIFO/CRC (1999) Project Mix - chemical analysis of mainstream smoke. January 1999. Bates number 2505383586/2505383604. Available: http://legacy.library.ucsf.edu/tid/yfe39c00. Accessed 14 November 2011.
90. Gomm W, Reininghaus W, Rustemeier K, Vonholt K (1999) Report P 0500/3283. 04 Mar 1999. Bates number 2075165366/2075165429. Available: http://legacy.library.ucsf.edu/tid/tcb27d00. Accessed 14 November 2011.
91. RJ Reynolds (2009) Ammoniation. Bates number 509018864-509018865A. Available: http://legacy.library.ucsf.edu/tid/xxb66b00. Accessed 14 November 2011.
92. Stevenson T, Proctor R (2008) The secret and soul of Marlboro: Phillip Morris and the origins, spread, and denial of nicotine freebasing. Am J Public Health 98: 1184-1194.
93. Blevins RA, Jr. (1973) U.S Exhibit 20,669, Memo, re: Free nicotine as a difference in performance between brands, RAYMOND A. BLEVINS, JR., R.J. REYNOLDS TOBACCO CO., July 12, 1973. 12 Jul 1973. Bates number USX006703. Available: http://legacy.library.ucsf.edu/tid/acq36b00. Accessed 14 November 2011.
94. Rustemeier K (1998) Study plan amendment: P - 3283 Change-no.: 2 chemical analysis, mix. 09 Oct 1998. Bates number 2505104535/2505104536. Available: http://legacy.library.ucsf.edu/tid/pvw46c00. Accessed 14 November 2011.
95. Gomm W, Hackenberg U, Reininghaus W, Rustemeier K, Voncken P, et al. (1998) Study plan P 0500/3283 chemical analysis of mainstream smoke of cigarettes containing ingredient mixtures. 29 Jan 1998. Bates number 2501958943/2501958963. Available: http://legacy.library.ucsf.edu/tid/cnf81c00. Accessed 14 November 2011.
96. Reininghaus W (1998) Telefax message no. 2248. 12 Feb 1998. Bates number 2075175916. Available: http://legacy.library.ucsf.edu/tid/dey56c00. Accessed 14 November 2011.
97. Reininghaus W (1998) Report P - 3283 table mainstream smoke yields of research cigarettes. 12 Feb 1998. Bates number 2505104541/2505104544. Available: http://legacy.library.ucsf.edu/tid/uvw46c00. Accessed 14 November 2011.
98. USDHHS (1988) The health consequences of smoking: nicotine addiction. A report of the Surgeon General. Washington (D.C.): US Department of Health and Human Services, Public Health Service, Centers for Disease Control, Center for Health Promotion and Education, Office on Smoking and Health. DHHS Publication number (CDC) 88-8406, 1988 DHHS Publication number (CDC) 88-8406, 1988.
99. National Cancer Institute (2001) Tobacco control monograph 13: risks associated with smoking cigarettes with low machine-measured yeilds of tar and nicotine. Bethesda (Maryland): National Cancer Institute.
100. Benowitz N (2010) Nicotine addiction. N Engl J Med 362: 2295-2303.
101. Gomm W, Hackenberg U, Kaegler M, Kindt R, Kuhn D, et al. (1996) Study plan P 0500/3248 correlation of classic and mechanistic end points in a comparative tumorigenicity study on rats chronic effects of room-aged sidestream smoke and diesel engine exhaust (Pt). 09 Dec 1996. Bates number 2064276608/2064276713. Available: http://legacy.library.ucsf.edu/tid/yjo45d00. Accessed 14 November 2011.
102. Stinn W, Teredesai A, Anskeit E, Rustemeier K, Schepers G, et al. (2005) Chronic nose-only inhalation study in rats, comparing room-aged sidestream cigarette smoke and diesel engine exhaust Inhal Toxicol 17: 549-576.
103. Glantz SA (2005) Primer of biostatistics. San Francisco: McGraw-Hill.
104. Roemer E, Rustemeier K, Vanscheeuwijck PM, Meisgen TJ, Veltel DJ, et al. (2000) Effects of the addition of flavor ingredients to the tobacco on the chemical composition and biological activity of cigarette smoke. 00 Mar 2000. Bates number 98984120. Available: http://legacy.library.ucsf.edu/tid/xsy64d00. Accessed 14 November 2011.
105. Byrd III DM, editor (2004) Phase one: the feasibility of testing ingredients added to cigarettes. Bethesda (Maryland): Life Sciences Research Office.
106. Byrd III DM, Lewis KD, Nixon PM, Feldman RS, editors (2004) Phase two: scientific criteria for the evaluation of ingredients added to cigarettes. Bethesda (Maryland): Life Sciences Research Office, Inc.
107. van der Toorn M, Rezayat D, Kauffman HF, Bakker SJ, Gans RO, et al. (2009) Lipid-soluble components in cigarette smoke induce mitochondrial production of reactive oxygen species in lung epithelial cells. Am J Physiol Lung Cell Mol Physiol 297: L109-L114.
108. Sobus JR, McClean MD, Herrick RF, Waidyanatha S, Onyemauwa F, et al. (2009) Investigation of PAH biomarkers in the urine of workers exposed to hot asphalt. Ann Occup Hyg 53: 551-560.

109. Sobus JR, McClean MD, Herrick RF, Waidyanatha S, Nylander-French LA, et al. (2009) Comparing urinary biomarkers of airborne and dermal exposure to polycyclic aromatic compounds in asphalt-exposed workers. Ann Occup Hyg 53: 561-571.
110. Slebos DJ, Ryter SW, van der Toorn M, Liu F, Guo F, et al. (2007) Mitochondrial localization and function of heme oxygenase-1 in cigarette smoke-induced cell death. Am J Respir Cell Mol Biol 36: 409-417.
111. Serdar B, Egeghy PP, Waidyanatha S, Gibson R, Rappaport SM (2003) Urinary biomarkers of exposure to jet fuel (JP-8). Environ Health Perspect 111: 1760-1764.
112. Mutti A, Corradi M, Goldoni M, Vettori MV, Bernard A, et al. (2006) Exhaled metallic elements and serum pneumoproteins in asymptomatic smokers and patients with COPD or asthma. Chest 129: 1288-1297.
113. Kim D, Andersen ME, Chao YC, Egeghy PP, Rappaport SM, et al. (2007) PBTK modeling demonstrates contribution of dermal and inhalation exposure components to end-exhaled breath concentrations of naphthalene. Environ Health Perspect 115: 894-901.
114. Fowles J, Dybing E (2003) Application of toxicological risk assessment principles to the chemical constituents of cigarette smoke. Tob Control 12: 424-430.
115. Al Rashidi M, Shihadeh A, Saliba NA (2008) Volatile aldehydes in the mainstream smoke of the narghile waterpipe. Food Chem Toxicol 46: 3546-3549.
116. Moennikes O, Vanscheeuwijck PM, Friedrichs B, Anskeit E, Patskan GJ (2008) Reduced toxicological activity of cigarette smoke by the addition of ammonia magnesium phosphate to the paper of an electrically heated cigarette: subchronic inhalation toxicology. Inhal Toxicol 20: 647-663.
117. Solana RP, Stratton K (2000) Comment to IOM Question 3. 17 Apr 2000. Bates number 2082337716/2082337726. Available: http://legacy.library.ucsf.edu/tid/kim49c00. Accessed 14 November 2011.
118. Lemus R, Carmines EL, Van Miert E, Coggins CR, Anskeit E, et al. (2007) Toxicological comparisons of cigarettes containing different amounts of vanillin. Inhal Toxicol 19: 683-699.
119. Gaworski CL, Lemus-Olalde R, Carmines EL (2008) Toxicological evaluation of potassium sorbate added to cigarette tobacco. Food Chem Toxicol 46: 339-351.
120. Gaworski CL, Coggins CR, Carmines EL (2008) Palladium alters cigarette smoke toxicological profile, but accumulates in the lungs of rats during inhalation exposure. Inhal Toxicol 20: 167-182.
121. Carmines EL, Lemus R, Gaworski CL (2005) Toxicologic evaluation of licorice extract as a cigarette ingredient. Food Chem Toxicol 43: 1303-1322.
122. Carmines EL, Gaworski CL (2005) Toxicological evaluation of glycerin as a cigarette ingredient. Food Chem Toxicol 43: 1521-1539.
123. Institute of Medicine (2009) Secondhand smoke exposure and cardiovascular effects: making sense of the evidence. Washington (D.C.): National Academy Press. http://www.iom.edu/Reports/2009/Secondhand-Smoke-Exposure-and-Cardiovascular-Effects-Making-Sense-of-the-Evidence.aspx.
124. Pope CA, 3rd, Burnett RT, Krewski D, Jerrett M, Shi Y, et al. (2009) Cardiovascular mortality and exposure to airborne fine particulate matter and cigarette smoke: shape of the exposure-response relationship. Circulation 120: 941-948.
125. US Department of Health and Human Services (2010) How tobacco smoke causes disease: the biology and behavioral basis for smoking-attributable disease. A report of the Surgeon General. Washington (D.C.): Dept. of Health and Human Services, Public Health Service, Office of Surgeon General, 2010. Available: http://surgeongeneral.gov/library/tobaccosmoke/index.html.
126. Brook RD, Rajagopalan S, Pope CA, 3rd, Brook JR, Bhatnagar A, et al. (2010) Particulate matter air pollution and cardiovascular disease: an update to the scientific statement from the American Heart Association. Circulation 121: 2331-2378.
127. Ejaz S, Insan-ud-din, Ashraf M, Nawaz M, Lim C, et al. (2009) Cigarette smoke condensate and total particulate matter severely disrupts physiological angiogenesis. Food Chem Toxicol 47: 601-614.
128. Vleeming W, Schenk E, Opperhuizen A (2004) Letter to the Editor. Food and Chemical Toxicology 42: 865-866.
129. Carmines EL, Haussmann H-J, Rustemeier K, Roemer E, Stabber R (2004) Response to Letter. Food and Chemical Toxicology 42: 867.
130. Gomm W, Hackenberg U, Reininghaus W, Rustemeier K, Voncken P, et al. (1998) Study plan P 0500/3283 chemical analysis of mainstream smoke of cigarettes 97.Mg.292, 97.Mg.295, 97.Mg.296, and 97.Mj.125 to 97.Mj.128 Project Mix (Pt). 03 Feb 1998. Bates number 2502334950/2502334971. Available: http://legacy.library.ucsf.edu/tid/egh81c00. Accessed 14 November 2011.
131. INBIFO (1998) INBIFO testing index - by project number 23 Feb 1998. Bates number 2075175385/2075175389. Available: http://legacy.library.ucsf.edu/tid/ocy56c00. Accessed 14 November 2011.
132. (2000) Report P 0500/3316 Chemical analysis of mainstream smoke from cigarettes 98.fb.107, 98.fb.109, 98.fb.110, and 98.fb. 111 project juice. 06 Nov 2000. Bates number 2067592110-2067592176.
133. INBIFO (1999) Report P 0500/3315 Chemical analysis of mainstream smoke from cigarettes 98.fb.107, 98.fb.113, 98.fb.114, and 98.fb. 116 project milk. 16 Dec 1999. Bates number 2067620440-2067620462. Available: http://legacy.library.ucsf.edu/tid/pnb97g00. Accessed 14 November 2011.
134. INBIFO (2001) Study plan P 0500/3417 in vitro mutagenicity of total particulate matter of mainstream smoke from cigarettes with different amounts of propylene glycol in the filler salmonella typhimurium reverse mutation assay project pg Ii. 18 Sep 2001. Bates

number 2067119314/2067119338. Available: http://legacy.library.ucsf.edu/tid/nly34a00. Accessed 14 November 2011.
135. Roemer E (1996) Study concept chemical analyses of mainstream smoke of the standard reference cigarette 1r4f smoked various conditions. 26 Sep 1996. Bates number 2064300661/2064300663. Available: http://legacy.library.ucsf.edu/tid/awz50c00. Accessed 14 November 2011.
136. Roemer E (1996) Study concept in vitro cytotoxicity (mouse embryo balb/c 3t3 cells) of mainstream smoke fractions of the standard reference cigarette 1r4f smoked under various conditions. 26 Sep 1996. Bates number 2064300659/2064300660. Available: http://legacy.library.ucsf.edu/tid/bwz50c00. Accessed 14 November 2011.
137. Roemer E (1996) Study concept salmonella reverse mutation assay of total particulate matter of the standard reference cigarette 1r4f smoked under various conditions. 26 Sep 1996. Bates number 2064300657/2064300658. Available: http://legacy.library.ucsf.edu/tid/cwz50c00. Accessed 14 November 2011.
138. McCann J, Gold LS, Horn L, McGill R, Graedel TE, et al. (1988) Statistical analysis of Salmonella test data and comparison to results of animal cancer tests. Mutat Res 205: 183-195.
139. Parodi S, Taningher M, Romano P, Grilli S, Santi L (1990) Mutagenic and carcinogenic potency indices and their correlation. Teratog Carcinog Mutagen 10: 177-197.
140. Bogen KT (1995) Improved prediction of carcinogenic potencies from mutagenic potencies for chemicals positive in rodents and the ames test. environmental and molecular mutagenesis 25: 37-49.
141. Fetterman BA, Kim BS, Margolin BH, Schildcrout JS, Smith MG, et al. (1997) Predicting rodent carcinogenicity from mutagenic potency measured in the Ames Salmonella assay. Environ Mol Mutagen 29: 312-322.
142. Baker RR, Massey ED, Smith G (2004) An overview of the effects of tobacco ingredients on smoke chemistry and toxicity. Food Chem Toxicol 42 Suppl: S53-83.
143. Baker RR, Pereira da Silva JR, Smith G (2004) The effect of tobacco ingredients on smoke chemistry. Part II: casing ingredients. Food Chem Toxicol 42 Suppl: S39-52.
144. Baker RR, Pereira da Silva JR, Smith G (2004) The effect of tobacco ingredients on smoke chemistry. Part I: flavourings and additives. Food Chem Toxicol 42 Suppl: S3-37.
145. Neilsen K, Glantz SA (2004) A tobacco industry study of airline cabin air quality: dropping inconvenient findings. Tob Control 13 Suppl 1: i20-i29.
146. Yano E (2005) Japanese spousal smoking study revisited: how a tobacco industry funded paper reached erroneous conclusions. Tob Control 14: 227-233; discussion 233-225.
147. Barnes RL, Hammond SK, Glantz SA (2006) The tobacco industry's role in the 16 Cities Study of secondhand tobacco smoke: do the data support the stated conclusions? Environ Health Perspect 114: 1890-1897.
148. Barnes RL, Glantz SA (2007) Endotoxins in tobacco smoke: shifting tobacco industry positions. Nicotine Tob Res 9: 995-1004.
149. Tong EK, Glantz SA (2007) Tobacco industry efforts undermining evidence linking secondhand smoke with cardiovascular disease. Circulation 116: 1845-1854.
150. World Health Organization. Guidelines for implementation of Article 5.3 of the WHO Framework Convention on Tobacco Control on the protection of public health policies with respect to tobacco control from commercial and other vested interests of the tobacco industry. http://www.who.int/fctc/guidelines/article_5_3.pdf. Accessed 12 April 2011.
151. Yerger VB, McCandless PM (2011) Menthol sensory qualities and smoking topography: a revew of tobacco industry documents. Tobacco Control 20: ii37-ii43.
152. Yerger VB (2011) Menthol's potential effects on nicotine dependence: a tobacco industry perspective. Tob Control 20: ii29-ii36.

ZUSAMMENFASSUNG DES HERAUSGEBERS

Hintergrund

In den USA hatte die Tabakindustrie bereits im Jahr 1963 erkannt, dass eine Regulierung ihrer Produkte unabwendbar ist. Regulierungen durch die US-Kontrollbehörde zur Lebens- und Arzneimittel-Überwachung (US Food and Drug Administration, FDA), die 2009 schließlich rechtskräftig wurden, widmete die Industrie ab Mitte der 1990er Jahre gesteigerte Aufmerksamkeit. Zudem enthielt das Rahmenabkommen zur Tabakkontrolle (FCTC) der Weltgesundheits-organisation (WHO), das 2003 in Kraft trat, Bestimmungen, die auf die Inhaltstoffe von Tabakprodukten und deren Offenlegung abzielten. Obwohl diese Maßnahmen Fortschritte für die Tabakkontrolle darstellen, zeigen die Entwicklungen der vergangenen Jahrzehnte, wie entschlossen die Tabakindustrie versucht gesetzliche Bestimmungen, einschließlich zu Additiven, zu vermeiden. In den USA erkannten führende Mitarbeiter von Philip Morris die Unabwendbarkeit gesetzlicher Bestimmungen und versuchten Gesetzgebung und Regulierung zu beeinflussen, indem sie die interne Forschung neu ausrichteten und wissenschaftliche Untersuchungen durchführten, die dazu dienen sollten, jeden Vorschlag zur Regulierung zu verändern. Beispielsweise führte die Firma ein Projekt namens „MIX“ durch, eine Untersuchung der Inhaltsstoffe und der Toxizität von Rauch, der beim Abbrennen von Zigaretten entstand, die drei verschiedene Kombination von 333 Zigarettenadditiven enthielten, die in ihrer „Zusammenstellung der in gängigen, kommerziell hergestellten Zigaretten ähnelten“. Die daraus abgeleiteten vier Publikationen, die in der Fachzeitschrift *Food and Chemical Toxicology* im Januar 2002 erschienen, kamen zu dem Schluss, die Ergebnisse ergäben keine nennenswerten Auswirkungen von Zusatzstoffen auf die Toxizität von Zigaretten.

Wozu dient die vorliegende Untersuchung?

Die WHO, die FDA und entsprechende nationale Regulierungsbehörden weltweit sehen den Gebrauch von Zigarettenadditiven mit großer Besorgnis. Philip Morris benutzte die Daten aus Projekt MIX, um die Unbedenklichkeit einzelner Additive zu reklamieren. Andere Zigarettenhersteller unternahmen ähnliche Experimente, die vergleichbare Resultate ergaben. Die Autoren der vorliegenden Untersuchung werteten Dokumente aus, die als Folge von Haftungsklagen gegen die Tabakindustrie öffentlich zugänglich gemacht wurden. Die Untersuchung erstreckt sich auf die Entstehung und die Konzeption von Projekt MIX und unterwirft die Ergebnisse einer zusätzlichen Auswertung, um zu bewerten, wie glaubhaft die Schlussfolgerungen der Arbeiten sind, die in *Food and Chemical Toxicology* veröffentlichten wurden.

Wie gingen die Untersucher vor und was fanden sie?

Die Untersucher werteten systematisch Dokumente der Tabakindustrie aus, die in der *Legacy Tobacco Documents Library* der *University of California, San Francisco* aufgrund von Haftungsklagen zugänglich sind (derzeit etwa 60 Millionen Seiten). In einem sich wiederholendem Prozess des Suchens, Überprüfens und Verfeinerns wurden 500 relevante Dokumente identifiziert, die einer detaillierten Prüfung unterzogen wurden.

Die Untersucher fanden heraus, dass die Ergebnisse der ursprünglichen Analyse aus Projekt MIX in den veröffentlichten Arbeiten verschleiert wurden, indem die Ergebnisse im Verhältnis zum Rohkondensat (total particulate matter, TPM) dargestellt wurden. Als die Untersucher ihre eigene Analyse durchführten, indem sie die Ergebnisse bezogen auf eine Zigarette darstellten (wie es im Originalprotokoll von Projekt MIX festgelegt wurde), stellte sich heraus, dass 15 krebserregende Chemikalien um 20% angestiegen waren. Die Untersucher berichteten zudem, dass Philip Morris aus ungeklärten Gründen 19 von 51 getesteten Chemikalien bei der Darstellung der Ergebnisse stark unterbewertete; darunter befinden sich 9 Substanzen, die, bezogen auf eine Zigarette, im Rauch der additivbelasteten Zigaretten verglichen mit Kontroll-zigaretten erheblich erhöht waren.

Die Untersucher gingen der Möglichkeit nach, dass Projekt MIX nicht infolge fehlender Effekte, sondern aufgrund mangelnder Teststärke keine signifikanten Veränderungen der Toxizität der mit Additiven belasteten Zigaretten nachweisen konnte. Dazu wurden eigene statistische Berechnungen durchgeführt. Die Nach-berechnung legt nahe, dass bei besserer Teststärke ein wesentlich breiteres Spektrum an biologischen Effekten unter dem Einfluss der Additive zu Tage getreten wäre, als es in der von Philip Morris publizierten Arbeit dargelegt wurde. Dies führt zu der Annahme, dass die Arbeit das toxische Potential von Rauch und Additiven erheblich unterschätzt.

Die Untersucher deckten zudem auf, dass *Food and Chemical Toxicology,* die Fachzeitschrift in der die vier aus Projekt MIX abgeleiten Arbeiten veröffentlicht wurden, einen Herausgeber und 11 Mitglieder des internationalen Redaktionsbeirates mit Verbindungen zur Tabakindustrie aufwies. Der wissenschaftliche Leiter von Projekt MIX, Edward Carmines, beschrieb den Veröffentlichungsprozess als “inside job.” (Insider Tätigkeit)

Was bedeuten diese Befunde?

Diese Befunde zeigen, dass Forschungsergebnisse der Tabakindustrie zum Gebrauch von Zigarettenadditiven nicht für bare Münze genommen werden können: die Ergebnisse beweisen, dass Toxine im Zigarettenrauch erheblich zunehmen, wenn zu Zigaretten Additive hinzugefügt werden. Zudem hätten Untersuchungen mit besserer Teststärke wahrscheinlich ein wesentlich breiteres Spektrum nachteiliger biologischer Effekte nachgewiesen, die mit Additiven assoziiert sind, als dies in den von Philip Morris veröffentlichten Arbeiten der Fall ist. Dies berechtigt zur Annahme, dass die veröffentlichten Arbeiten das toxische Potential von Rauch und Additiven erheblich unterschätzen.

Zulassungsbehörden, einschließlich der FDA und vergleichbare Einrichtungen andernorts, welche die FCTC der WHO umsetzen, sollten eine eigene unabhängige Analyse der Daten aus Projekt MIX vornehmen, die, korrekt bewertet, als gesicherte Erkenntnis für eine Abschaffung der untersuchten Additive in Zigaretten (einschließlich Menthol) aus Gründen der öffentlichen Gesundheit gelten können.

ZUSÄTZLICHE INFORMATIONEN

Bitte greifen Sie auf folgende Websites mittels der Online Version dieser Zusammenfassung zu unter http://dx.doi.org/10.1371/journal.pmed.1001145

- Für die Richtlinie von *PLoS Medicin*e zur Veröffent-lichung von Arbeiten, die von der Tabakindustrie gefördert sind siehe http://www.plosmedicine.org/article/info:doi/10.1371/journal.pmed.1000237
- Die Weltgesundheitsorganisation (WHO) bietet Informationen zum Rahmenabkommen zur Tabakkontrolle (FCTC) http://www.who.int/fctc/
- Die Dokumente, die von den Untersuchern dieser Arbeit ausgewertet wurden, können eingesehen werden bei der Legacy Tobacco Documents Library http://legacy.library.ucsf.edu
