# Supplementary material for: The Toxic Effects of Cigarette Additives. Philip Morris' Project Mix Reconsidered: An Analysis of Documents Released through Litigation
Source: PLoS Med. 2011 Dec 20;8(12):e1001145. doi: 10.1371/journal.pmed.1001145 (PMC3243707; doi:10.1371/journal.pmed.1001145)
Supplement: Alternative Language Text S2 — German translation of Text S1 by TK. (PDF) [file pmed.1001145.s009.pdf]

Die toxische Wirkung von Zigarettenadditiven. Das Philip Morris Projekt Mix auf dem Prüfstand: eine Analyse von Dokumenten, die als Folge von Haftungsklagen herausgegeben wurden

Ergänzender Text

Marcia S. Wertz, PhD RN

Thomas Kyriss, MD

Suman Paranjape, PhD, MPH

Stanton A. Glantz, PhD

PLoS Medicine 8(12): e1001145 doi: 10.1371/journal.pmed.1001145

**Tabelle S-1. Kombinierte Liste für die Analyse von Rauchinhaltsstoffen der CPSC, IARC und aus Projekt MIX**		
1,1-Dimethylhydrazin	Benzo[c]phenanthren	NAB *
1,3-Butadien (µg/cig) *	Benzo]c]pyren	Naphthalin†
1,4-Dimethylphenanthren	Benzo[ghi]perylen†	NAT *
1-Methylchrysen	Bernsteinsäure	NDBA
1-Methylphenanthren	Blei (ng/cig) *	NDEA
1-Naphthylamin	Cadmium *	NDELA *
2,3-dimethylmaleicanhydrtit	Captan	NDMA *
2-Cresol	Carbazol	NDPA
2-Methylchrysen	Catechol *	NEMA
2-Methylfluoranthen	Cholesterin	Nickel *
2-Nnaphthylamin	Chrom *	Nikotin *
2-Nitropropan	Chrysen *	N-Nitroso-N-methylethylamin
3- und 4-Kresol	Coronen	NNK *
3- und 4-Methylkatechol	Crotonaldehyd	NNN *
3-Methylchrysen	Cumarin	N-phenyl-2-naphthylamin
3-Methylfluoranthen	Cyanwasserstoff *	NPIP
3-Methylpyridin	DDT	NPY *
4-Aminobiphenyl	Dibenz [*a*,*h*] acridin	Ortho-anisidin
4-Methylchrysen	Dibenz [a,j] acridin *	O-cresol†
5-Methylchrysen *	Dibenz[-]anthracen *	O-toluidine
6-Methylchrysen	Dibenzo [a,e]pyren	P-cresol†
7H-Dibenzo[c,g]carbazol	Dibenzo [a,h]pyren	Perylen
Acenaphthen†	Dibenzo [a,i]pyren	pH Wert
Acenaphthylen†	Dibenzo[a,e]fluoranthen	Phenanthren *
Acetaldehyd *	Endrin	Phenol *
Acetamid	Essisäure	Propionaldehyd
Aceton	Ethylenoxid	Propylenoxid
Akrolein *	Eugenol	Pyren *
Akrylonitril *	Fluoranthen *	Pyridin
Ameisensäure	Fluoren *	Redox Potential
Ammoniak‡	Formaldehyd *	Resorcin *
Anilin	Hydrazin	Selen
Anthanthren	Hydroquinon *	Stickstoffoxide *
Anthracen *	Indeno [1,2,3-cd]pyren *	Styrol
Arsen *	Isopren (µg/cig) *	Teer
Benz[a]acridin	Kohlenmonoxid *	Toluol *
Benz[a]anthracen *	Malathion	TPM (Rohkondensat)*
Benz[c]acridin	Maleinsäureanhydrid	Triphenylen
Benzen *	Maleisäurehydrazid	Urethan
Benzo[-] fluoren	M-Cresol†	Vinylchlorid *
Benzo[-]fluoranthen *	Methanol	Wasser *
Benzo[a]pyren *	Methylacrylat	Y-butyrolactose
*Eingeschlossen in Projekt MIX und den Listen der CSPC oder IARC<br>†Eingeschlossen in Projekt MIX aber nicht in den Listen der CSPS oder IARC<br>‡Eingeschlossen in Projekt MIX, aber nicht publiziert [1]		

**Table S-2. 19 Rauchinhaltsstoffe. die nicht in den veröffentlichten Radarplots aufgeführt sind** [1]		
Acenaphthalen N* IG2 & IG3	Acenaphthen N IG2 & IG3	Fluoren N IG2
Phenanthren H IG1 & IG3 N IG2	Anthracen H IG1 & IG3 N IG2	Fluoranthen H IG1 & IG3
Pyren H IG1 & IG3	Benz[a]anthrazen H IG1 & IG3	Chrysen H IG1 & IG3
Benzo[-]fluoranthen H in allen Gruppen	Indeno [1,2,3-cd]pyren H IG1	Dibenz[-]anthracen
Benzo[ghi]perylen H in allen Gruppen	5-Methylchrysen	Dibenz[a,j]acridin
NDMA	NDELA	Chrom
Nickel		
*zeigt an, dass der Wert im additivhaltigen Rauch statistisch signifikant verändert ist verglichen mit dem Rauch der Kontrollzigarette bezogen auf eine Zigarette (N = niedriger als in Kontrollzigaretten, H = höher als Kontrolle, IG = Zutaten Gruppe) mitgeteilt in der von Rustemeier [1] veröffentlichten Tabelle von 2002.		

**Table S-3. Statistisch signifikante toxikologische Veränderungen durch Zigarettenrauch der drei Additivgruppen mit je zwei unterschiedlichen Dosierungen (Nur statistisch signifikante Ergebnisse sind abgebildet.)**				
**Biologische Parameter**	**Veröffentlichte Auswertung basierend auf den beobachteten Daten** [2] **(N=4-14 pro Gruppe†)**		**Bei Annahme der selben Durchschnittswerte und Standardabweichungen, aber mit N=50 pro Gruppe**	
	**Männliche Ratten**	**Weibliche Ratten**	**Männliche Ratten**	**Weibliche Ratten**
Anteil des Carboxyhämoglobin im Blut (%)			Angestiegen 1N<br>Gefallen 2N 1H	Gefallen in allen Gruppen
Nikotinmetaboliten* im Urin (%) NCOT			Angestiegen 1N<br>Gefallen 1H	Angestiegen 1N 2N 3H
**Serumchemie**				
Alkalische Phosphatase			Gefallen 2N	Angestiegen 2H
Aspartataminotransferase (AST)				Angestiegen 2H<br>Gefallen 3H
Alaninaminotransferase (ALT)			Gefallen 1H 2H	Angestiegen 2H<br>Gefallen 3N 3H
γGlutamyltransferase (GGT)				Angestiegen 1N 2N 2H 3N
Gesamtcholesterin	Angestiegen 2N 3N 3H		Angestiegen 1N 2N 2H 3N 3H	Angestiegen 3N
Triglyzeride				Angestiegen 1N 2N 2H 3N
Glukose				Gefallen 1H 2N 2H 3N
Harnstoff	Gefallen 3N 3H	Angestiegen 1H 2N	Gefallen in allen Gruppen	Angestiegen in allen Gruppen
**Hämatologie**				
Erythrozyten			Angestiegen 2N 3N 3H	Angestiegen 1H 2H 3N
Hämatokrit			Angestiegen 1H 2N 3N	Angestiegen 1H 2H 3N
Hämoglobin			Angestiegen 1H 2N 2H 3N 3H	Angestiegen 2H 3N
Thrombozyten			Angestiegen 3H	Angestiegen 1N 2N 3N 3H
Leukozyten			Angestiegen 1N 2H	Angestiegen 1N 2H
Neutrophile				Gefallen 3H
Lymphozyten			Angestiegen 1N 2H 3N 3H	Gefallen 1N 2H
**Organgewichte**				
Thymus	Angestiegen 1N 1H 2H 3N 3H	Angestiegen 3H	Angestiegen in allen Gruppen	Angestiegen 3H<br>Gefallen 1N
Leber	Angestiegen 2H		Angestiegen 2H 3H	Angestiegen 2N
Nebennieren				Angestiegen 1N 2N 2H
Lungen	Angestiegen 2H		Angestiegen 1H 2N	Angestiegen 1N 1H 2N 3H
Nieren				
**Histopathologie der Nase**				
Nasenhöhle Level 1				
Hyperplasie von Respir.epithel		Angestiegen 1N	Gefallen 1N 1H 2H	Angestiegen 1N 1H 2N 2H 3N

und der Reservezellen				
Plattenepithelhyperplasie				Angestiegen 3H<br>Gefallen 2H
Gobletzellhyperplasie				Angestiegen 3H
Verlust von Gobletzellen			Angestiegen 1N 1H 2N	Gefallen 2N 2H 3N 3H
Nasenhöhle Level 2				
Hyperplasie von Respir.epithel und der Reservezellen				Angestiegen 2H 3H
Atrophie der Riechschleimhaut	Gefallen 2H	Angestiegen 2N 2H	Gefallen 1N 2H 3N	Angestiegen 1N 1H 2N 2H 3N
Plattenepithelhyperplasie				Angestiegen 2H
Nasenhöhle Level 3				
Atrophie der Riechschleimhaut		Angestiegen 2N 2H	Gefallen 2H 3N	Angestiegen 1N 1H 2N 2H 3N
Plattenepithelhyperplasie		Angestiegen 2H	Gefallen 2H	Angestiegen 1N 1H 2N 2H 3N
Nasenhöhle Level 4				
Atrophie der Riechschleimhaut				Angestiegen 1N 2N
Plattenepithelhyperplasie			Gefallen 2H	Angestiegen 1N 2H
**Histopathologie des Larynx**				
Epiglottis				
Metaplasie des mehrreihigen Flimmerepithels			Angestiegen 2N 2H	Gefallen 1H 3N 3H
Plattenepithelhyperplasie			Angestiegen 1H 2N 2H 3N	Angestiegen 1H
Larynxgrund (Aryknorpel)				
Metaplasie des mehrreihigen Flimmerepithels		Gefallen 3H		Gefallen in allen Gruppen
Stimmbänder, Mitte medial				
Plattenepithelhyperplasie			Angestiegen 1N 3H	Gefallen 1H 3N 3H
Stimmbänder, oben medial				
Metaplasie des mehrreihigen Flimmerepithels				
Stimmfalten				
Plattenepithelhyperplasie				Gefallen 1H 2N 2H
**Dicke des Larynxepithels**				
Lanryxgrund	Angestiegen 3N	Angestiegen 3N 3H	Angestiegen 2N 2H 3N	Gefallen 1H 3N 3H
**Histopathologie der Trachea, Bronchien and Lungen**				
Trachealbifurkation				
Hyperplasie von Respir.epithel und Gobletzellen			Angestiegen 2N	Gefallen 3H
Rechte Lung				
Alveolarmakrophagen			Angestiegen 3H	Angestiegen 3H

				Gefallen 2H
Linke Lunge				
Hyperplasie von Respir.epithel und Gobletzellen			Angestiegen 2H	Gefallen 3H
Alveolarmakrophagen			Angestiegen 3H<br>Gefallen 2H	

†N variierte (z.B.4-5 Ratten pro Gruppe für HbCO%, 8 Ratten pro Gruppe für Nikotinemetabolite, 8-10 Ratten pro Gruppe für Serumchemie, Hämatologie und pathologische Untersuchungen, ausgenommen 14 Ratten in allen Kontrollgruppen).
Nummer entspricht der *Zutaten Gruppe* (z.B. 2 = *Zutaten Gruppe 2*); N oder H beziehen sich auf niedrige oder hohe Dosierungen der Additive in den Gruppen.
Nur von PM Wissenschaftlern [2] mitgeteilte statistisch signifikante Unterschiede (erste zwei Spalten, analytische Methodik entspricht der Publikation von PM) und die Re-Analyse der Autoren unter der Annahme dass die selben Durchschnittswerte und Standardabweichungen wie von PM mitgeteilt, aber eine angehobene Stichprobengröße von 50 Ratten pro Gruppe vorliegen (letzte zwei Spalten)
Ein leeres Feld zeigt statistisch nicht signifikante Unterschiede an.
*Zigaretten mit Additiven hatten angeblich weniger Tabak (ersetzt durch Additive), und daher weniger Nikotin, was geringere Cotininspiegel erklären kann, sofern bestimmt.

LITERATUR

1. Rustemeier K, Stabbert R, Haussmann HJ, Roemer E, Carmines EL (2002) Evaluation of the potential effects of ingredients added to cigarettes. Part 2: chemical composition of mainstream smoke. Food Chem Toxicol 40: 93-104.
2. Vanscheeuwijck PM, Teredesai A, Terpstra PM, Verbeeck J, Kuhl P, et al. (2002) Evaluation of the potential effects of ingredients added to cigarettes. Part 4: subchronic inhalation toxicity. Food Chem Toxicol 40: 113-131.
